# Single nuclear spin detection and control in a van der Waals material

**DOI:** 10.1038/s41586-025-09258-7

**Published:** 2025-07-09

**Authors:** Xingyu Gao, Sumukh Vaidya, Kejun Li, Zhun Ge, Saakshi Dikshit, Shimin Zhang, Peng Ju, Kunhong Shen, Yuanbin Jin, Yuan Ping, Tongcang Li

**Affiliations:** 1 Department of Physics and Astronomy, Purdue University, West Lafayette, IN USA; 2 Department of Physics, University of California, Santa Cruz, Santa Cruz, CA USA; 3 Department of Materials Science and Engineering, University of Wisconsin–Madison, Madison, WI USA; 4 Elmore Family School of Electrical and Computer Engineering, Purdue University, West Lafayette, IN USA; 5 Department of Physics, University of Wisconsin–Madison, Madison, WI USA; 6 Department of Chemistry, University of Wisconsin–Madison, Madison, WI USA; 7 Purdue Quantum Science and Engineering Institute, Purdue University, West Lafayette, IN USA; 8 Birck Nanotechnology Center, Purdue University, West Lafayette, IN USA

**Keywords:** Two-dimensional materials, Quantum metrology, Two-dimensional materials, Single photons and quantum effects

## Abstract

Optically active spin defects in solids^1,2^ are leading candidates for quantum sensing^3,4^ and quantum networking^5,6^. Recently, single spin defects were discovered in hexagonal boron nitride (hBN)^7–11^, a layered van der Waals (vdW) material. Owing to its two-dimensional structure, hBN allows spin defects to be positioned closer to target samples than in three-dimensional crystals, making it ideal for atomic-scale quantum sensing^12^, including nuclear magnetic resonance (NMR) of single molecules. However, the chemical structures of these defects^7–11^ remain unknown and detecting a single nuclear spin with a hBN spin defect has been elusive. Here we report the creation of single spin defects in hBN using ^13^C ion implantation and the identification of three distinct defect types based on hyperfine interactions. We observed both *S* = 1/2 and *S* = 1 spin states within a single hBN spin defect. We demonstrated atomic-scale NMR and coherent control of individual nuclear spins in a vdW material, with a π-gate fidelity up to 99.75% at room temperature. By comparing experimental results with density functional theory (DFT) calculations, we propose chemical structures for these spin defects. Our work advances the understanding of single spin defects in hBN and provides a pathway to enhance quantum sensing using hBN spin defects with nuclear spins as quantum memories.

## Abstract (web summary)

The detection and coherent control of single ^13^C nuclear spins in hexagonal boron nitride at room temperature enables the use of van der Waals materials in upcoming quantum technologies.

## Main

Solid-state spin defects have become a leading platform for a wide range of quantum technologies, including multinode quantum networking^5,6^ and quantum-enhanced sensing^3,4^. These advances are largely driven by the spin–photon quantum interfaces, which use optically addressable coherent spins. Despite the success of various spin–photon systems, each material platform has intrinsic limitations, creating trade-offs depending on the specific application^1,2^. Moreover, spin defects that operate at room temperature are rare. Recently, optically active spin defects in hBN, a vdW material, has gained vast attention^7–11,13^. The layered structure of hBN facilitates integration with nanophotonic devices^14^ and provides an ideal platform for quantum sensing at the atomic scale^12^. hBN spin defects have been used for sensing magnetic fields^15–17^, temperature^15,16^, strain^18^ and beyond^19^. However, electron spins in hBN suffer from short spin coherence times^20–22^.

Nuclear spins typically exhibit long coherence times thanks to their weak coupling with the local environment, making them ideal candidates for quantum registers^23^. By using nuclear spins as ancillary qubits, we can overcome the limitation of electron spin coherence times and enhance the sensitivity of a spin-based quantum sensor^24,25^. This approach requires the ability to initialize, control and read out individual nuclear spins^26,27^. In the context of hBN spin defects, previous experiments used negatively charged boron vacancy (${V}_{{\rm{B}}}^{-}$) defects to polarize and read out nitrogen nuclear spin ensembles^28–31^. However, these experiments were limited to ensemble-level operations owing to the low quantum efficiency of ${V}_{{\rm{B}}}^{-}$ defects. Moreover, the relatively short relaxation time of ${V}_{{\rm{B}}}^{-}$ electron spins (*T*_1_ < 20 μs at room temperature) restricted the maximum operational time of nuclear spins. Recently discovered single spin defects in hBN^7–11^ enabled the readout of individual electron spins in hBN. However, the chemical structures of these single spin defects remain unidentified and the control of individual nuclear spins in hBN or other vdW materials is still elusive.

Here we report the realization of single nuclear spin detection and control using a carbon-related defect in hBN. Our study reveals three main types of single spin defect in ^13^C-implanted hBN, with remarkably high optically detected magnetic resonance (ODMR) contrasts of up to 200%. Two of these defect types are strongly coupled to nearby ^13^C nuclear spins. We observe the coexistence of *S* = 1 and *S* = 1/2 states within a single spin defect, although only the *S* = 1/2 states exhibit strong coupling to ^13^C nuclear spins, with coupling strengths reaching up to 300 MHz. The strong hyperfine coupling results in well-resolved hyperfine structures, enabling the initialization and readout of single nuclear spins assisted by a defect electron spin. We realize electron–nuclear spin two-qubit gate and coherent control of a ^13^C nuclear spin, with a nuclear spin π-gate fidelity up to 99.75% at room temperature. We also perform Ramsey and Hahn echo measurements of the nuclear spin. The dephasing and coherence times of a typical ^13^C nuclear spin in hBN at room temperature are measured to be ${T}_{2}^{* }\,=\,$ 16.6 μs and *T*_2_ = 162 μs, respectively. Our DFT calculations suggest that ${{\rm{C}}}_{{\rm{B}}}^{+}{{\rm{C}}}_{{\rm{N}}}^{0}$ donor–acceptor pairs (DAP) and C_B_O_N_ are likely key components for the two types of spin defect with large hyperfine interactions.

## Single spin defects in ^13^C-implanted hBN

**Fig. 1:**
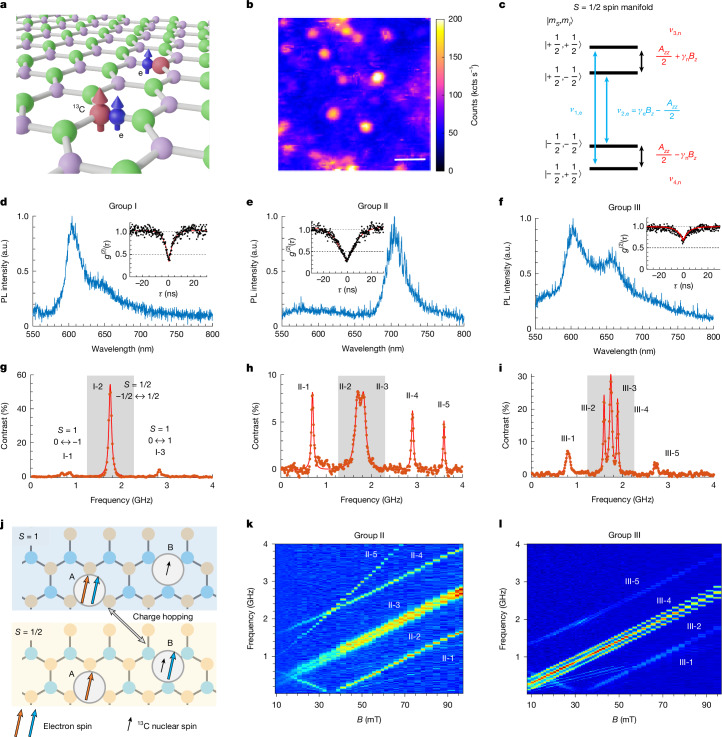
Observation of three types of single spin defect in hBN. **a**, Illustration of a carbon-related spin defect complex, consisting of an electron spin strongly coupled to a ^13^C nuclear spin and a nearby, weakly coupled electron spin without strong hyperfine interaction. **b**, PL confocal map showing isolated bright emitters in hBN. Scale bar, 2 μm. **c**, Energy-level diagram of an electron spin *S* = 1/2, coupled to a ^13^C nuclear spin (*I* = 1/2). *A*_*zz*_ is the hyperfine interaction strength. *ν*_1,e_ and *ν*_2,e_ are the two electron spin transitions. **d**–**f**, Optical spectra of defects 1–3, belonging to groups I–III, respectively. **g**–**i**, ODMR spectra of defects 1–3 under an out-of-plane external magnetic field of 62.5 mT. The number of peaks in the central region (shaded area) differs among groups I–III defects. Within this shaded area, the peaks correspond to the |−1/2⟩ ↔ |+1/2⟩ transitions, with hyperfine structures observed in groups II and III. Outside the shaded region, the transitions correspond to the |0⟩ ↔ |±1⟩ transitions. Furthermore, the defect in **h** exhibits a |−1⟩ ↔ |+1⟩ double-quantum transition (II-5). **j**, Illustration of the spin-pair model for explaining the coexistence of *S* = 1 and *S* = 1/2 transitions. **k**,**l**, Magnetic-field-dependent ODMR spectra of defect 2 in group II (**k**) and defect 3 in group III (**l**). a.u., arbitrary units. Source Data

We create carbon-related spin defects in hBN^8,9,11^ (Fig. 1) by ^13^CO_2_ (99% ^13^C) ion implantation and thermal annealing (Supplementary Information Section II). A confocal photoluminescence (PL) map reveals isolated emitters, as shown in Fig. 1b. Their optical spectra spread from 570 to 700 nm, depending on different defects (Fig. 1d–f). Photon-correlation measurements suggest that some defects are single photon emitters (*g*^(2)^(0) < 0.5). To verify their spin properties, we perform ODMR measurements by toggling the microwave on and off while collecting the emitted photons. The ODMR contrast *C* is determined by the ratio of photon count rates when the microwave is on (*N*_on_) or off (*N*_off_): *C* = (*N*_on_ − *N*_off_)/*N*_off_ × 100%. In contrast to previous reports of *S* = 1/2 spin defects with only a single resonant peak in ODMR^7–10^, we observe three distinct ODMR spectra with several resonances (Fig. 1g–i). The positive contrast indicates that the defects are initialized into a darker state and we also observe defects with negative contrasts but are rarer (Supplementary Fig. 20). These ODMR spectra feature a centre branch (shaded areas in Fig. 1g–i) comprising several resonances that are assigned to hyperfine structures, along with further side resonances located approximately 1 GHz away from the centre branch. The high ODMR contrast and brightness yield a typical DC magnetic field sensitivity of $5\,\mu {\rm{T}}/\sqrt{{\rm{Hz}}}$ (Methods).

On the basis of ODMR spectra as shown in Fig. 1g–i, we categorize the spin defects into three main groups, groups I–III, according to the number of peaks in the centre branch. Individual peaks are labelled according to their respective defect group. The hyperfine splitting of group II and III defects are determined to be 130 and 300 MHz, respectively. Notably, the 300 MHz hyperfine splitting is determined by the separation between transitions III-2 and III-4. The extra centre peak, III-3, exhibits a different hyperfine structure under weak microwave driving, suggesting that it originates from a second electron spin. This observation supports the recently proposed spin-pair model, in which a single defect complex hosts two electron spins, separated by several nanometres, that couple differently to the ^13^C nuclear spins^32,33^ (Fig. 1j).

To better understand the spin transitions, we measure the ODMR spectra as a function of out-of-plane magnetic fields (Fig. 1k,l). The results reveal an absence of zero-field splitting (ZFS) in the centre branch, corresponding to the |*m*_*S*_ = +1/2⟩ ↔ |*m*_*S*_ = −1/2⟩ transition within the *S* = 1/2 spin manifold (see Supplementary Information Section IV for experimental verification). By contrast, the side resonances (II-1, II-4, III-1 and III-5) exhibit non-zero ZFS and disperse with a *g*-factor of 2, confirming that they correspond to the |*m*_*S*_ = 0⟩ ↔ |*m*_*S*_ = ±1⟩ transitions within the *S* = 1 spin manifold. The Hamiltonian of each spin manifold is given by:

1 $$H=D{S}_{z}^{2}+E({S}_{x}^{2}-{S}_{y}^{2})+{\gamma }_{{\rm{e}}}{\bf{B}}\cdot {\bf{S}}+\sum _{i}{\bf{S}}\cdot {A}_{i}\cdot {{\bf{I}}}_{i}+{\gamma }_{{\rm{n}}}{\bf{B}}\cdot {{\bf{I}}}_{i},$$

in which *γ*_e_ and *γ*_n_ are the electron spin and the nuclear spin gyromagnetic ratios, respectively, and **B** is the external magnetic field. **S** denotes the electronic spin operator with *S* = 1 or 1/2. *D* and *E* together are the ZFS parameters, which are non-zero for the *S* = 1 states but vanish for the *S* = 1/2 states. **I**_*i*_ denotes the nuclear spin operator for the *i*th nuclear spin coupled to the defect electron. Specifically, *I* = 1/2 for ^13^C nuclear spins, *I* = 1 for ^14^N nuclear spins and *I* = 3/2 for ^11^B nuclear spins. The hyperfine interaction between the electron spin and the *i*th nuclear spin is described by the interaction tensor *A*_*i*_.

The magnetic-field-dependent ODMR allows us to extract the ZFS parameters *D* (typically  about 1 GHz) and *E* (varies from 100 to 400 MHz) along an out-of-plane quantization axis for the *S* = 1 transitions (Supplementary Fig. 3). This is different from the recent observation of a spin-triplet defect with an in-plane quantization axis^11^, making our system more favourable for magnetic-field alignment. Furthermore, an extra peak II-5 of defect 2 locates at the frequency *ν*_5_ = *ν*_1_ ± *ν*_4_ and has a level anticrossing with II-4 at 37 mT (Supplementary Fig. 3), suggesting a double-quantum transition. Although previously proposed spin-pair models based on DAP have focused only on *S* = 1/2 transitions in metastable state^32,33^, the coexistence of both *S* = 1/2 and *S* = 1 transitions can be well explained by an extended spin-pair model incorporating extra energy levels and transitions (see Methods). The DAP model also accounts for the large variation in PL spectra observed across different defects, despite their similar ODMR signatures^34,35^.

## Detection of single ^13^C nuclear spins in hBN

**Fig. 2:**
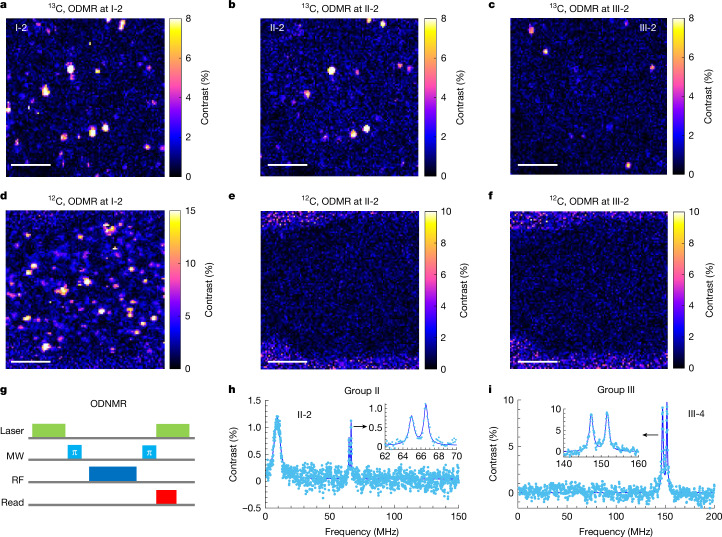
Optical detection of ^13^C nuclear spins in hBN. **a**–**c**, ODMR contrast map of ^13^C-implanted hBN by driving a microwave at: 2.01 GHz, resonance I-2 of group I defects (**a**); 1.95 GHz, resonance II-2 of group II defects (**b**); and 1.86 GHz, resonance III-2 of group III defects (**c**). **d**–**f**, ODMR contrast map of ^12^C-implanted hBN by driving the microwave at: I-2 (**d**), II-2 (**e**) and III-2 (**f**). The contrast fluctuation outside the hBN is caused by the low photon counts collected from the background. Scale bars, 5 μm. A 71.5-mT magnetic field is applied out of plane (perpendicular to the hBN nanosheet). **g**, Illustration of the ODNMR sequence. **h**, An ODNMR spectrum of defect 2 by driving the microwave at II-2. **i**, An ODNMR spectrum of defect 3 by driving the microwave at III-4. Source Data

The well-resolved hyperfine structures enable us to distinguish different defect groups through two-dimensional mapping of the ODMR contrast at specific microwave driving frequencies. Figure 2a–c presents the ODMR contrast distribution of a ^13^C-implanted hBN with microwaves resonant at I-2, II-2 and III-2 transitions (Fig. 1g–i), respectively. All three maps reveal several spots with finite contrasts, enabling us to locate group II and III defects. By contrast, no ODMR signal is observed in ^12^C-implanted hBN when microwaves are applied at the II-2 and III-2 transitions.

Besides ODMR, optically detected nuclear magnetic resonance (ODNMR) (Fig. 2g) of ^13^C nuclear spins can provide further insights into the electron–nuclear hyperfine coupling. Here we conduct these measurements at the centre branch of group II and III defects. For both defects 2 and 3, we observe two closely spaced resonances in ODNMR at approximately half the frequencies of the hyperfine splitting observed in ODMR (Fig. 2h,i). Such a feature verifies the *S* = 1/2 configuration of the defect electron spin for these transitions. For a *S* = 1 configuration, we expect the ODNMR resonance frequency to be the same as the hyperfine splitting in ODMR. The two-peak structure originates from the external magnetic field and/or a weaker hyperfine coupling to a second electronic spin. We also notice that different nuclear resonance frequencies are obtained when the microwave is applied at III-2 (III-4) and III-3 (Supplementary Fig. 17). These features further verify that two *S* = 1/2 electron spins are involved in defect 3.

## Coherent control of a ^13^C nuclear spin in hBN

The well-isolated electron spin transitions at III-2 and III-4 (Fig. 1i) enable coherent manipulation of individual electron spin states associated with different nuclear spin states. This capability allows for the initialization, control and readout of a nuclear spin by means of the electron spin, without the need for strong magnetic fields or level anticrossing^28–31^.

**Fig. 3:**
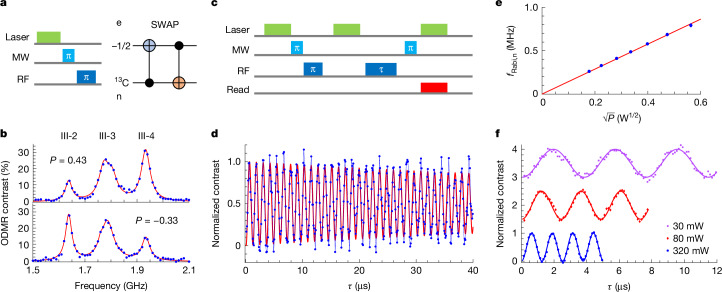
Initialization and coherent control of a ^13^C nuclear spin. **a**, Illustration of the pulse sequence (left) for nuclear spin initialization. We use a SWAP gate (right) to transfer the electron spin polarization to the ^13^C nuclear spin. **b**, ODMR signal after nuclear spin initialization. One of the two peaks (III-2 and III-4) dominates, yielding a nuclear spin polarization of 0.43 (top) and −0.33 (bottom), depending on the direction of initialization. **c**, Pulse sequence for nuclear spin coherent control. **d**, An example of nuclear spin Rabi oscillation, persisting for 40 μs without significant decay. The blue curve is the experimental data and the red curve is fitting. **e**, Nuclear spin Rabi frequency as a function of the square root of the RF power. **f**, Nuclear spin Rabi oscillations taken at different RF powers. Source Data

To polarize the ^13^C nuclear spin, we first use a laser pulse to initialize the electron state (assuming |*m*_*S*_ = −1/2⟩). Next, we apply a SWAP gate (Fig. 3a) to swap the electron spin state and nuclear spin state, after which the nuclear spin is initialized to |↑⟩ (or |↓⟩). To estimate the fidelity of nuclear spin initialization, we measure the electron spin ODMR signal after the SWAP gate and calculate the polarization according to the imbalance between III-2 and III-4 (Fig. 3b). We determine the nuclear spin polarization to be approximately 43% (−33%) using the equation *P* = (*ρ*_4_ − *ρ*_2_)/(*ρ*_4_ + *ρ*_2_), in which *ρ*_*j*_ represents the amplitude of resonance III-*j*. A higher nuclear spin polarization of 60% can be achieved with another defect, as shown in Supplementary Fig. 18.

For nuclear spin coherent control, we use the protocol depicted in Fig. 3c. After polarizing the nuclear spin, another 10-μs laser pulse reinitializes the electron spin state. Subsequently, we park the frequency of the selective RF pulse while varying its pulse duration *τ*. Finally, the nuclear spin state is read out by means of the spin defect using a selective microwave π pulse and a 5-μs laser pulse. Figure 3d shows the resulting nuclear spin Rabi oscillations. We determine a π-pulse time of 0.6 μs and a coherence time of 117 μs by fitting the oscillation contrast *C*(*τ*) to *C*(*τ*) = *a*sin(π*τ*/*T*_π_ + *b*)exp(−*τ*/*T*_Rabi_) + *d*, yielding a π-gate fidelity of 99.75% (Methods). The extended operational time for nuclear spins is attributed to the long electron spin relaxation time (*T*_1,e_ = 144 μs; Supplementary Fig. 15) of this carbon-related spin defect, which is an order of magnitude longer than that of ${V}_{{\rm{B}}}^{-}$ spin ensembles^28,29^. By repeating the measurements at different RF powers, we observe a clear power dependence of the oscillations (Fig. 3f), for which the nuclear Rabi frequency is linearly proportional to the amplitude of the RF field (Fig. 3e).

**Fig. 4:**
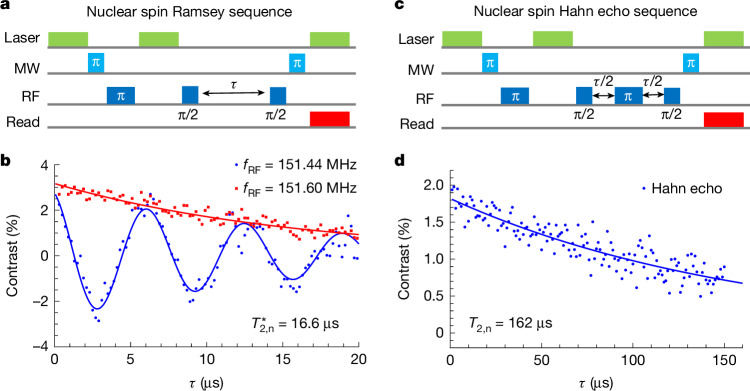
Spin coherence of a ^13^C nuclear spin. **a**, The pulse sequence of nuclear spin Ramsey interferometry. **b**, The nuclear spin Ramsey fringe. The measurements are performed when the RF frequency is in resonance with the nuclear spin transition (red squares) or slightly detuned from the resonance (blue dots). The fitting of the oscillation shows an inhomogeneous dephasing time of ${T}_{2,{\rm{n}}}^{* }=16.6$ μs. **c**, The pulse sequence of nuclear spin Hahn echo. **d**, The nuclear spin Hahn echo measurement shows a slow decay with *T*_2,n_ = 162 μs. Source Data

We further characterize the nuclear spin coherence through Ramsey and Hahn echo sequences, as illustrated in Fig. 4a,b. In the Ramsey interferometry, when RF pulses are applied exactly in resonance with the nuclear spin transitions, we observe a slow decay in 20 μs. With a slight detuning of the RF frequency, a further oscillation is observed alongside the original decay. By fitting the oscillation using the equation $a\cos (\omega \tau +\phi ){{\rm{e}}}^{-\tau /{T}_{2,{\rm{n}}}^{* }}+c$, we determine the inhomogeneous dephasing time to be ${T}_{2}^{* }$ = 16.6 μs and the oscillation frequency to be the same as the detuning. Figure 4d shows the exponential decay measured by the nuclear spin Hahn echo sequence, revealing a nuclear spin coherence time of *T*_2,n_ = 162 μs. The nuclear spin coherence time is comparable with the electron spin relaxation time *T*_1,e_ = 144 μs (Supplementary Fig. 15), suggesting that the measurement is limited by the electronic spin lifetime.

## Identifying chemical structures

**Fig. 5:**
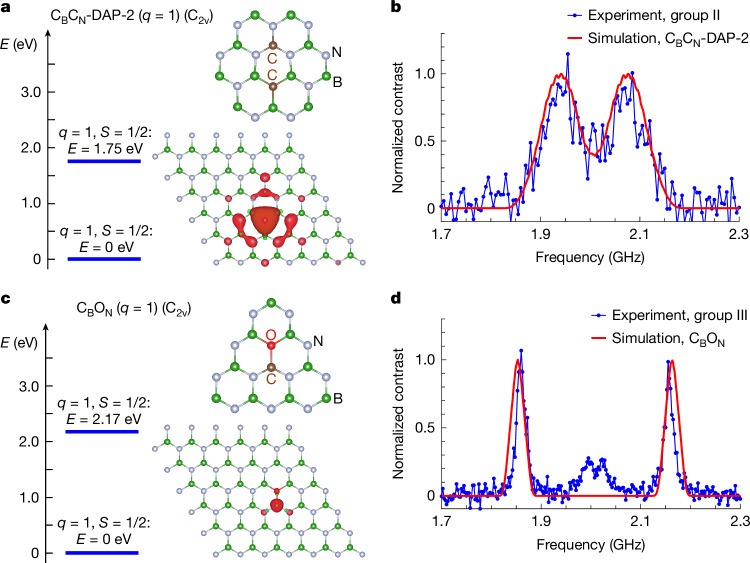
Candidates for the defect members in spin pairs. **a**, Chemical structure, spin density and energy-level diagram of the positively charged ${{\rm{C}}}_{{\rm{B}}}^{+}{{\rm{C}}}_{{\rm{N}}}^{0}\text{-DAP-2}$ defect. **b**, Simulated ODMR spectrum of the positively charged ${{\rm{C}}}_{{\rm{B}}}^{+}{{\rm{C}}}_{{\rm{N}}}^{0}\text{-DAP-}2$ defect based on the calculated hyperfine coupling parameters. The simulation result (red curve) is compared with the experimental result (blue curve). **c**, Chemical structure, electronic wavefunction and energy-level diagram of the positively charged C_B_O_N_ defect. **d**, Simulated ODMR spectrum of the positively charged C_B_O_N_ defect based on the calculated hyperfine coupling parameters. The simulation result (red curve) is compared with the experimental result (blue curve). Source Data

As well as providing long-lived spin qubits, nuclear spin magnetic resonance offers valuable insight into identifying defect structures. Here we perform first-principles calculations of the static properties and hyperfine interaction tensors to enable direct comparison with experimental data. Our calculations suggest that C_B_C_N_-DAP-*L* (ref. ^36^) is a likely member of the spin pair responsible for group II defects (Fig. 5a). This dimer can exist in two charge states: ${{\rm{C}}}_{{\rm{B}}}^{+}{{\rm{C}}}_{{\rm{N}}}^{0}\text{-DAP-}L$ and ${{\rm{C}}}_{{\rm{B}}}^{0}{{\rm{C}}}_{{\rm{N}}}^{0}\text{-DAP-}L$. The positively charged state (${{\rm{C}}}_{{\rm{B}}}^{+}{{\rm{C}}}_{{\rm{N}}}^{0}\text{-DAP-}L$) has an *S* = 1/2 manifold, and our calculations predict a ^13^C_N_ hyperfine coupling of 135 MHz (for *L* = 2). This value closely matches the experimentally observed value of 130 MHz. The simulated ODMR spectrum aligns well with the measured ODMR, as shown in Fig. 5b. Also, the calculated hyperfine coupling for the nearest ^11^B nuclear spins is 17.6 MHz, resulting in an ODNMR resonance at 8.8 MHz, which closely matches the observed ODNMR resonance at 9.2 MHz in Fig. 2h. Because the spin density is primarily localized at the C_N_ site and is barely affected by the distance *L*, the hyperfine coupling strength remains nearly uncharged for different *L* values (Supplementary Fig. 32).

To account for the observed 300-MHz hyperfine splitting in group III defects, we consider an oxygen substitution next to the ^13^C_B_ site, forming a C_B_O_N_ defect^10^ as one member of the spin pair. Oxygen impurities exist in hBN samples^37^ and can also be introduced during annealing owing to residual oxygen in the vacuum chamber. The positively charged state (${{\rm{C}}}_{{\rm{B}}}{{\rm{O}}}_{{\rm{N}}}^{+}$) of this defect hosts a spin-doublet manifold (*S* = 1/2) (Fig. 5c) with a hyperfine interaction strength *A*_*zz*_ = 314 MHz, in close agreement with our measured 300 MHz splitting. In Fig. 5d, the simulated ODMR linewidth induced by the nuclear spin bath is 23 MHz, comparable with the 16 MHz linewidth measured under weak microwave driving (Supplementary Fig. 13). The central peak (III-3) in Fig. 5d) is attributed to a second nearby spin defect.

For both group II and III defects, the structures discussed above correspond to the defect members in the spin pairs that exhibit strong hyperfine coupling. We expect that a second, nearby donor or acceptor defect couples to the proposed defect candidates to form a defect DAP complex. The structure of the other defect remains unclear owing to lack of characteristic hyperfine features. Further identification of both defect constituents may be possible by using high-purity hBN to suppress intrinsic ^12^C impurities, combined with higher ^13^C implantation density. This approach would improve the probability that both defects interact with ^13^C nuclear spins, enabling more definitive structural assignments.

## Conclusion

In conclusion, we report the detection and coherent control of single ^13^C nuclear spins using single hBN spin defects at room temperature. We find three distinct defect groups in ^13^CO_2_-implanted hBN samples, categorized on the basis of their ODMR spectra. We observe both *S* = 1/2 and *S* = 1 spin states within a single hBN spin defect complex, which shows quantum coherence at room temperature and ODMR contrast up to 200%. Also, the electronic spin state can be read out using a reasonably long laser pulse of around 5 μs, yielding approximately one photon per readout pulse (Supplementary Fig. 16) and a single-short spin readout efficiency of *η* = 0.12 (see Methods).

By making use of the control of individual resonances within the well-resolved hyperfine structures, we demonstrate initialization, coherent control and readout of a single ^13^C nuclear spin using spin defects in group II (Supplementary Information Section IV) and group III. The nuclear spins exhibit coherence times that are orders of magnitudes longer than those of electronic spins in hBN, offering the potential for long-lived quantum registers. The well-resolved hyperfine structure, combined with the high readout efficiency of spin states enabled by the high ODMR contrasts and the extended nuclear spin coherence times, makes this approach promising for achieving single-shot readout of individual nuclear spins^26^. This capability is crucial for implementing quantum error-correction protocols in a quantum register^27^. Moreover, the ^13^C nuclear spin can serve as a quantum memory to enhance quantum sensing with single hBN spin defects.

## Methods

### Sample preparation

The hBN thin flakes were tape-exfoliated from a monocrystalline hBN crystal and transferred onto Si/SiO_2_ substrates. Then we irradiated the hBN flakes with 2.5 keV ^13^CO_2_ (99.0% ^13^C, Sigma-Aldrich) ions with a dose density of 10^12^ cm^−2^ using a home-built ion implanter. The sample is then annealed at 1,000 °C at 10^−5^ Torr for 2 h to activate the carbon-related defects. For ODMR measurements, we transferred the hBN flakes to a coplanar waveguide using the standard dry transfer method with propylene carbonate stamps. The waveguide is made of 200-nm-thick silver with a 4-nm-thick Al_2_O_3_ layer on top.

### Sensitivity of a single spin defect

The ODMR contrast varies between defects and can reach as high as 200% (Supplementary Fig. 24). Among the more than 100 spin defects investigated, approximately 25% exhibit a contrast higher than 10%. A single hBN spin defect in our sample has a typical sensitivity of $5\,\mu {\rm{T}}/\sqrt{{\rm{Hz}}}$ for DC magnetic-field sensing, calculated using $(8{\rm{\pi }}/3\sqrt{3})(1/{\gamma }_{{\rm{e}}})(\Delta \nu /C\sqrt{I})$ (ref. ^29^), in which Δ*ν* is linewidth (20 MHz), *C* is the contrast (30%) and *I* is the photon count rate (170 kcts s^−1^). Furthermore, although most group II and III defects exhibit stable behaviours under a weak laser excitation (≤15 μW), the stability of group I defects varies greatly.

### Estimation of nuclear spin polarization

We estimate the polarization of the ^13^C nuclear spin by evaluating the imbalance between III-2 and III-4 in the ODMR spectrum. The ODMR is taken after the SWAP gate to transfer the electron polarization to the ^13^C nuclear spin. By using the fitted relative populations of the hyperfine basis states, the polarization can be calculated by the equation

2 $$P=\frac{{\sum }_{{m}_{I}}{m}_{I}{\rho }_{{m}_{I}}}{I{\sum }_{{m}_{I}}{\rho }_{{m}_{I}}}=\frac{{\rho }_{1/2}-{\rho }_{-1/2}}{{\rho }_{1/2}+{\rho }_{-1/2}}.$$

### Spin readout efficiency

The efficiency of a single-shot spin readout is an important factor to estimate how efficiently we can determine the electronic spin state of a spin defect, which is highly dependent on the defect properties. The readout efficiency is defined by the signal-to-noise ratio from a single readout pulse and can be expressed as^38^

3 $${\eta }_{{\rm{s}}}=1/{\sigma }_{{\rm{s}}}={\left(1+2\frac{{\alpha }_{0}+{\alpha }_{1}}{{({\alpha }_{0}-{\alpha }_{1})}^{2}}\right)}^{-1/2},$$

in which *α*_0_ and *α*_1_ are the mean numbers of detected photons for a single measurement of the brighter state and darker state, respectively. We estimate the efficiency based on the pulsed ODMR measurements. The pulsed ODMR contrast is 17.5% when we set the readout duration at 5 μs under a *P*_MW_ = 60 mW microwave drive. The contrast reaches 28% when *P*_MW_ = 2 W. For each readout laser pulse, we obtain approximately 0.9 photons from the darker state under the 15-μW laser pumping. These yield the efficiency of 0.08 and 0.12 for *P*_MW_ = 60 mW and *P*_MW_ = 2 W, respectively. See details in Supplementary Information Section IV.

### Gate fidelity

For Rabi oscillations limited by a pure dephasing process, we can write the π-gate fidelity as *F*_π_ = 0.5(1 + exp(−1/*Q*_π_)), in which *Q*_π_ = *T*_Rabi_/*T*_π_ is the quality factor of a π gate^39^. We extract the coherence time and π-gate time of nuclear spin Rabi by fitting the results in Fig. 3 to the function *C*(*τ*) = *a*sin(π*τ*/*T*_π_ + *b*)exp(−*τ*/*T*_Rabi_) + *d*, in which *C*(*τ*) is the signal contrast of Rabi. As a result, we obtain a *F*_π,n_ = 99.75% π-gate fidelity, with *T*_π,n_ = 0.60 μs and *T*_Rabi,n_ = 117 μs. Similarly, we also estimate the electronic spin π-gate fidelity to be 96.2%, using the same defect and transition in the nuclear spin control experiments.

### DFT calculations

We use Quantum Espresso^40^, an open-source plane-wave software, to perform the DFT calculations. Both the Perdew–Burke–Ernzerhof functional and the Heyd–Scuseria–Ernzerhof hybrid functional (the factor of 0.32 for Fock exchange)^41,42^ are used for the exchange-correlation interaction. We use the optimized norm-conserving Vanderbilt (ONCV) pseudopotential^43,44^ for the calculations of excitation energy and the GIPAW pseudopotential^45^ for the calculation of hyperfine interaction parameters and ZFS. We set the kinetic energy cut-off to be 55 Ry, which is adequate for converging the relevant properties. Geometry optimizations are carried out with a force threshold of 0.001 Ry Bohr^−1^. We select the 6 × 6 × 1 or higher supercell size of hBN for the calculations of hyperfine parameters and excitation energies. For these calculations, we sample a *k*-point mesh of 3 × 3 × 1 for the calculation of excitation energies^46^ and the *Γ* point for the hyperfine parameters and ZFS^28,46^. We calculate the zero-phonon line by the constraint occupation DFT method^47^, the hyperfine parameters using the QE-GIPAW code^48^, the ZFS by using the ZFS code^46^ and we cross-compare results between the ZFS code and the PyZFS code^49^. The key results are summarized in Supplementary Tables 2 and 3.

### Simulation of ODMR spectrum

The continuous-wave ODMR spectra shown in Fig. 5 are simulated using the MATLAB toolbox EasySpin^50^ based on data from the ab initio calculations. EasySpin also takes the nuclear Zeeman and quadrupole interaction into account. Therefore, the continuous-wave ODMR linewidth can be determined according to the hyperfine couplings with the most abundant nuclear-spin-active isotopes: ^13^C, ^11^B and ^14^N. In our simulation, we consider a ^13^C nuclear spin, ten nearest ^11^B nuclear spins and two proximate ^14^N nuclear spins. The other nuclei, located further away, couple more weakly to the electron, scaling with  ∝ 1/*r*^3^ (in which *r* is the distance from the central carbon site) and, thereby, have a negligible effect on the ODMR linewidth.

### Spin-pair model

The *S* = 1 transitions are consistently observed alongside the *S* = 1/2 transitions within the same emitters. To explain the coexistence of both spin transitions in ODMR, we use a weakly coupled spin-pair model^32,33^ and perform numerical simulations to investigate the underlying mechanism.

**Extended Data Fig. 1:**
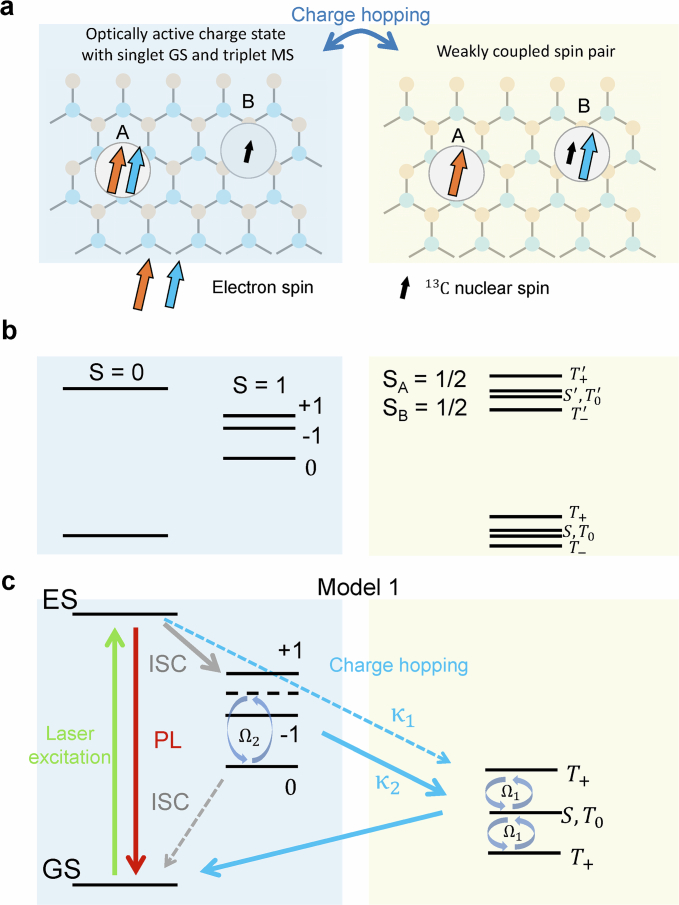
Spin-pair model. **a**, Two possible electron configurations of a spin defect complex in different internal charge states. **b**, The energy levels of internal charge states according to the DFT prediction. When two electrons occupy the same defect site (left), it forms a singlet GS and ES, as well as a triplet metastable state. When two electrons locate at different defect sites (right), each form a *S* = 1/2 state and they are weakly coupled through dipolar interaction, forming a spin *S* = 1/2 pair. **c**, A proposed energy-level diagram for explaining the coexistence of the *S* = 1/2 and *S* = 1 ODMR transitions.

Extended Data Fig. 1a provides a simplified representation of the spin-pair model, consisting of two independent defects (defects A and B), separated by ≥1 nm, forming a defect complex. This complex hosts two unpaired electrons that establish different internal charge states depending on their spatial occupancy. When both electrons are localized on the same defect (defect A), they form a closed-shell spin singlet GS with a metastable spin triplet state (*S* = 1), which can be accessed through laser excitation and intersystem crossing transitions (left panel in Extended Data Fig. 1b). This state corresponds to a strongly coupled spin-pair charge state and explains the *S* = 1 transitions.

Alternatively, laser excitation can induce charge hopping that transfers one electron from defect A to defect B, forming a weakly coupled defect pair. In this configuration, each defect hosts a single electron (*S* = 1/2), as illustrated in the right panel of Extended Data Fig. 1b. This spin-dependent charge hopping yields a corresponding spin-dependent PL signal^32^.

The actual GS, which is also the optically active state, is determined by the lowest-energy charge configuration and depends on the specific defect species and the local Fermi energy level. We considered two possible energy-level configurations (Supplementary Information Section XII), with Extended Data Fig. 1c depicting the most likely scenario. In the most stable charge state, both electrons occupy the same defect site, yielding a *S* = 0 GS and a metastable *S* = 1 state. Laser excitation can then generate a metastable spin-pair charge state by promoting transitions from the *S* = 1 to the *S* = 1/2 manifold. The pronounced asymmetry in the Rabi oscillations, reflected by an increasing contrast baseline in both *S* = 1/2 and *S* = 1 transitions, strongly supports the metastable nature of these spin manifolds.

In the presence of a ^13^C nuclear spin, the defect electron spins can couple to the nuclear spin through hyperfine interactions, which vary across different charge states. In the weakly coupled spin-pair state, the nuclear spin is primarily coupled to a single electron spin at defect A. This is consistent with our experimental observations, in which hyperfine coupling constants *A*_*zz*_ of 130 and 300 MHz were measured for group II and group III defects, respectively.

Given their distinct hyperfine features, group II and III defects are likely to have more well-defined and deterministic structures. By contrast, group I defects, lacking hyperfine splitting, may include a range of chemical configurations: either similar to group II and III defects (but involving ^12^C instead of ^13^C) or very different chemical structures. This structural variability may account for the broader range of stability observed in group I defects, whereas group II and III defects tend to exhibit more consistent and stable behaviour under experimental conditions.

### Simulation of spin photodynamics

On the basis of the possible energy-level models, we numerically simulate the spin photodynamics using the Lindblad master equation:

4 $$\dot{\rho }=-\,i[H,\rho (t)]+\sum _{k}{\varGamma }_{k}\left[{L}_{k}\rho (t){L}_{k}^{\dagger }-\frac{1}{2}\{{L}_{k}^{\dagger }{L}_{k},\rho (t)\}\right],$$

in which *ρ*(*t*) is the time-dependent density matrix, *Γ*_*k*_ represents transition rates and *L*_*k*_ are the associated Lindblad operators. This simulation allows us to predict PL signals in both continuous-wave ODMR and pulsed Rabi experiments (see details in Supplementary Information).

In the continuous-wave ODMR simulation, we use the full Hamiltonian, which includes the optical manifold, spin-pair states and spin-triplet states. For model 1, in which the GS is a spin singlet (*S* = 0), the Hamiltonian is written as:

5 $$H={H}_{{\rm{pair}}}\oplus {H}_{m,S1}\oplus {H}_{{\rm{eg}}}=\left(\begin{array}{ccc}{H}_{{\rm{pair}}}^{8\times 8} &  & \\  & {H}_{m,S1}^{6\times 6} & \\  &  & {H}_{{\rm{eg}}}^{4\times 4}\end{array}\right)$$

in which *H*_eg_, *H*_*m*,*S*1_ and *H*_pair_ describe the optical manifold, the spin *S* = 1 metastable state and the spin-pair state, respectively (see detailed expressions in Supplementary Information). For each subspace, we consider a ^13^C nuclear spin coupled through hyperfine interaction. The full Hamiltonian is a direct sum of individual spin manifolds, meaning that there is no coherent interaction between them. Instead, they are connected by means of incoherent transitions described by the Lindblad operators *L*_*k*_.

## Online content

Any methods, additional references, Nature Portfolio reporting summaries, source data, extended data, supplementary information, acknowledgements, peer review information; details of author contributions and competing interests; and statements of data and code availability are available at 10.1038/s41586-025-09258-7.

## Supplementary information

Supplementary Information Supplementary Information, including Supplementary Figs. 1–42, Supplementary Tables 1–10 and Supplementary References.

## Source data

Source Data Fig. 1

Source Data Fig. 2

Source Data Fig. 3

Source Data Fig. 4

Source Data Fig. 5

## Data Availability

Source data for the figures in the main text are provided with this paper. Other data that support the plots in this paper and other findings of this study are available from the corresponding author on request.

## Code availability

The custom codes that support the findings of this study are available from the corresponding author on request.
