# Supplementary material for: Single nuclear spin detection and control in a van der Waals material
Source: Nature. 2025 Jul 9;643(8073):943–9. doi: 10.1038/s41586-025-09258-7 (PMC12286849; doi:10.1038/s41586-025-09258-7)
Supplement: Supplementary file 1 — Supplementary Information, including Supplementary Figs. 1–42, Supplementary Tables 1–10 and Supplementary References. [file 41586_2025_9258_MOESM1_ESM.pdf]

nature portfolio

https://doi.org/10.1038/s41586-025-09258-7

Supplementary information

Single nuclear spin detection and control in a van der Waals material

In the format provided by the authors and unedited

Supplementary Information for "Single nuclear spin detection and control in a van der Waals material"

Xingyu Gao,[1,*] Sumukh Vaidya,[1,*] Kejun Li,[2,3] Zhun Ge,[1] Saakshi Dikshit,[4] Shimin Zhang,[3] Peng Ju,[1] Kunhong Shen,[1] Yuanbin Jin,[1] Yuan Ping,[3,5,6] and Tongcang Li[1,4,7,8,†]

[1]*Department of Physics and Astronomy, Purdue University, West Lafayette, Indiana 47907, USA*
[2]*Department of Physics, University of California, Santa Cruz, CA, 95064, USA*
[3]*Department of Materials Science and Engineering, University of Wisconsin-Madison, 53706, USA*
[4]*Elmore Family School of Electrical and Computer Engineering, Purdue University, West Lafayette, Indiana 47907, USA*
[5]*Department of Physics, University of Wisconsin-Madison, 53706, USA*
[6]*Department of Chemistry, University of Wisconsin-Madison, 53706, USA*
[7]*Purdue Quantum Science and Engineering Institute, Purdue University, West Lafayette, Indiana 47907, USA*
[8]*Birck Nanotechnology Center, Purdue University, West Lafayette, Indiana 47907, USA*

(Dated: June 21, 2025)

Contents

I. Experimental setup 2

II. Sample preparation 3

III. Spin defects in $^{13}CO_2$ doped hBN 3

IV. Spin defects in Group II 5
A. Ground state ODMR spectroscopy 5
B. Electron spin manipulation and verification of spin multiplicity 6
C. Optically detected NMR and coherent control of ^{13}C nuclear spin 8
D. Other examples of Group II spin defects 10

V. Spin defects in Group III 12
A. ODMR spectroscopy 12
B. Spin coherence and readout efficiency 14
C. Optically detected nuclear spin magnetic resonance 16
D. Coherent control of ^{13}C nuclear spin using Defect 10 18
E. Other examples of Group III spin defects 18

VI. Spin defects in Group I 20
A. GS ODMR spectroscopy and spin coherence properties 20
B. Other examples of Group I defects 21
C. Variation in Zero-Field Splitting (D) of S = 1 Transitions 22

VII. Summary of defect examples 23

VIII. Spin defects in $^{12}CO_2$ implanted hBN 24

IX. Spin defects in Helium ion implanted hBN 25

X. Non-implanted hBN with thermal annealing 26

XI. DFT calculations of possible defect candidates 26

---

* These authors contributed equally to this work.
† tcli@purdue.edu

A. Hyperfine Interaction 27
B. Zero-Field Splitting 27
C. Results and discussion 28

XII. Modeling spin dynamics: spin pair, charge state conversion and energy levels 35
A. Asymmetry and Beating in Rabi oscillation 37
B. Modeling B-field dependent ODMR spectra 41
1. Model 1: Singlet GS, metastable S = 1 states, and metastable spin pair 41
2. Model 2: GS spin pair and metastable triplet states 43

References 46

I. Experimental setup

All the measurements were carried out at room temperature using a home-built confocal microscope system. We use a continuous-wave 532-nm laser to initialize and detect hBN defect spins. The laser is shuttered by an acousto-optic modulator (ISOMET, M1205-T110L-1) in a double-pass configuration to achieve $> 10^5$:1 on/off ratio. An objective lens (NA=0.9) is used to focus the laser beam and collect the fluorescence from the samples. The collected fluorescence is separated from the laser beam by a dichroic mirror, filtered through a long-pass filter, coupled into a multi-mode fiber and finally detected by a single photon counting module (Excelitas, SPCM-AQRH). The sample is mounted on a 3D translational stage to control the position. The lateral scanning is enabled by an galvanometer scanner (Thorlabs GVS212).

To control the electron spin, we use two microwave sources (Stanford Research Systems SG386) to generate microwaves and select one of the microwave sources as the input by using an RF switch (Mini-Circuits ZASWA-2-50DRA+). For most ODMR and ODNMR measurements, only one microwave source is used. The second microwave source is used for estimating nuclear spin polarization, where two distinct microwave frequencies are needed in the same pulse sequence. The amplitude of the microwave field is modulated by another RF switch (Mini-Circuits ZASWA-2-50DRA+) and then amplified by an amplifier (Mini-circuits ZHL-10W-202s or ZHL-16-43-s+).

For controlling the nuclear spins, we add an additional RF driving field using a signal generator (Teledyne LeCroy, T3AFG200). Similarly, the RF field is switched by an RF switch (Mini-Circuits ZASWA-2-50DRA+) and then amplified by an amplifier (LZY-22+). The microwave and RF fields are combined using an RF splitter (ZFRSC-42-S+). Prior to this, we add the long pass filter (Mini-Circuits, VHF-1080+) at the microwave input and low pass filter (VLF-490+) at the RF input to prevent signal interference and ensure proper signal routing. The instruments are gated through a multi-channel pulse streamer (Swabian Instruments Pulse Streamer 8/2) with 1 ns temporal resolution.

In our experiments, an external magnetic field is applied along the out-of-plane (z-axis) direction using a permanent magnet mounted on a 3D translational stage. The magnetic field is aligned and calibrated using boron vacancy (V_B^-) defects in hBN, which have a well-characterized g-factor of 2. The non-resonant PL signal of boron vacancy defects exhibits a characteristic response to changes in the magnetic field magnitude and orientation [1, 2]. We leveraged this feature to calibrate and align our magnetic field along the z axis.

The calibration procedure consists of the following steps:

1. We align the magnet to the laser beam (without the objective lens) to ensure that the laser hits the center of the magnet. The reflected beam is adjusted to overlap with the incoming beam, providing an initial rough alignment.

2. After reinstalling the objective lens, we mount the hBN sample containing boron vacancy defects. The x and y positions of the magnet stage are fixed, and the magnetic field is adjusted along the z-axis to locate the level anti-crossing (LAC) position.

3. At the LAC, we scan the (x, y) positions and plot a 2D PL map to identify the local maximum signal. The magnet is then finely tuned to this optimal (x, y) position. This method ensures an alignment uncertainty of the magnetic field orientation of less than 0.5°.

In the experiments, we use different microwave powers depending on the specific measurement being performed. For ODMR contrast mapping, we typically apply a low microwave power (∼30 mW) to minimize power broadening and better resolve defects with distinct hyperfine features. At this power level, the thermal drift of the sample is small—typically less than 500 nm per day for continuous measurements. After acquiring a scan, we locate the target defect based on the ODMR contrast map and then perform a fine-positioning scan around the region of interest. This fine scan takes only a few seconds, during which drift can be neglected.

The strong microwave drive (up to 1 W) is used only during pulsed ODMR experiments such as Rabi oscillations, T_1 and T_2 measurements for fast coherent control. This power level refers to the microwave power during the pulse, which

typically lasts tens to hundreds of nanoseconds. For most of the sequence duration, including the laser initialization (∼10 μs or more) and readout (∼10 μs or more), the microwave is off. As a result, the average microwave power applied to the sample remains low (typically <100 mW, often less than 10 mW), minimizing sample heating and drifting. In nuclear spin resonance experiments, the RF pulses are stronger and longer, and thermal drift is more noticeable. However, it stabilizes over time. To actively compensate for any residual drift, we implemented a peak-finding routine that re-optimizes the laser focus on the defect after each scan or when position shifts are detected. This feedback control allows us to maintain precise alignment with the target defect throughout extended measurements.

II. Sample preparation

A monocrystalline hBN (from 2D Semiconductors) was tape exfoliated into thin flakes, which were transferred onto Si substrate (with a 285 nm SiO_2 layer). The hBN flakes were irradiated with 2.5 keV ionized $^{13}CO_2$ (99.0% ^{13}C, Sigma-Aldrich) using a home-built ion implanter with a dose density of 10^{12} cm^{-2}. The sample is then annealed at 1000 °C at 10^{-5} torr for 2 hours. After annealing, the hBN flakes were transferred to a coplanar waveguide. The waveguide is made of 200 nm thick silver with a 5 nm thick Al_2O_3 layer on top which helps stabilize the charge state of spin defects. The hBN contains a large number of unstable emitters just after annealing. We further treat the flake by a 2D confocal scan with strong laser exposure (∼500 μW) for 100 ms at each pixel (step size 50 nm).

Our sample preparation method yields approximately 20-30% ODMR-active defects. Among these, around 10% belong to Group II and Group III, while the remaining are primarily Group I defects, along with a small fraction of unidentified spin defects. Additionally, the thickness of the hBN flake also affects defect yield efficiency and stability. The optimal thickness for achieving a high yield of single ODMR-active emitters is typically 10–30 nm (Figure S1). In thicker hBN flakes, we generally observe higher PL background, reducing single-photon purity. Conversely, if the hBN is too thin, the defect's proximity to the metallic waveguide substrate can impact charge stability, leading to more pronounced blinking effects, particularly when the thickness is below 10 nm. Introducing a protective layer between the hBN and the waveguide could further improve defect stability.

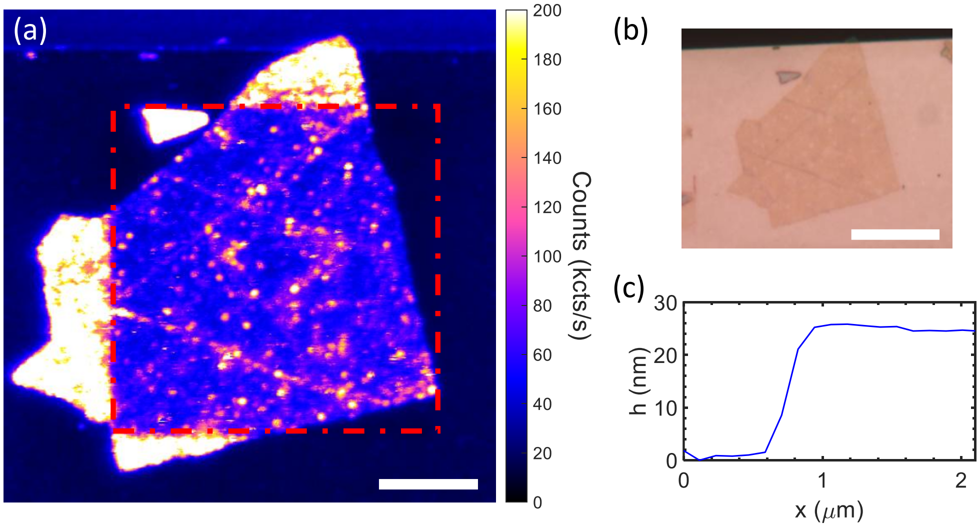

Figure S1. (a) Confocal image of a hBN flake with single spin defects taken with 20 μW laser excitation. The region enclosed by the red dashed line has been performed by a 2D confocal scan with 600 μW laser exposure. The region outside has not been treated by strong laser exposure. Scale bar: 10 μm. (b) Optical image of the flake. Scale bar: 20 μm. (c) A profile measured by AFM showing a flake thickness of 25 nm.

III. Spin defects in $^{13}CO_2$ doped hBN

After ion implantation and thermal annealing, we observe a large density of bright emitters within the flake, but these emitters are nearly continuously distributed and most of them are unstable under laser excitation. To obtain stable single spin defects, we perform a 2D confocal scan with a relatively strong laser exposure (∼500 μW) over

the flake. The strong laser exposure quenches most unstable defects, after which the number of emitters significantly decreases and isolated spots can be resolved (Figure S1).

Next, we perform continuous wave (CW) ODMR measurements to identify the spin resonance spectroscopy. An external magnetic field is applied along the c-axis of hBN (out-of-plane). The Hamiltonian of the spin system in this condition can be written as

$$H = DS_z^2 + E(S_x^2 - S_y^2) + \gamma_e \mathbf{B} \cdot \mathbf{S} + \sum_i \mathbf{S} \cdot A_i \cdot \mathbf{I_i} + \gamma_n \mathbf{B} \cdot \mathbf{I_i}, \tag{1}$$

where $\mathbf{S}$ is the spin operator, γ_e is the electron spin gyromagnetic ratio, $\mathbf{B}$ is the external magnetic field, γ_n is the nuclear spin gyromagnetic ratio. D and E are the longitudinal and transverse zero field splitting (ZFS) parameters. The hyperfine interaction between the electron and surrounding nuclear spins $\mathbf{I_i}$ are characterized by the hyperfine interaction strength tensor A_i.

Among the isolated spots, several different types of ODMR spectra are observed. Figure 1 in the main text shows three main types of ODMR spectra measured at room temperature. In all the groups, we see a center resonance at the frequency proportional to the external magnetic field, and we also observe two side resonance approximately 1 GHz away from the center. Within the center branch, there can be different numbers of peaks, and we assign them into Group I to III by the peak number from 1 to 3, respectively. The splitting among these center peaks is attributed to the hyperfine interaction with the proximate ^{13}C nuclei. Here, we also note that some defects show more complicated spectra in the ODMR measurements. But since they are rarely observed and hardly reproducible, we assign them into Group Others. The defects in Group I do not show hyperfine splitting, indicating a weak coupling with ^{13}C nuclear spins or the presence of ^{12}C instead of ^{13}C.

Figure 1(g) in the main text illustrates an example ODMR spectrum of a Group I defect, wherein only one resonance, I-2, is discernible at the center branch. Despite employing weak microwave driving to minimize the linewidths to as small as 20.9 MHz (I-2) and 10.8 MHz (I-1, I-3) (Figure S22-S25 in Supplementary Information), hyperfine structure is unresolved for any of the three resonances (I-1 to I-3). It is worth noting that the minor peak to the left of I-1 does not represent an additional resonance but rather originates from the resonance I-2, driven by the second harmonic leaked from the microwave amplifier.

Figure 1(h) shows an ODMR spectrum of a representative Group II defect, featuring a two-peak hyperfine structure (II-2 and II-3) at the center. The 130 MHz separation between II-2 and II-3 suggests the presence of a ^{13}C nuclear spin strongly coupled to the defect electron spin. In contrast, three side peaks (II-1, II-4, and II-5) exhibit no discernible hyperfine structure. This observation implies that the center branch and the additional resonances stem from different spin systems with distinct hyperfine coupling strengths with the ^{13}C nuclear spins. Here,we also note that the II-5 transition is not always observed in every defect. This peak is absent or falls below our $\sim$0.5 % contrast noise floor in many defect.

The ODMR spectrum of Group III defects contains three peaks (III-2, III-3, and III-4) within the center branch, as depicted in Figure 1(i). Individual ODMR contrasts within the center branch can reach as high as 50% (Figure S13 in Supplementary Information). With weak microwave driving, an additional splitting is observable at peak III-3, showing a separation of 34 MHz. We anticipate that resonance III-3 corresponds to a different spin separate from the spin associated with resonances III-2 and III-4. Specifically, the electron spin contributing to III-2 and III-4 interacts with a single ^{13}C nuclear spin with a coupling strength of 300 MHz. Conversely, the electron spin involved in III-3 exhibits weaker coupling to a ^{13}C nuclear spin, with a strength of 32 MHz. Further supporting evidence will be provided in subsequent discussions.

Figure S2(a) shows confocal map a ^{13}C doped hBN sample after just strong laser exposure. We further map out the ODMR contrast spatial distribution by driving the microwave at three different frequencies (S2(b)-(d)). 2.01 GHz is around the center frequency at 71.8 mT where we see many high contrast spots in the flake. By driving the microwave at II-2 resonance of Group II and III-2 resonance of Group III, the numbers of high contrast spots are much fewer. Group I defects are the most commonly observed defects in a new sample just after strong laser exposure. But more than half of the Group I defects show blinking or quenching during the ODMR experiments, which may come from charge dynamics. Most spin defects that shows significant blinking will finally become spin inactive during the ODMR measurements (usually in a few minutes). For the defects without obvious blinking effect, they mostly remain stable for more than several weeks of measurements. Despite relatively low occurrence of Group II and III defects compared to the Group I defects, most of these defects (Group II and III) exhibit good stability under a weak laser excitation ($\sim$15 μW).

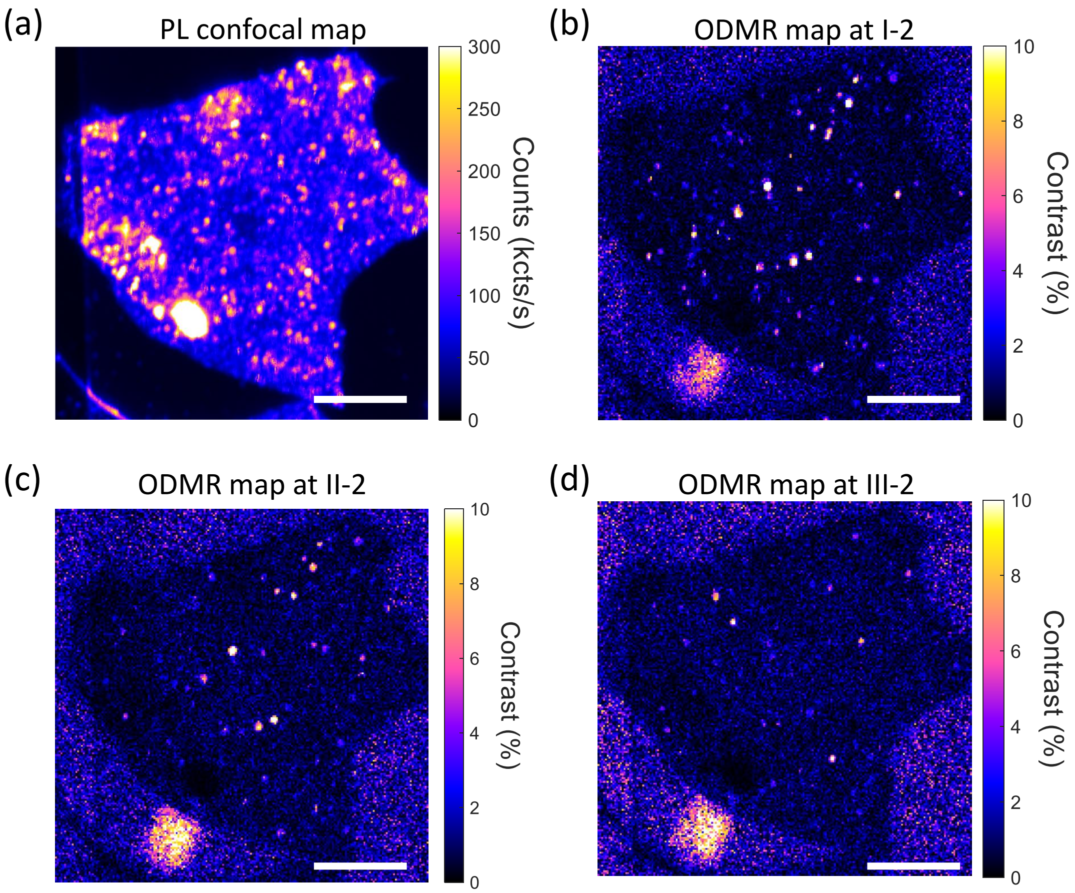

Figure S2. (a) Confocal PL map of a $^{13}CO_2$ implanted hBN after strong laser exposure. (b)-(d) ODMR contrast map by driving the microwave at (b) I-2, (c) II-2, and (d) III-2. An external magnetic field of 71.8 mT is applied along the out-of-plane direction. The microwave power was set to 30 mW to minimize linewidth broadening due to power effects and reduce sample drift, while maintaining a reasonable ODMR contrast. Contrast fluctuations outside the hBN flake arise from low photon counts in the background. Scale bars are 10 μm.

IV. Spin defects in Group II

A. Ground state ODMR spectroscopy

Figure S3 (a) displays the evolution of the ODMR spectra of Defect 2 (Table S1 in Section VII) in different out-of-plane magnetic fields. The spectrum reveals five distinct resonances, labeled II-1 to II-5, at frequencies ν_i (i =1-5). Resonances II-1 to II-4 exhibit a g-factor of g = 2, confirming their electronic spin nature. Resonance frequencies of II-2 and II-3 scale linearly with the external magnetic field without ZFS. Fitting the resonance II-1 and II-4, we extract ZFS parameters of D = 1.1 GHz and E = 0.4 GHz, suggesting a S$\geq$1 state with an out-of-plane intrinsic quantization axis.

The resonance frequency of II-5 shows a steeper slope (an effective g-factor of 4) as a function of the external magnetic field. To gain further insight into this transition, we performed a finer scan over the energy-level crossing between II-4 and II-5. Notably, a LAC is observed near 37 mT (Figure S3(b)). Additionally, the frequency ν_5 of R5 turns out to be $\nu_4 + \nu_1$ ($\nu_4 - \nu_1$), when B > 37 mT (B < 37 mT). These findings suggest that II-5 originates from the same spin system as II-4 and may correspond to a double-quantum transition.

Next, we examine the pulsed ODMR spectrum of this defect (Figure S3(d)). The experimental sequence includes a 10 μs laser initialization pulse, a 5 μs readout laser pulse, a 5 μs wait time between them, during which a 30 ns microwave pulse is applied. The pulsed ODMR spectrum reveals the same resonance peaks as the cw-ODMR spectrum, indicating that the corresponding spin states reside either in the ground state (GS) or in a long-lived metastable state with a lifetime exceeding 5 μs.

To further understand the ODMR spectrum, we conduct CW ODMR measurements at different laser excitation

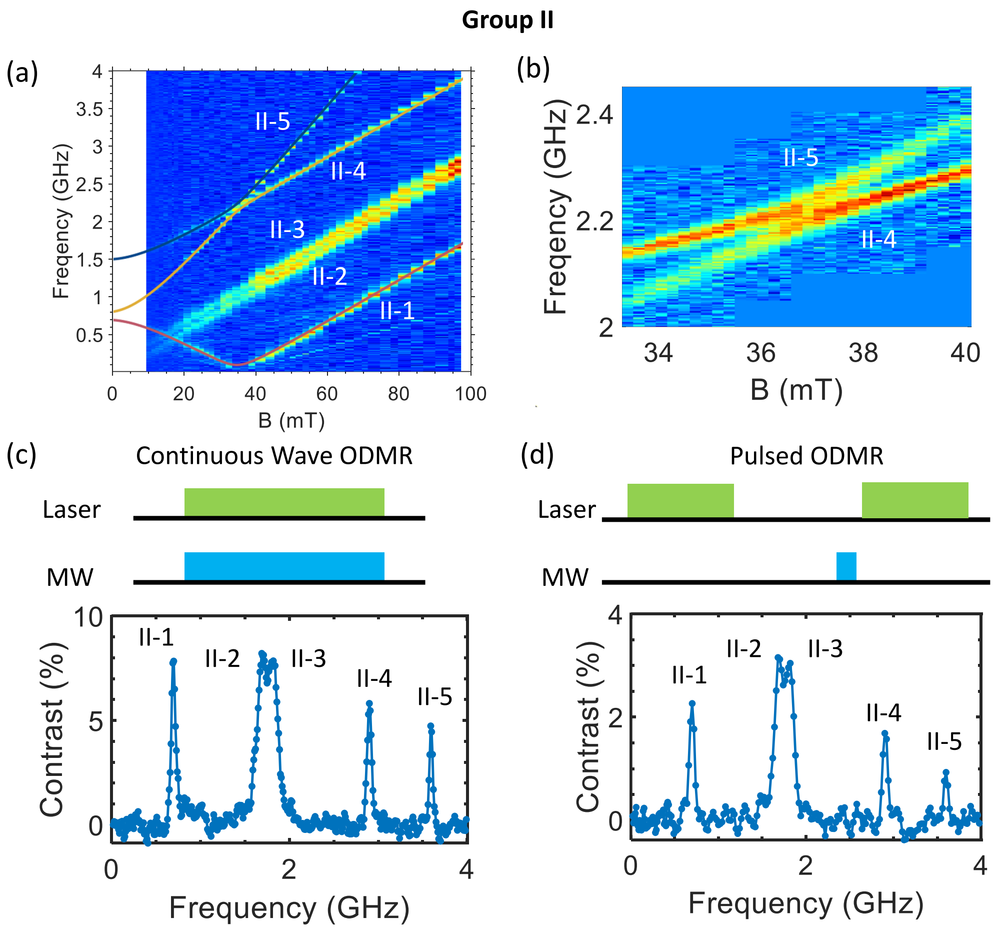

Figure S3. **ODMR of Defect 2 in Group II.** (a) cw-ODMR spectrum as a function of magnetic field. (b) A fine scan of cw-ODMR spectrum near 37 mT. (c) A cw-ODMR spectrum measured at 62.5 mT. (d) Pulsed ODMR of Defect 2 measured at 62.5 mT.

powers as shown in Figure S4. The steady-state ODMR contrasts of resonance II-1 and II-2 are plotted as a function of the incident laser power. Both contrasts initially increase, then peak at a laser power of 20 μW, and finally start to drop when the laser intensity is ramped up further. Meanwhile, the PL count increases when the laser excitation power increases (Fig. S5).

In addition, we also determine the saturation contrast and unsaturated linewidth of the ODMR spectra by performing the microwave power dependent measurements. As shown in Figure S6, under a weak microwave driving, the unsaturated ODMR of II-1 has a narrow linewidth of around 16 MHz at 71.5 mT. No visible hyperfine splitting is observe in II-1, indicating that the upper limit of hyperfine coupling to the ^{13}C nuclei is less than 16 MHz. By applying a strong microwave ($>$ 100 mW), II-1 saturates in contrast and displays a large distorting in the lineshape. In contrast, II-2 does not show saturation behavious in ODMR with an increasing microwave power in our experimental range. Instead, the contrast shows a rapid increase when we further ramp up the microwave power beyond 100 mW.

B. Electron spin manipulation and verification of spin multiplicity

All defects in hBN have symmetry not higher than D_{3h}, and most complexes have symmetry not higher than C_{2v} symmetry. Since the electronic states of a system with C_{2v} or lower symmetry are nondegenerate, it is more energetically favorable for electrons to pair up in one level than fill in the next unoccupied electronic level, unless the level splitting energy is smaller than the paring energy. Consequently, for the defects with C_{2v} or lower symmetry, a singlet (S=0) GS is more favorable if they have even number of electrons, and doublet (S=1/2) GSs are more common when the defects have odd number of electrons. It can be even more difficult in terms of energy to form a quartet (S=3/2) defect. So far, we haven't found one defect whose GS can be in high spin (S$\geq$3/2). As S=3/2 state

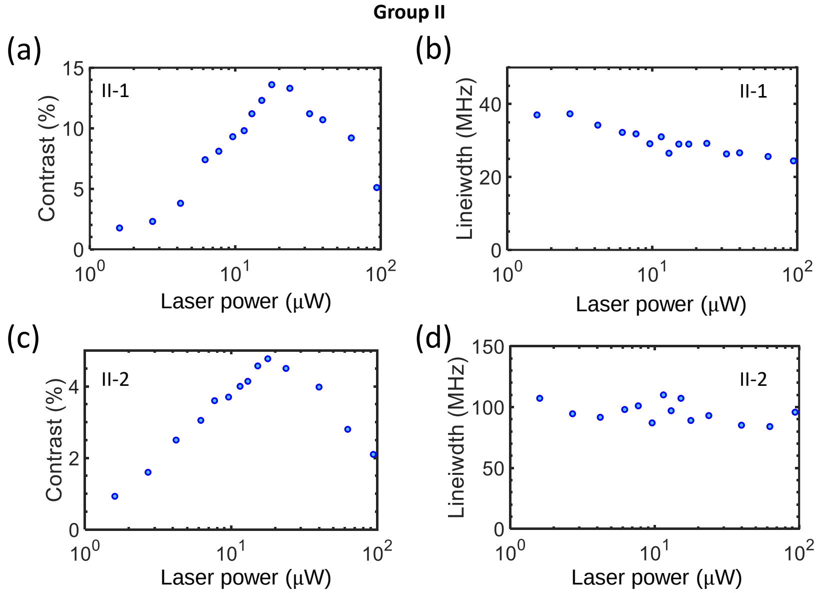

Figure S4. cw-ODMR measurements at different excitation laser power (Defect 6). (a) cw-ODMR contrast and (b) linewidth of II-1 as a function of the incident laser power. (c) cw-ODMR contrast and (d) linewidth of II-2 as a function of the incident laser power. Measurements are performed at 71.8 mT.

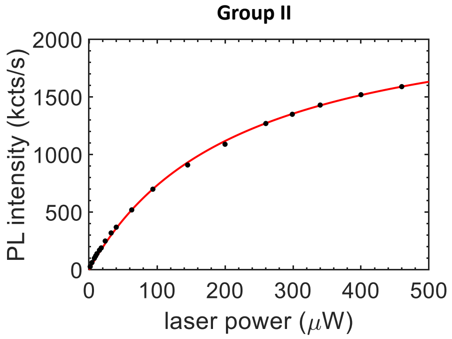

Figure S5. PL count rate as a function of the laser power (Defect 6).

is generally energetically unfavorable and consequently of low possibility, we don't consider S=3/2 spin state as the picture for explaining our experimental optical observation.

To explain the multiple transitions in the ODMR, we propose two spin systems with S=1/2 and S=1 coexist in the same defect that may come from different charge states. Resonances II-2 and II-3 are attributed to a spin-1/2 system that strongly couples to a single ^{13}C nuclear spin. Meanwhile, II-1, II-4 and II-5 originate from a spin-1 system that weakly couples to the surrounding nuclei. Such coexistence of spin-1 and spin-1/2 systems in a single defect has been recently discovered in hBN. The previous observation showed an in-plane intrinsic quantization axis [3], different from the out-of-plane spin axis that we observed.

To verify our argument on the spin multiplicity, we further perform the spin coherent control on these transitions and compare their Rabi frequencies. II-1 to II-4 shows clear Rabi oscillations when we vary the duration of a resonant microwave pulse (Figure S7). Driving in resonance with II-2, we observe a Rabi oscillation with a decay time of $T_{Rabi,II-2}$ = 97 ns (Figure S7(b)). The Rabi oscillation of II-1 persists for $T_{Rabi,II-1} = 0.75$ μs at a Rabi frequency of 46 MHz, resulting in a quality factor of π pulse Q = $T_{Rabi}/T_{\pi} = 69$ (Figure S7(c)). Resonance II-1 requires two closely spaced Rabi frequency components ($f_{rabi,1} = 44.56$ MHz, $f_{rabi,2} = 43.07$ MHz) to fit the oscillation data, which hints a possible splitting due to the weak hyperfine coupling with a ^{13}C nucleus.

The spin multiplicity can be obtained by comparing the Rabi frequencies at different resonances. The Rabi frequency is determined by the microwave driving Hamiltonian

$$H_d = \gamma_e B_{mw} S_x cos(\omega_{mw} t), \tag{2}$$

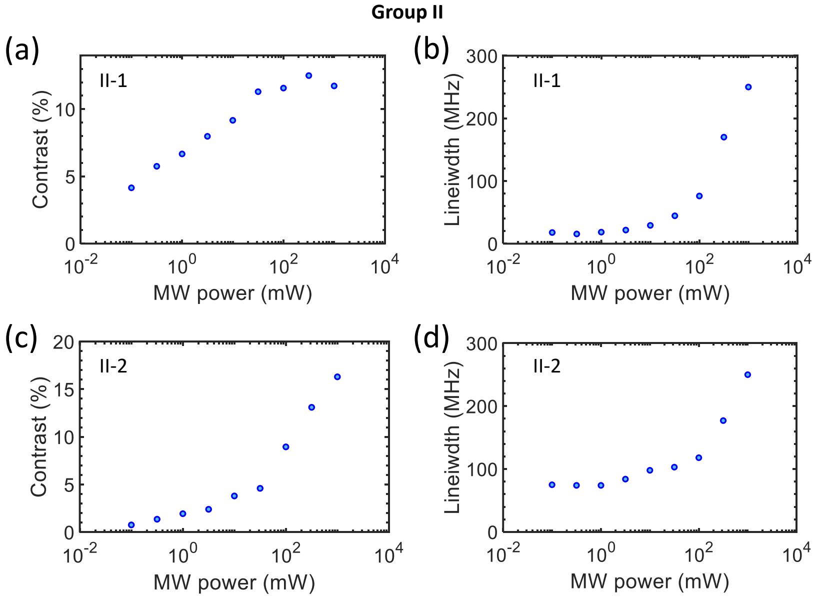

Figure S6. cw-ODMR measurements at different excitation microwave power (Defect 6). (a) cw-ODMR contrast and (b) linewidth of II-1 as a function of the microwave power. (c) cw-ODMR contrast and (d) linewidth of II-2 as a function of the microwave power. Measurements are performed at 71.8 mT.

where S_i $(i = x, y, z)$ is the GS spin operator. When the microwave frequency is in resonance with the spin transition frequency, the Rabi frequency is determined by the microwave field strength and the spin states. Considering the transition from $|m_s\rangle$ to $|m_s + 1\rangle$, the Rabi frequency is given by

$$\begin{aligned}\Omega_1 =& \frac{\gamma_e B_{mw}}{\hbar} \langle m_s + 1| S_x |m_s\rangle \\ =& \frac{\gamma_e B_{mw}}{2\hbar} \sqrt{S(S+1) - m_s(m_s+1)}.\end{aligned} \tag{3}$$

For S=1 GS, this gives a Rabi frequency of $\Omega_{S=1} = \gamma_e B_{mw}/\sqrt{2}\hbar$ and, for S=1/2 GS, the Rabi frequency is $\Omega_{S=1/2} = \gamma_e B_{mw}/2\hbar$. The multiplicities of S=1 and S=1/2 give a factor of $\sqrt{2}$ difference in Rabi frequency when the microwave field magnitude B_{mw} is fixed.

In the experiment, we compare the Rabi frequencies of II-1 and II-2 at the same input microwave power. To get rid of the frequency dependent microwave transmission which affects the Rabi frequency at different driving frequencies, we tune the magnetic field to make the resonance frequencies of II-1 and II-2 be the same. Figure S8(b) shows the Rabi oscillations of II-1 and II-2 at 98.4 mT and 62.5 mT, respectively. A ratio of 1.37 is obtained by fitting the oscillation frequencies, agreeing well with the expected ratio between S=1 (II-1) and S=1/2 (II-2).

C. Optically detected NMR and coherent control of ^{13}C nuclear spin

To understand the hyperfine interaction and gain more insight into the chemical structure of the defect, we perform the optically detected nuclear magnetic resonance (ODNMR) to detect and directly control the most strongly coupled ^{13}C nuclear spin. Figure S9 shows the measured nuclear spin resonance spectrum obtained by setting the microwave frequency in resonance with II-2 and sweeping the frequency of the RF pulse. The clear nuclear spin transitions at 65.0 MHz and 66.6 MHz are consistent with the hyperfine splitting ∼130 MHz in the ESR spectrum together with a spin-1/2 model involving in the II-2 transition (Figure S9(b)). In addition, the ODNMR spectrum also shows another broad resonance at around 9.2 MHz due to hyperfine coupling with other nearby nuclear spins.

Furthermore, by parking the RF pulse at either 65.0 MHz or 66.6 MHz and increasing the pulse duration, we obtain coherent Rabi oscillations of the proximal ^{13}C nuclear spin with Rabi frequency up to 0.7 MHz (Figure S10). We also confirm that nuclear spin Rabi frequency $\Omega_{C,Rabi}$ shows a linear dependence on the square root of the RF power.

The fast coherent control of the ^{13}C nuclear spin is enabled by the gyromagnetic ratio enhancement due to the hyperfine interaction with the defect electron spin [4]. For a S=1/2 nuclear spin that couples to a spin-1/2 electron spin, the enhancement factor is related to the electron spin state. These factors can be expressed as [5]

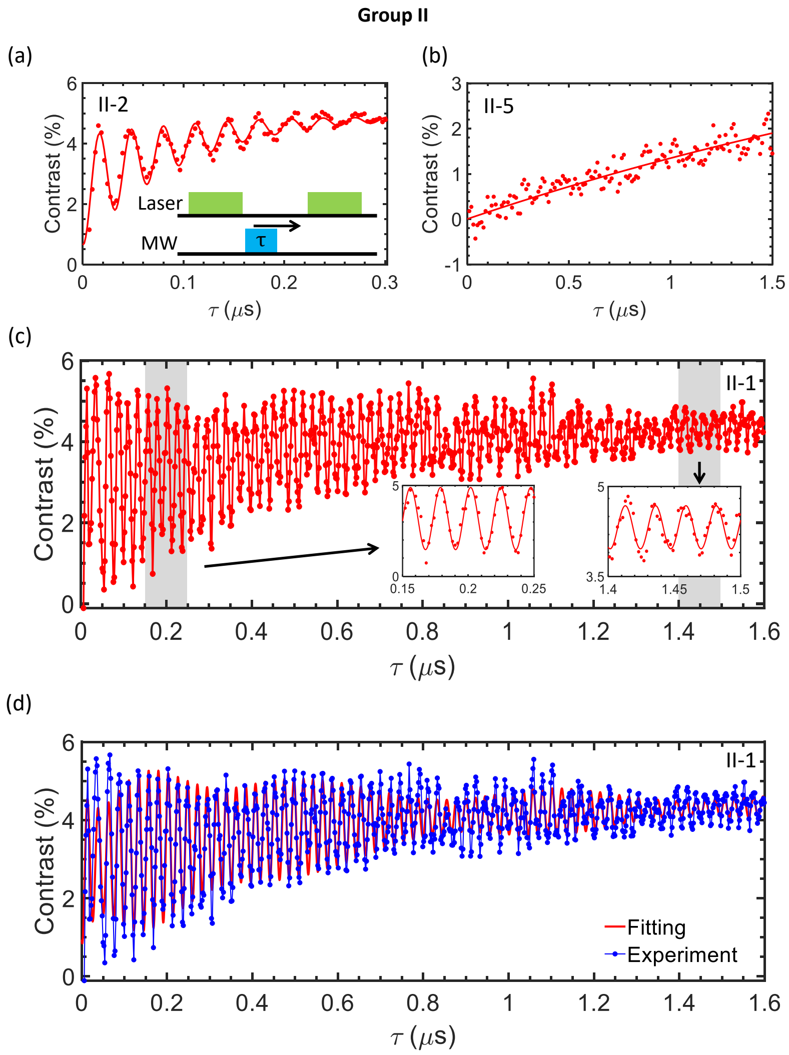

Figure S7. **Coherent control of the electron spin.** Rabi oscillation by driving the transition at the resonance (a) II-2, (b) II-5, and (c) II-1. (d) Fitting the Rabi oscillation of resonance II-1. Two oscillation frequencies are extracted into a major component of $f_{rabi,1} = 44.56$ MHz with a decay time of $T_{Rabi,II-1} = 0.75$ μs, and a minor component of $f_{rabi,2} = 43.07$ MHz with a decay time of $T_{Rabi,II-1} = 0.19$ μs.

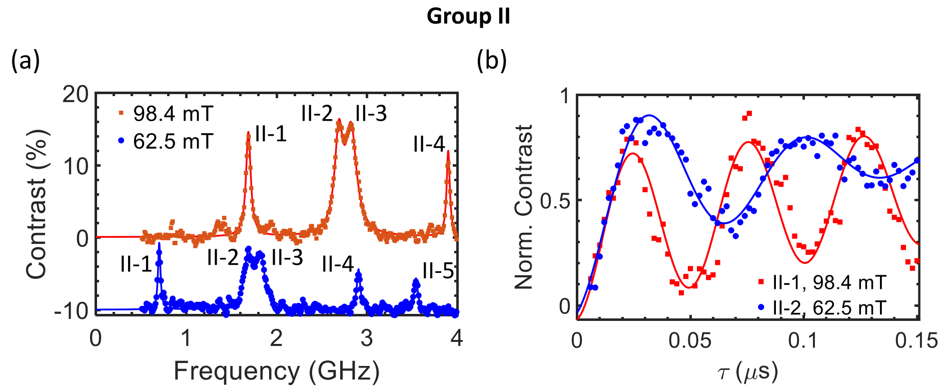

Figure S8. (a) cw-ODMR spectra measured at 98.4 mT and 62.5 mT. (b) Rabi oscillation measured at II-1 (red squares, 98.4 mT) and at II-2 (blue dots, 62.5 mT).

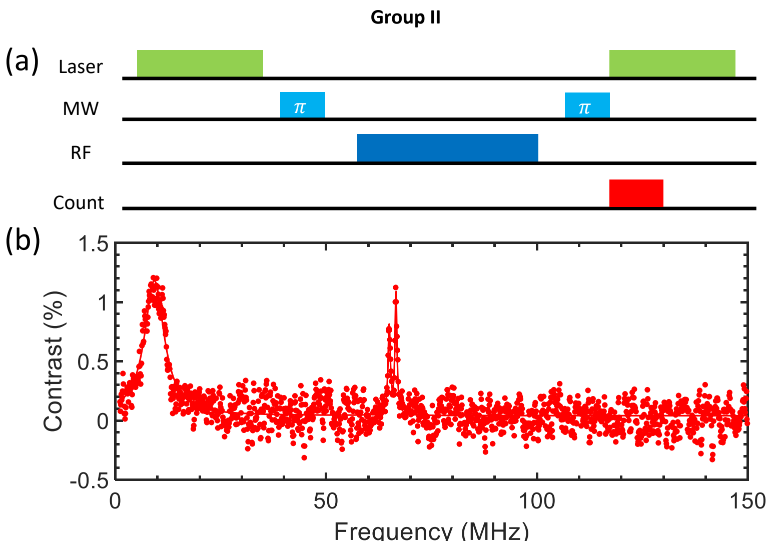

Figure S9. (a) Experimental pulse sequence (b) Resulting ^{13}C nuclear spin resonance spectra at 71.8 mT measured at the resonance II-2.

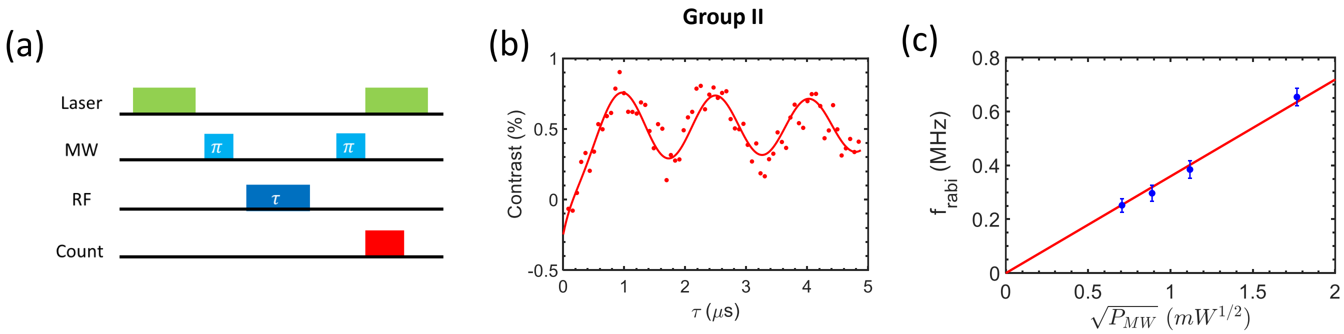

Figure S10. (a) Schematic of the pulse sequence for coherently control and optical detection of ^{13}C nuclear spins. (b) Nuclear spin Rabi measured under 35 dBm RF driving. (c) Nuclear spin Rabi frequency under different RF powers.

$$\alpha_{+1/2} = cos(\beta) + \frac{\gamma_e}{\gamma_n} sin(\beta) \tag{4}$$

$$\alpha_{-1/2} = cos(\beta) - \frac{\gamma_e}{\gamma_n} sin(\beta) \tag{5}$$

where β is given by

$$tan(2\beta) = \frac{A_\perp}{\gamma_e B_z - \gamma_n B_z}, \tag{6}$$

where $A_\perp$ is the transverse hyperfine interaction strength. In the regime of strong external magnetic field, such that $A_\perp \ll \gamma_e B_z - \gamma_n B_z$, $\alpha_{\pm 1/2}$ can be exact up to the first order:

$$\alpha_{\pm 1/2} = 1 \pm \frac{\gamma_e}{\gamma_n} \frac{A_\perp/2}{\gamma_e B_z - \gamma_n B_z}. \tag{7}$$

The transverse hyperfine coupling strength $A_\perp$ can be estimated from the enhancement factors.

D. Other examples of Group II spin defects

Figure S11 shows three spin-active defects (Defect 5-7 in Table S1 in Section VII) in Group II with the corresponding PL, ODMR and ODNMR spectra. The PL spectra show a relatively large variation of the zero phonon lines (ZPL)

over tens of nanometers. The ODMR spectra of all the three defects show both resonance from spin-1/2 and resonance from spin-1. The resonances of spin-1 transition do not always locate at the same frequencies despite the same external magnetic field. This may originate from different local strain environment at different positions in the flake or different separation of donor-acceptor pair that we propose to explain the chemical structure of the Group II spin defects. We further characterize the nuclear spin resonance at the center hyperfine peaks (Figure S11 (c),(f),(i)). All the three defects shows two resonances at nearly the same frequencies in the ODNMR spectra. Both ODMR spectra and ODNMR spectra suggest that Defect 5-7 have the similar chemical structure as Defect 2 despite different PL spectra among these defects.

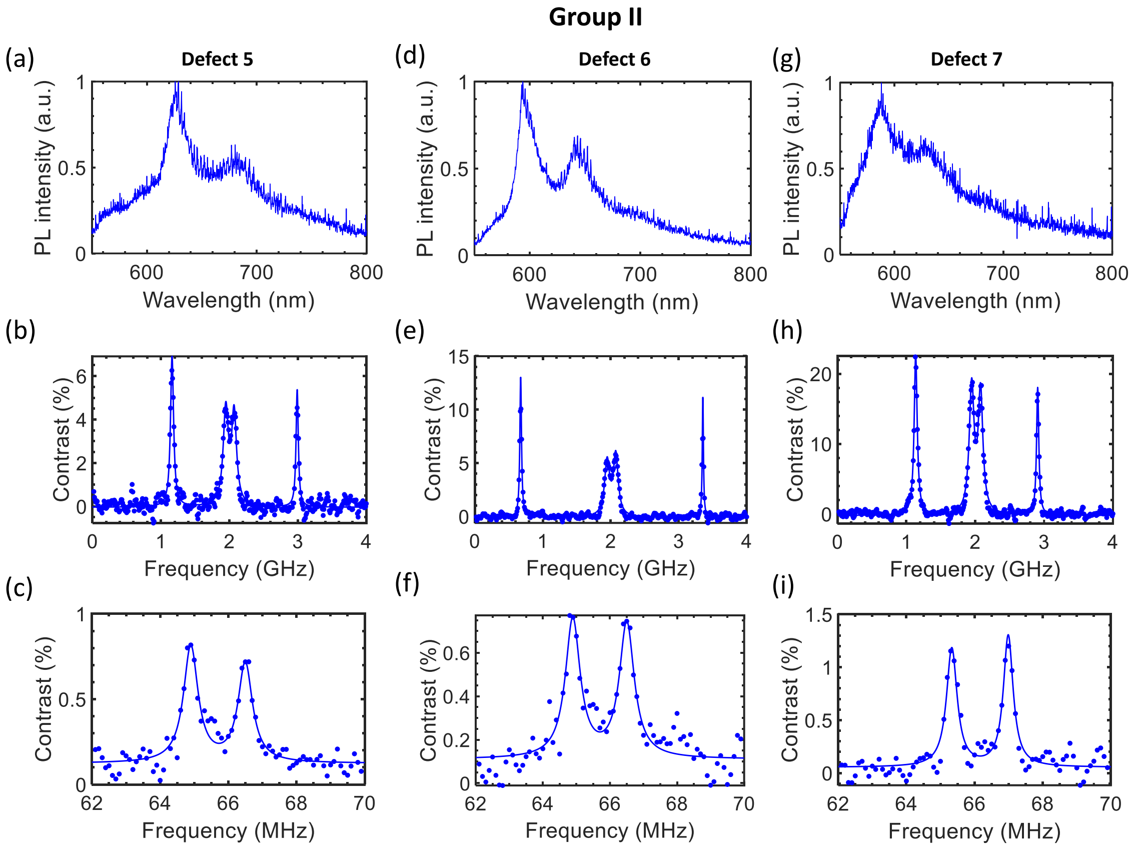

Figure S11. Other examples of Group II defects. (a) Optical, (b) ODMR and (c) ODNMR spectra of Defect 5. (d) Optical, (e) ODMR and (f) ODNMR spectra of Defect 6. (g) Optical, (h) ODMR and (i) ODNMR spectra of Defect 7. Other information about these defects can be found in Table S1 in Section VII.

V. Spin defects in Group III

A. ODMR spectroscopy

Figure S12 shows the ODMR spectra of Defect 3 (Group III, Table S1 in Section VII). In the CW ODMR, five resonances are observed: three central resonances (III-2,III-3,III-4) and two side resonances (III-1,III-5) (Figure S12(a)). A fine scan near the side resonances III-1 and III-5 using a weak microwave drive reveals that each of these resonances splits further into two peaks. This splitting, approximately 70 MHz, is attributed to hyperfine interactions with a ^{13}C nuclear spin.

Pulsed ODMR measurements were also performed to identify the ground-state spectroscopy. Unlike the Group II defects, Defect 3 exhibits only the central three resonances (III-2 to III-4). In this measurement, the waiting time between two laser pulses is set to 100 ns, indicating that III-1 and III-5 involve short-lived states with lifetimes significantly shorter than 100 ns.

The evolution of the ODMR spectrum as a function of an externally applied magnetic field is shown in Figure S12(c). The magnetic field is aligned to out-of-plane axis. The frequencies of III-2 to III-4 are linearly proportional to the magnitude of the magnetic field without obvious ZFS parameters. III-1 and III-5 shows a ZFS of D = 950 MHz and E = 300 MHz.

Based on these results, it is speculated that resonances III-2 to III-4 originate from a spin-1/2 system, while III-1 and III-5 correspond to a spin-1 system. The spin-1/2 origin will be further verified in subsequent discussions using ODNMR measurements. However, since III-1 and III-5 are not observed in the pulsed ODMR spectrum, no additional experiments were conducted to confirm their spin multiplicity.

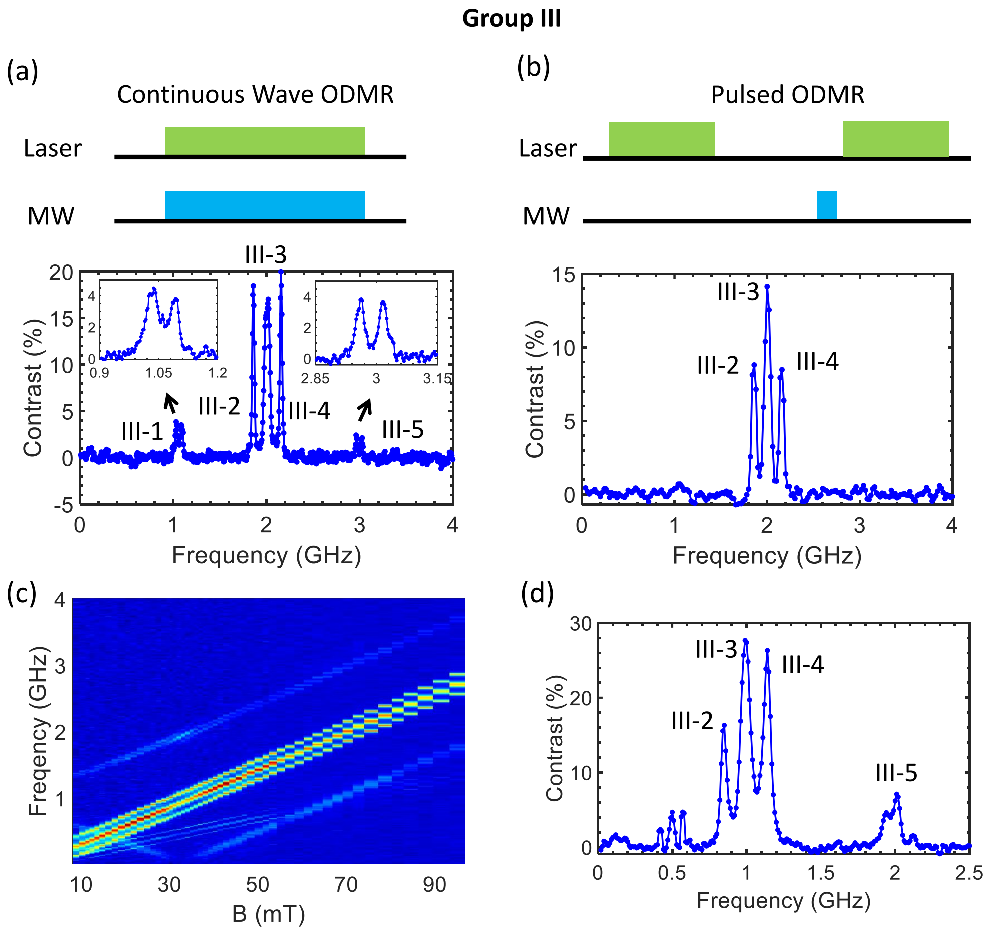

Figure S12. **ODMR of Defect 3 in Group III.** (a) CW-ODMR spectrum measured at 71.8 mT. (b) Pulsed ODMR of Defect 3 measured at 71.8 mT. (c) CW-ODMR spectrum as a function of magnetic field. (d) CW-ODMR spectrum measured at 35.4 mT.

Interestingly, when we scan the magnetic field near the LAC of III-1, we observe an imbalanced amplitudes in the

ODMR spectrum between III-2 and III-4 (Figure S12(c)-(d)). This imbalance hints a finite nuclear spin polarization induced by the electron spin near LAC. However, such an imbalance is observed only near 35.4 mT when III-1 goes to zero. Either increase or decrease the magnetic field will reduce the imbalance and finally make it disappear. This hints that the nuclear spin polarization is induced by the LAC of resonance III-1, but is detected by the electron spin involved in III-2 and III-4. Additionally, the contrast of all transitions decrease near the LAC of III-1. These results suggest a strong relation among these transitions and need more investigation to better comprehend this complex dynamics.

We further characterize the GS ODMR spectroscopy at different microwave powers to obtain the best ODMR contrast of Defect 3. Figure S13 (a) shows the contrasts of III-2 to III-4 as a function of microwave power. Strikingly, ODMR contrasts of resonance III-2(III-4) and III-3 reach 40% and 60% , respectively (Figure S13(d)). A significant power broadening is also observed under the strong microwave driving (Figure S13(b)). By applying a weak microwave (0.3 mW), we extract the natural ODMR linewidth of 16 MHz and 25 MHz for III-2 and III-3, respectively. In this condition, a splitting with a separation of 34 MHz at III-3 can be resolved (III-3a and III-3b in Figure S13(c)). The splitting of III-3 can originate from the hyperfine coupling with a ^{13}C nuclear spin. This hints that there are two spin GS with different hyperfine coupling strengths contributing to the center three resonances. Therefore, we anticipate that III-2 and III-4 are from the same spin system with a hyperfine coupling strength of $\sim$ 300 MHz, while III-3 originates from another spin system with a relatively weaker hyperfine coupling strength (34 MHz). The two spin systems may come from a closely spaced two defects or different charge states of the same defect.

The high ODMR contrast and relatively narrow linewidth give rise to an improved sensitivity for sensing application. Here we take DC magnetic field sensing as an example. The DC magnetic field sensitivity can be written as

$$\eta_{DC} = \frac{8\pi}{3\sqrt{3}\gamma_e}\frac{\delta\nu}{C\sqrt{I}}, \tag{8}$$

where I denotes the photon count rate, $\delta\nu$ is the ODMR linewidth and C is the contrast. Given the 170 kcts/s photon count rate of Defect 3 under 15 μW laser excitation, we obtain an optimized senstivity of 5 $\mu T/\sqrt{Hz}$ if we use III-2(III-4) for the measurement (Figure S14).

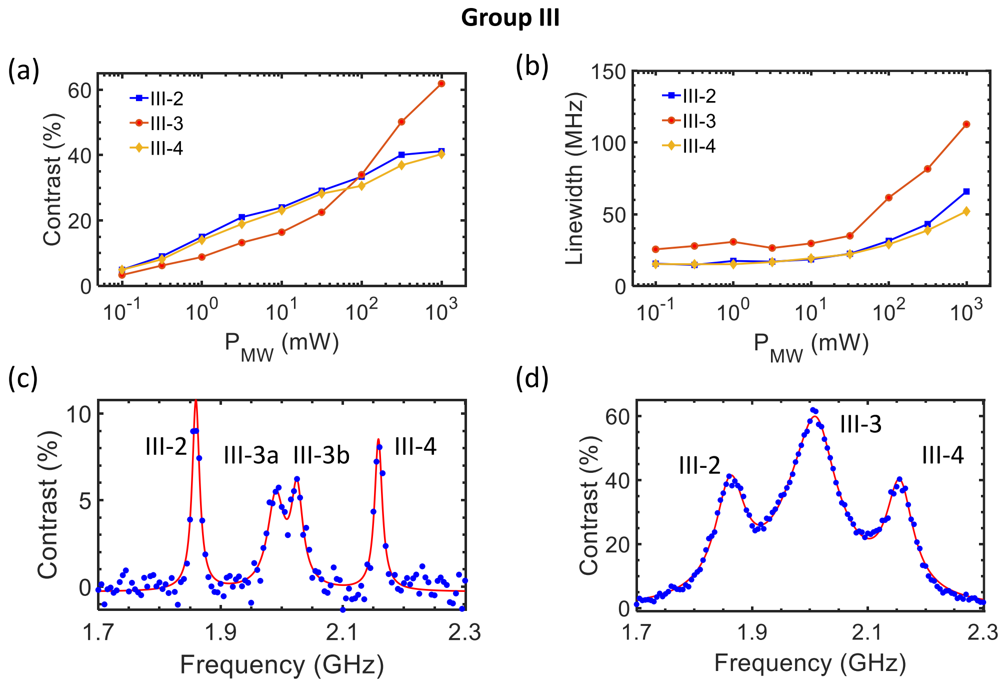

Figure S13. **ODMR of as a function of microwave power.** (a) ODMR contrast as a function microwave power. (b) ODMR linewidth as a function microwave power. The linewidth of III-3 for $P_{MW} \geq 100$ mW is obtained by using single lorentzian fitting. The linewidth of III-3 for $P_{MW} \leq 100$ mW is obtained by using double lorentzian fitting and taking the average of the linewidths of two hyperfine peaks III-3a and III-3b (as shown in (c)). (c) CW-ODMR spectrum with weak microwave driving (0.3 mW). Two hyperfine peaks with a separation of 34 MHz are resolved at the center resonance III-3. (d) CW-ODMR spectrum measured with 1 W microwave driving.

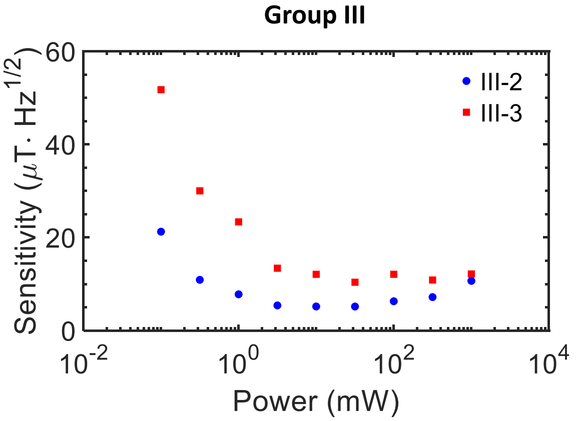

Figure S14. DC magnetic field sensitivity as a function of the microwave power. Laser power is fixed at 15 μW.

B. Spin coherence and readout efficiency

Next, we characterize the spin coherence properties of Defect 3. As shown in Figure S15, the defects shows a high contrast ($\sim$30%) Rabi ocillation signal with a decay time of T_{Rabi} = 169 ns. More impressively, the relaxometry measurement give a strikingly long T_1 of 144 μs, a order of magnitude higher than the V_B^- defects in hBN. Figure S15(c) presents spin coherence measurements using Hahn Echo sequence using a single refocusing π pulse. The extracted coherence time is 131 ns, comparable to that of V_B^- defects.

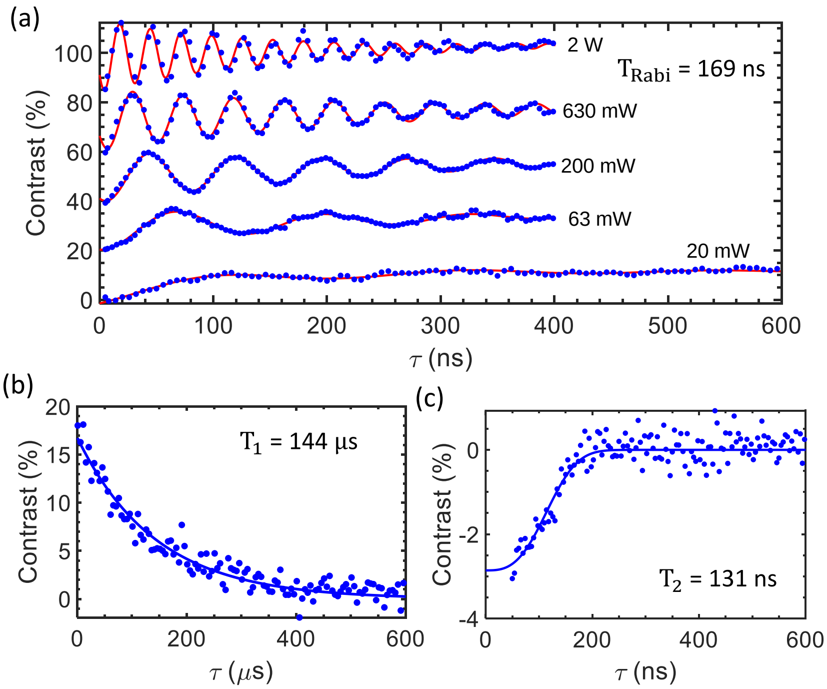

Figure S15. **Spin coherence of Defect 3 in Group III.** (a) Coherent control of the electron spin resonance at III-2. The defects shows a high contrast ($\sim$30%) Rabi oscillation signal with a decay time of $T_{Rabi,e}$ = 169 ns. (b) $T_{1,e}$ relaxation measurement at III-2, showing a relaxation time of 144 μs. (c) Hahn Echo measurement revealing $T_{2,e}$ = 62 ns.

We also observe that the Rabi oscillations of Group III defects exhibit significantly lower asymmetry compared to other defect groups. As microwave power increases, the asymmetry becomes even less pronounced. This subtle asymmetry suggests the possibility of a GS spin-1/2 manifold, where minor asymmetry could arise from an inhomogeneous hyperfine environment created by surrounding nuclear spins or from the transfer of population between a metastable state and the ground state. In the latter case, this effect can be largely canceled through differential measurements

by performing a reference measurement using the same pulse sequence but without a microwave pulse, as done in our experiments.

As microwave power increases, the transverse driving field becomes stronger, reducing the relative influence of the static magnetic field in the z-direction caused by nuclear spin coupling, leading to an overall increase in the Rabi contrast. At low microwave power, the contrast is smaller, making the Rabi asymmetry more prominent.

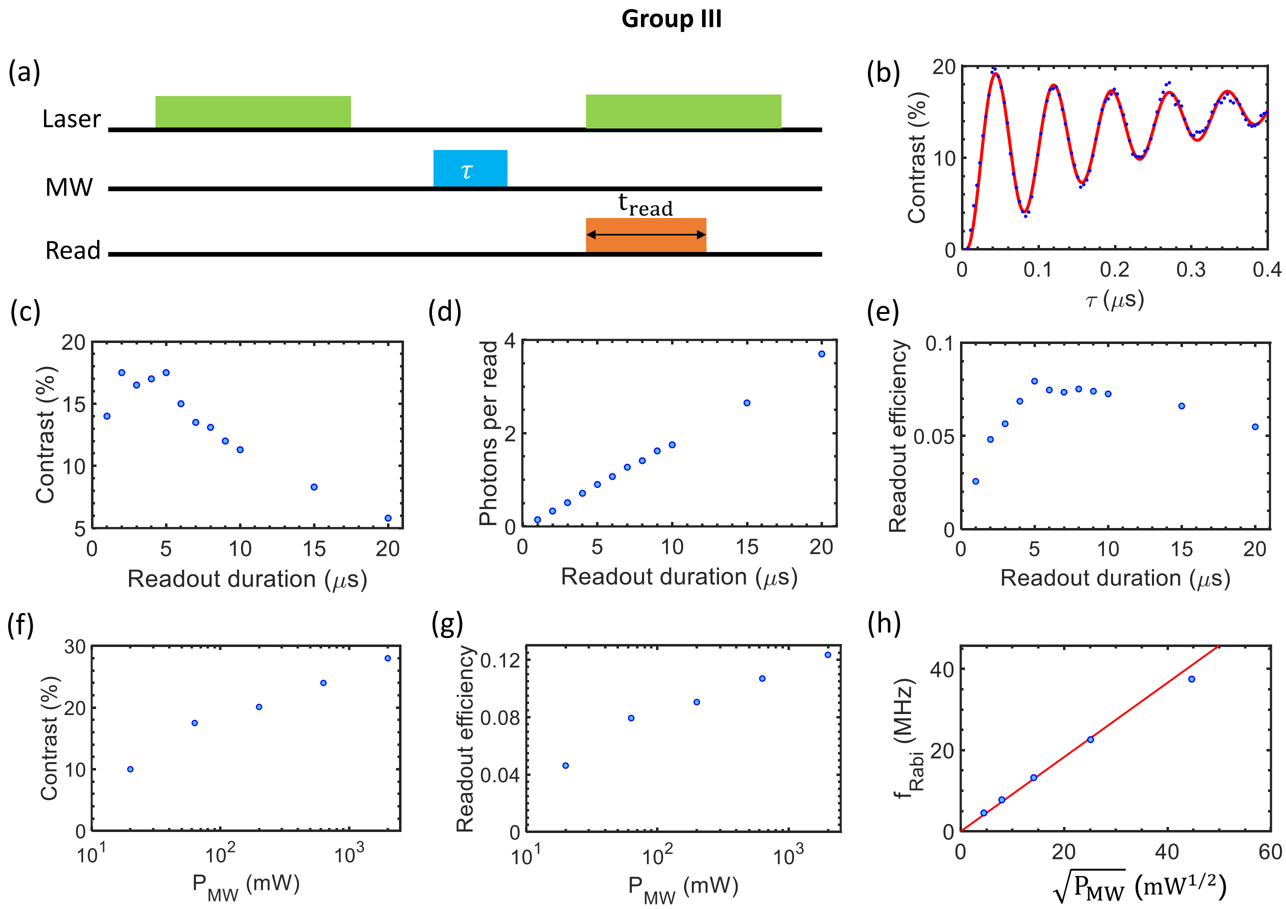

Figure S16. **Optimizing electron spin readout efficiency of Defect 3 in Group III.** (a) A schematic of the pulse sequence to estimate the readout efficiency. Rabi measurements are performed by tuning the microwave duration τ at different readout times t_{read}. (b) An example of Rabi oscillation measured at 71.8 mT. (c) ODMR contrast of the Rabi oscillation as a function of readout duration time t_{read}. (d) Number of photons collected from the darker state in each readout pulse as a function of t_{read}. (e) Estimated electron spin readout efficiency as a function of t_{read}. (f) ODMR contrast of the Rabi oscillation as a function of microwave power P_{MW}. (g) Estimated readout efficiency as a function of P_{MW}. (h) Rabi frequency as a function of $\sqrt{P_{MW}}$. The laser power is fixed at 15 μW.

Now, we estimate the spin state readout efficiency by estimating the signal to noise (SNR) ratio of a single optical readout pulse in Rabi measurements. The SNR takes the form [6]:

$$SNR = \frac{\alpha_0 - \alpha_1}{\sqrt{\alpha_0 + \alpha_1}}, \tag{9}$$

where α_0 and α_1 are the mean numbers of detected photons for a single measurement of the brighter state and darker state, respectively. The dimensionless spin-readout uncertainty of a single readout measurement, σ_s, is directly related to SNR, following the equation [6]:

$$\sigma_s = \sqrt{1 + \frac{2}{SNR^2}} = \sqrt{1 + 2\frac{\alpha_0 + \alpha_1}{(\alpha_0 - \alpha_1)^2}} \tag{10}$$

After N times of repetitive measurements and averaging, the uncertainty can be reduced to

$$\sigma_N = \frac{\sigma_s}{\sqrt{N}}. \tag{11}$$

To make the uncertainty σ_N at 1, a number $n = (1/\sigma_s)^2$ of measurements are required. Therefore, here we define the single-time readout fidelity by making an inverse of the spin readout noise for a single measurement:

$$\eta_s = 1/\sigma_s = \left(1 + 2\frac{\alpha_0 + \alpha_1}{(\alpha_0 - \alpha_1)^2}\right)^{-1/2}. \tag{12}$$

Figure S16 presents the optimization results to maximize the readout efficiency by tuning the readout duration time and microwave power in the pulsed ODMR experiments. The pulsed ODMR contrast reach ∼18% when the readout duration is 5 μs under 15 μW laser pumping. If we further ramp up the readout duration time, the contrast starts decreasing. The long readout time window hints a relatively long-live metastale state involved in the spin polarization process. In addition, we see a monotonic increase of the contrast when we increase the microwave power. Finally, the readout efficiency reaches up to 0.12 in a single-time spin-state measurement when we apply a 2 W microwave drive.

C. Optically detected nuclear spin magnetic resonance

To understand the hyperfine interaction of Defect 3, we perform the optically detected nuclear magnetic resonance (ODNMR) measurements. Figure S17 shows the measured nuclear spin resonance spectrum obtained by setting the microwave frequency in resonance with III-4 and sweeping the frequency of the RF pulse. The clear nuclear spin transitions at 147.3 MHz and 151.4 MHz are consistent with the hyperfine splitting ∼300 MHz in the ESR spectrum together with a spin-1/2 model involving in the III-2 transition (Figure S9 (b),(c)). This resonance is also observed when the microwave is resonance with III-2. However, if the microwave frequency is set in resoance with III-3, no ODNMR spectrum is observed near 150 MHz. Instead, we observe two clear resonances near 16 MHz. The different resonance frequencies observed at III-2(III-4) and III-3 hint that III-2(III-4) and III-3 belong to two S = 1/2 spin systems that couples to nuclear spins with different strengths.

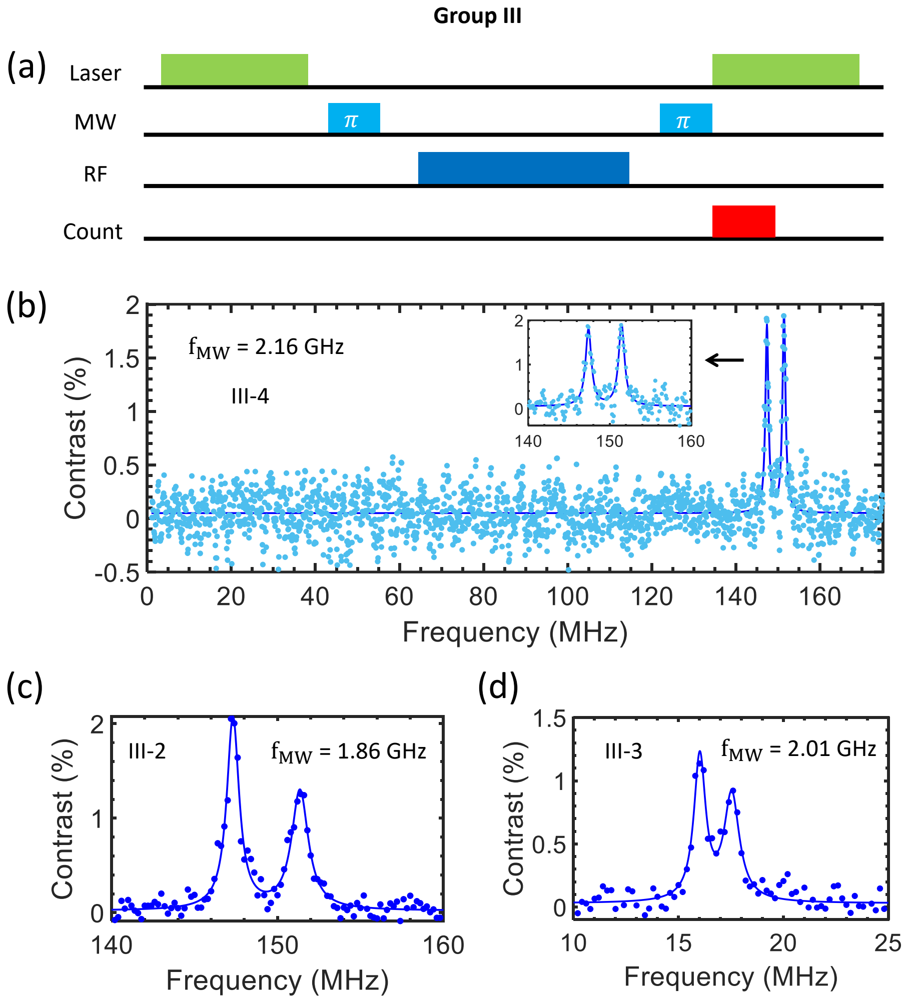

Figure S17. **ODNMR of Defect 3 in Group III.** (a) A schematic of the pulse sequence. (b)-(d) ODNMR spectra when the microwave is parked in resonance wtih (b) III-4 (c) III-2 and (d) III-3. The measurements are perform at 71.8 mT.

D. Coherent control of ^{13}C nuclear spin using Defect 10

In the main text, we present the optical detection and coherent control of a ^{13}C nuclear spin using Defect 10. Here we present one more example of nuclear spin coherent control using Defect 10. As shown in Figure S18, the ODMR contrasts of resonances III-2 to III-4 reaches 33 % and the T_1 is 156 μs. By performing the same pulse sequence shown in Figure 3 in the main text, we achieve a nuclear spin polarization of 60%.

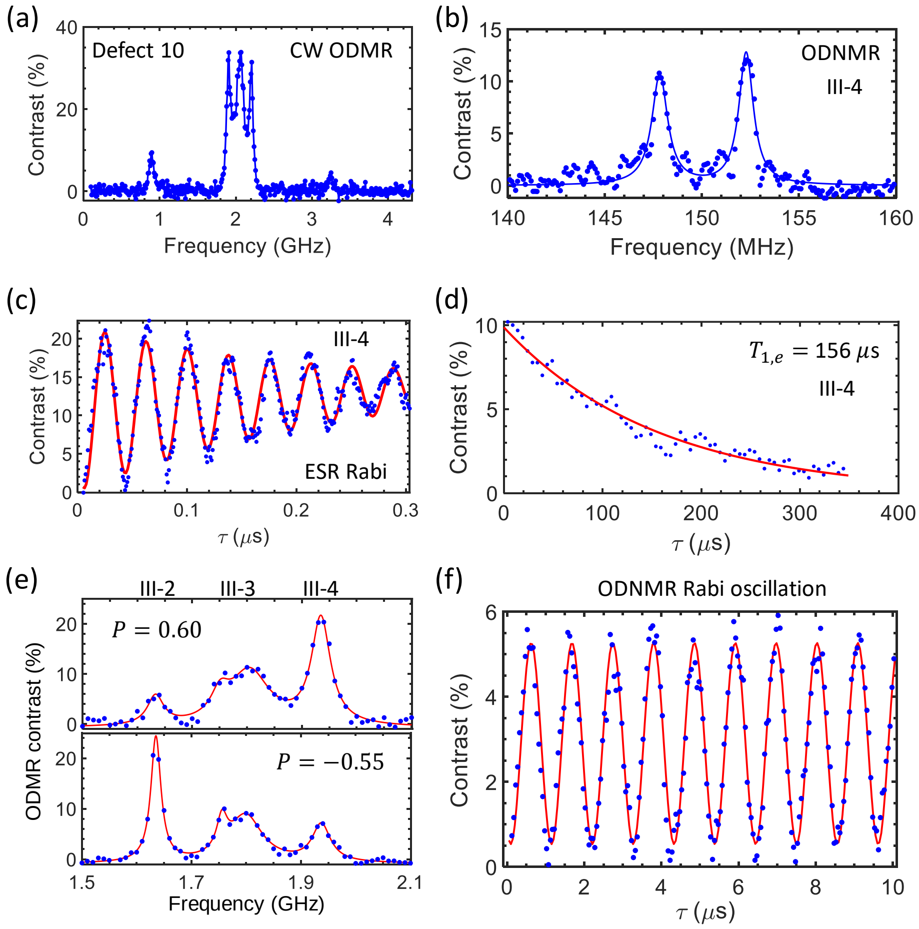

Figure S18. **Coherent control of a nuclear spin using Defect 10** (a) The electron spin ODMR spectrum of Defect 10. (b) ODNMR spectrum measured at III-2. (c) Electron spin Rabi oscillation. (d) T_1 relaxation time at room temperature. (e) Estimation of nuclear spin polarization by measuring the ODMR spectrum after the SWAP gate. (f) ^{13}C nuclear spin Rabi oscillation.

E. Other examples of Group III spin defects

Figure S19 shows two additional examples of Group III defects (Defect 8-9 in Table S1 in Section VII). Their ODMR spectra exhibit three central peaks, although the relative intensities of III-2 and III-3 differ between defects. In particular, the ODMR spectrum of Defect 9 shows a significantly weaker contrast at III-3 compared to III-2 and III-4. Under strong microwave driving (1 W), III-3 becomes unresolved due to power broadening, resulting in only two prominent peaks at the center. When the microwave power is reduced to mitigate broadening effects, a low-contrast resonance at III-3 reappears.

The ODNMR of both defects, shows two peaks at 146 MHz and 150 MHz, respectively. We note that the ODNMR frequencies of Group III defects can vary by 5-10 MHz, likely due to local variations in the spin wavefunction caused by strain or nearby defects. The overall agreement in both ODMR and ODNMR spectra supports the conclusion that Defects 8 and 9 share the same chemical structure as Defect 3.

In addition, we can also observe single Group III spin defects exhibiting negative contrast as shown in Figure S20. Such defects are rarely seen in our samples and typically do not show measurable contrast in pulsed ODMR experiments even with dark times on the order of hundreds of nanoseconds between laser pulses. This behavior suggests differences in their optical cycling or spin initialization dynamics compared to the more common positive-contrast defects.

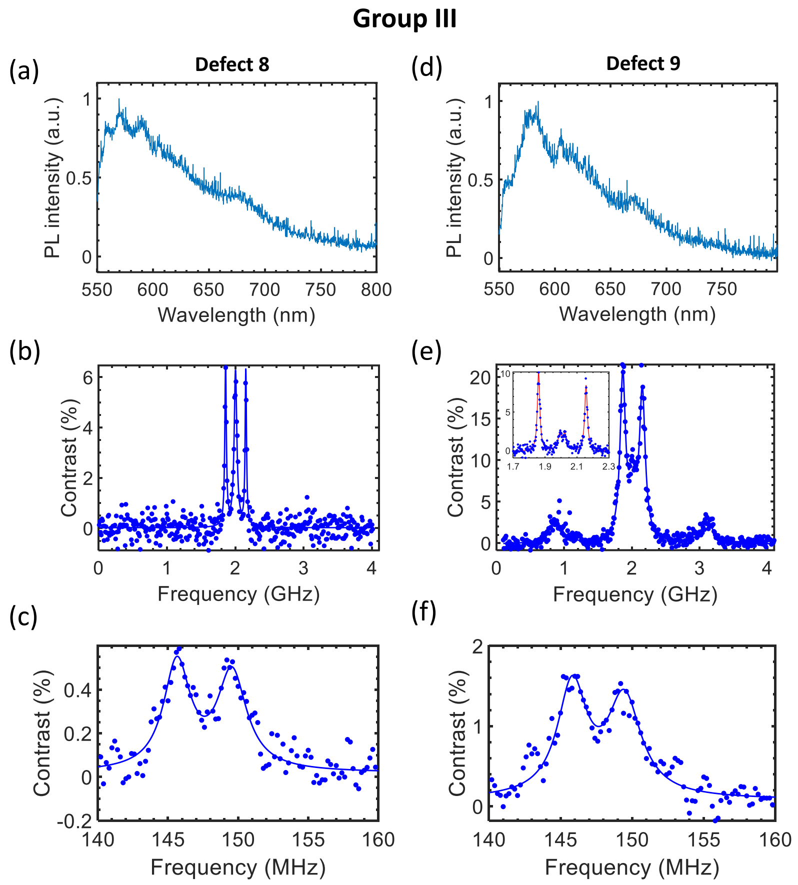

Figure S19. Other examples of Group III defects. (a) Optical, (b) ODMR and (c) ODNMR spectra of Defect 8. (d) Optical, (e) ODMR and (f) ODNMR spectra of Defect 9. The inset of (e) shows the center branch of Defect 9 taken under a weak microwave drive, which reveals the center resonance III-3 with a hyperfine splitting ($A_{zz} \approx 30$ MHz). All the ODNMR measurements are taken at 71.5 mT at resonance III-2 (ν=1.86 GHz). Other information about these defects can be found in Table S1 in Section VII.

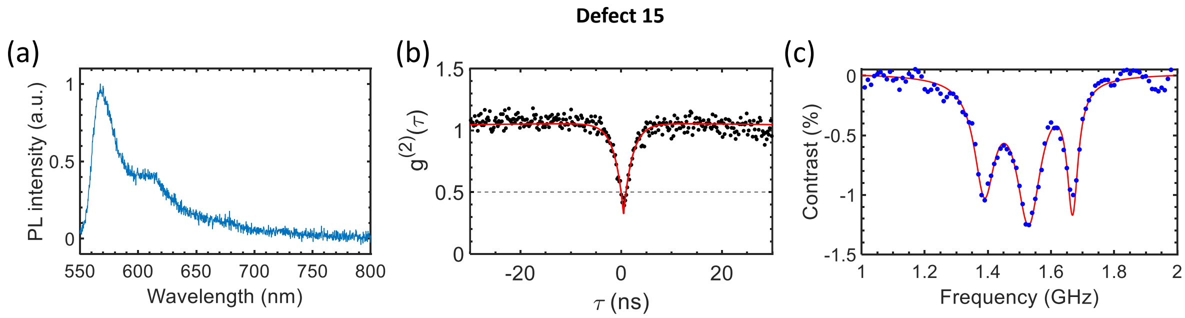

Figure S20. (a) Optical spectrum of Defect 15. (b) Second-order intensity-correlation measurement ($g^{(2)}(\tau)$) showing a single defect. (c) A CW ODMR spectrum measured at 54.5 mT, showing a negative contrast.

VI. Spin defects in Group I

A. GS ODMR spectroscopy and spin coherence properties

In the $^{13}CO_2$ implanted hBN flakes, the most frequently observed spin defects belong to Group I, which do not show hyperfine splitting in their ODMR spectra. This absence of hyperfine splitting indicates weak or no coupling to the ^{13}C nuclear spins. These defects are likely associated with intrinsically present carbon impurities, which are predominantly composed of naturally abundant ^{12}C.

The stability of Group I defects varies across different defects. More than half of the observed defects display blinking behavior during ODMR measurements and may quench within a few minutes. In contrast, the stable defects remain optically active throughout the entire duration of the measurements when using weak laser excitation (15 μW), and can persist without degradation for several weeks under continuous experimental conditions.

Here, we characterize the stable spin defects in Group I to gain more insight into their properties. Figure S21 shows the ODMR spectra of Defect 4 (Table S1 in Section VII). Both CW ODMR and pulsed ODMR reveal three resonances. The center resonance does not show hyperfine structure. In Figure S22, we present CW ODMR measurements at different microwave powers. The ODMR contrast reaches up to 70% at 1 W microwave power. The best DC magnetic field sensitivity of Defect 4 is 4.7 μT·Hz$^{-1/2}$. Under a weak microwave driving ($\leq$ 1 mW), the linewidth of I-1 and I-2 are $<$ 20 MHz and no hyperfine structure is observed in any resonances. Therefore, we expect no ^{13}C atoms to be directly involved in the Group I defects, as the hyperfine coupling strength with ^{13}C nuclear spins are expected to be $>$ 50 MHz for point defects containing ^{13}C atoms according to the *ab initio* calculations [7].

We further investigate the spin coherence properties of Group I defect. As presented in Figure S23, Defect 4 shows clear coherent oscillation at all three resonances in the Rabi measurements. The central resonance (I-2) shows a long relaxation time up to 216 μs, while the two side resonances (I-1,I-3) show a relatively short T_1 of $\sim$ 6 μs. The coherence times of I1-I3 measured by Hahn Echo sequence are approximately 120 ns.

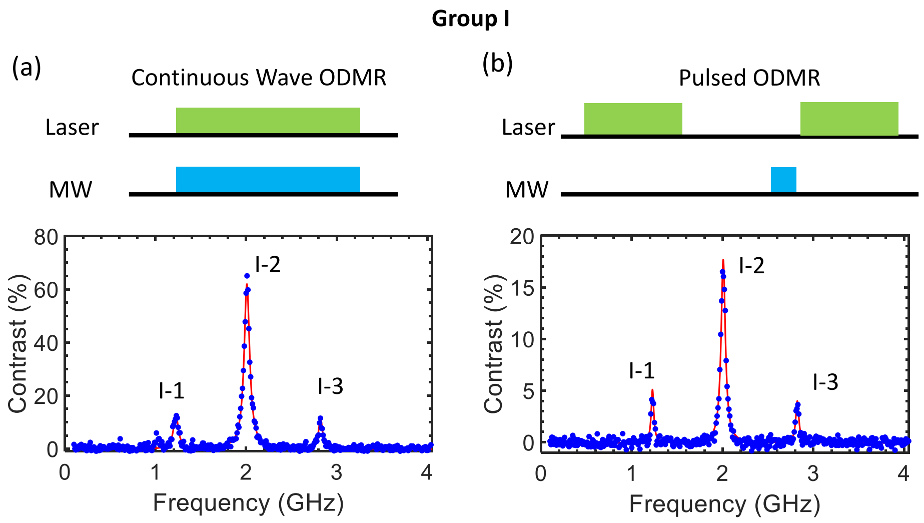

Figure S21. **ODMR of Defect 4 in Group I.** (a) CW-ODMR spectrum measured at 71.8 mT. (b) Pulsed ODMR spectrum measured at 71.8 mT. Laser power is fixed at 15 μW.

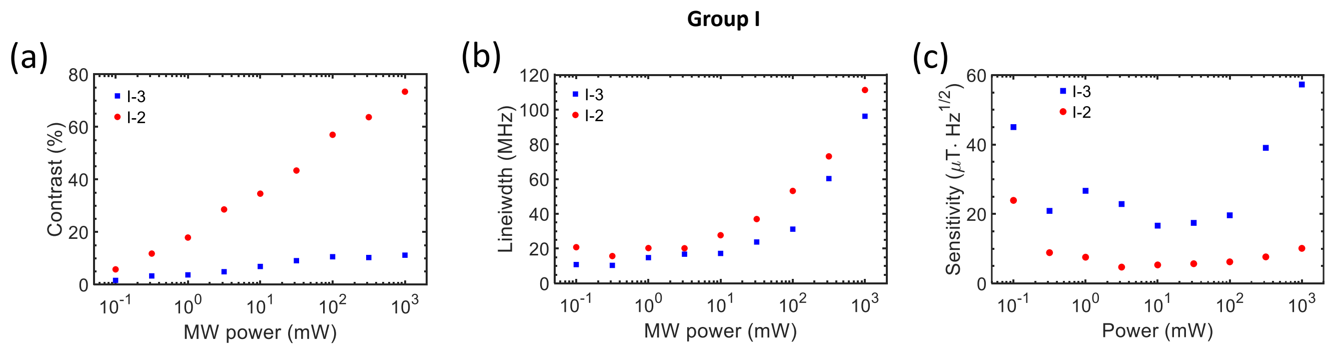

Figure S22. **ODMR of Defect 4 in Group I as a function of the microwave power.** (a) CW-ODMR contrast as a function of microwave power. (b) ODMR linewidth as a function of microwave power. (c) DC magnetic field sensitivity measured at resonance I-2 and I-3. Laser power is fixed at 15 μW.

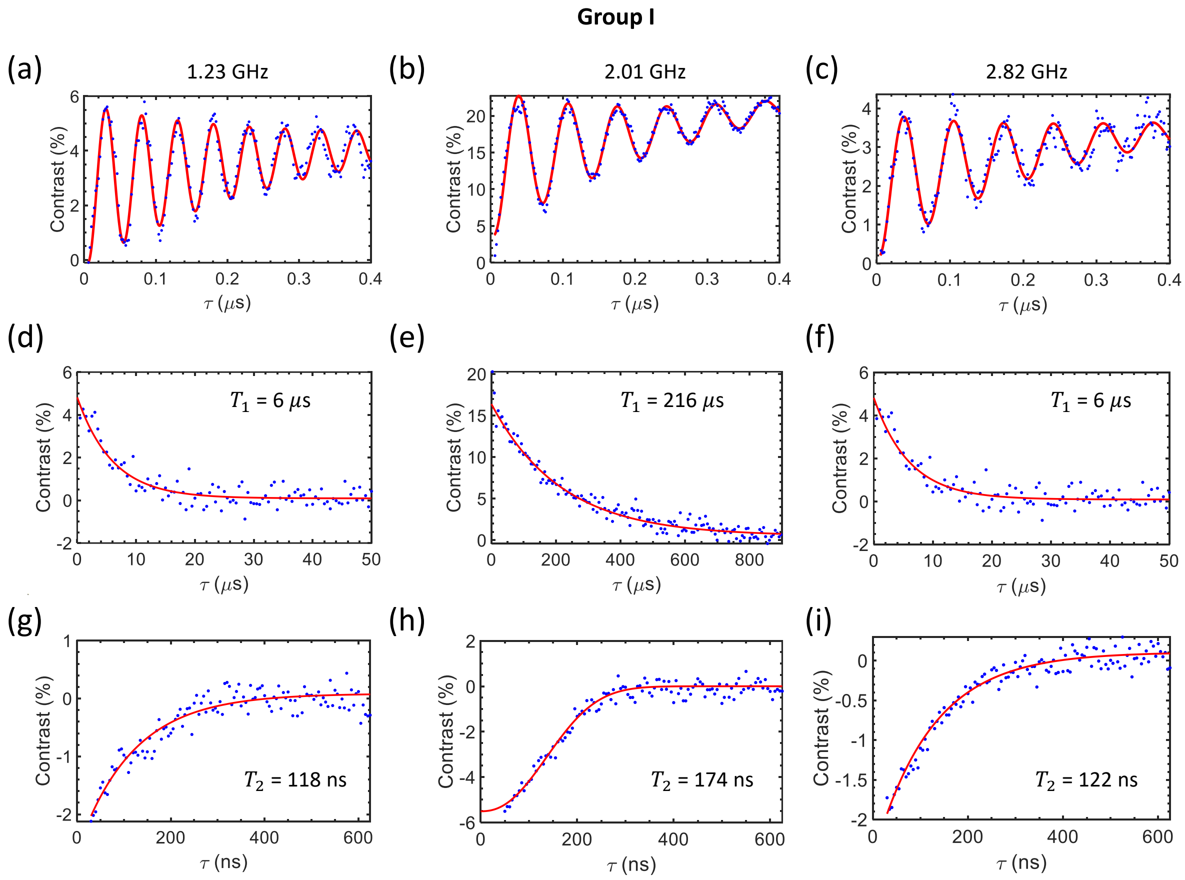

Figure S23. **Coherence properties of Defect 4 in Group I.** (a)-(c) Rabi oscillations measured at (a) I-1, (b) I-2 and (c) I-3. (d)-(f) T_1 relaxations measured at (d) I-1, (e) I-2 and (f) I-3. (g)-(i) T_2 measured by Hahn Echo sequence at (g) I-1, (h) I-2 and (i) I-3. Laser power is fixed at 15 μW. A 71.8 mT external magnetic field is applied.

B. Other examples of Group I defects

Figure S24 shows four additional examples (Defect 10-12 in Table S1 in Section VII) of Group I defects. The CW ODMR contrast can reach up to 200%, meaning that the photoluminescence with the microwave on is three times brighter than when the microwave is off. Additionally, the Rabi measurement also shows a remarkably high ODMR

contrast of 100 %.

However, in the Rabi measurements, Defects 11 and 13 show poor coherence and irregular oscillation profiles (characteristic of beating patterns) at the central resonance I-2. Instead of well-defined oscillations, the signal displays a sharp initial increase in contrast upon microwave excitation, followed by a gradual rise with ambiguous modulation as the microwave duration increases. These behaviors suggest a metastable-state configuration of a spin-pair system, which will be discussed in detail in Section XII.

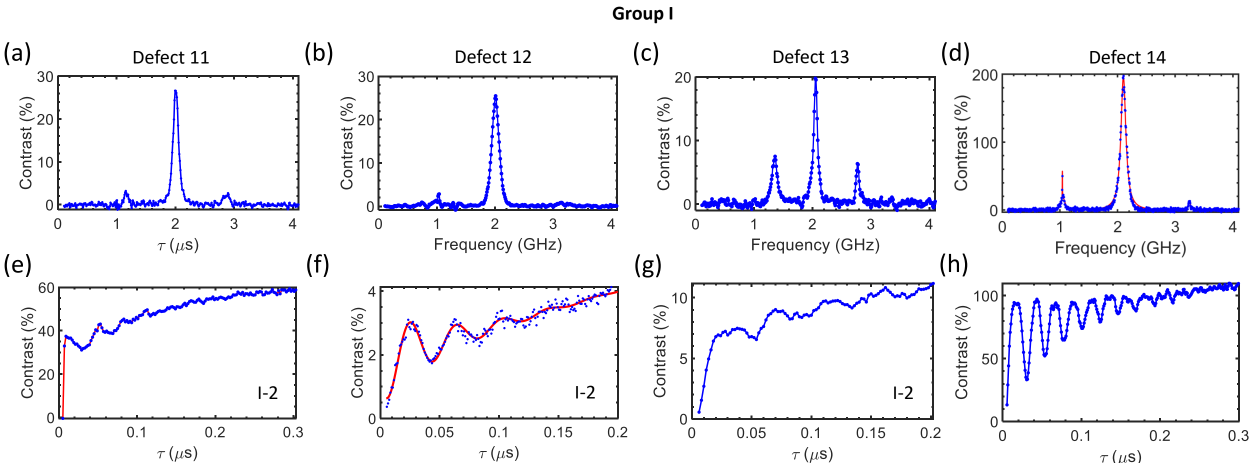

Figure S24. **Other defects in Group I.** (a)-(d) ODMR spectra of Defect 11-14. (e)-(h) Rabi oscillations of Defect 11-14.

C. Variation in Zero-Field Splitting (D) of S = 1 Transitions

The ZFS parameters D and E for S = 1 transitions exhibit noticeable variations across different defects. While local strain is often considered the primary source of these variations, another contributing factor may be the alignment of the external magnetic field relative to the defect's intrinsic quantization axis. Unlike diamond NV centers or hBN V_B^- defects, which exhibit clear ODMR contrast at zero field, the carbon-related defects in our samples show no detectable signal without an applied magnetic field. As a result, the determination of ZFS relies on fitting of ODMR spectra at finite magnetic fields. In such cases, a small misalignment between the applied field and the quantization axis could introduce a bias in the estimated ZFS splitting. Magnetic-field-dependent ODMR measurements of selected defects indicate a misalignment of approximately ±3° from the expected z-axis, likely due to local distortions such as wrinkles in the hBN flake. This misalignment introduces an estimated uncertainty of approximately 150 MHz (∼15%) in the determination of D. To account for these effects, we include this uncertainty in the D values in Table S1.

Here we also note that the ZFS D exhibit a large variation across defects, as listed in Table S1, exceeding what can be accounted for by angular misalignment alone. For example, Figure S25 shows a Group I defect with a noticeably smaller ZFS D, suggesting additional contributing factors such as local strain variations or subtle differences in defect chemical composition. These observations underscore the influence of the local hBN environment in shaping the spin properties of the defects.

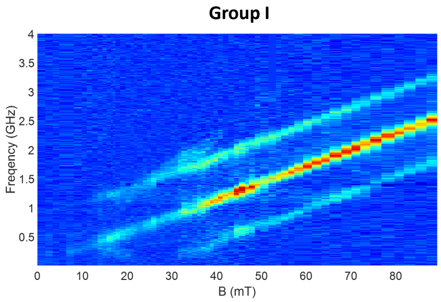

Figure S25. An example of magnetic field dependent ODMR for a Group I defect with a relatively small ZFS parameter D.

VII. Summary of defect examples

List of defect examples in $^{13}CO_2$ implanted hBN									
Defects	Group	Counts	λ_{peak}	Spin	Contrast	$T_{1,e}$	$T_{2,e}$	D	A_{zz}
D1	I	110 kcts/s	604 nm	Center(I-2): S=1/2	50%	18 μs	100 ns	-	-
				Side(I-1,I-3): S=1	3%			1.07±0.16 GHz	-
D2	II	130 kcts/s	704 nm	Center(II-2,II-3): S=1/2	9%	17 μs	89 ns	-	130 MHz
				Side(II-1,II-4): S=1	9%	33 μs	109 ns	1.1±0.16 GHz	-
D3	III	170 kcts/s	602 nm	Center(III-2,III-4): S=1/2	35%	144 μs	131 ns	-	299 MHz
				Center(III-3): S=1/2	50%	146 μs	96 ns	-	34 MHz
				Side(III-1,III-5): S=1 (*)	14%			0.95±0.14 GHz	
D4	I	180 kcts/s	579 nm	Center(I-2): S=1/2	65%	216 μs	174 ns	-	-
				Side(I-1,I-3): S=1	11%	6 μs	120 ns	0.8±0.12 GHz	-
D5	II	210 kcts/s	627 nm	Center(II-2,II-3): S=1/2	7%	18 μs		-	130 MHz
				Side(II-1,II-4): S=1	6%	17 μs		0.92±0.14 GHz	-
D6	II	170 kcts/s	594 nm	Center(II-2,II-3): S=1/2	14%	24 μs		-	130 MHz
				Side(II-1,II-4): S=1	12%	26 μs		1.34±0.2 GHz	-
D7	II	290 kcts/s	587 nm	Center(II-2,II-3): S=1/2	29%	8.8 μs		-	130 MHz
				Side(II-1,II-4): S=1	30%	8.8 μs		0.89±0.13 GHz	-
D8	III	120 kcts/s	571 nm	Center(III-2,III-4): S=1/2	6%	114 μs	116 ns	-	296 MHz
				Center(III-3): S=1/2	7%	115 μs	150 ns	-	
				Side(III-1,III-5): S=1 (*)	0.6 %			1.05±0.16 GHz	
D9	III	130 kcts/s	579 nm	Center(III-2,III-4): S=1/2	22%	37 μs	101 ns	-	296 MHz
				Center(III-3): S=1/2	11%			-	~30 MHz
				Side(III-1,III-5): S=1 (*)	3%			1.1±0.16 GHz	~70 MHz
D10	III	110 kcts/s	610 nm	Center(III-2,III-4): S=1/2	33%	156 μs		-	300 MHz
				Center(III-3): S=1/2	33%	132 μs		-	~51 MHz
				Side(III-1,III-5): S=1 (*)	10%			1.1±0.16 GHz	
D11	I	170 kcts/s	659 nm	Center(I-2): S=1/2	26%	17 μs		-	-
				Side(I-1,I-3): S=1	3%			0.85±0.13 GHz	-
D12	I	140 kcts/s	586 nm	Center(I-2): S=1/2	26%	24 μs	92 ns	-	-
				Side(I-1,I-3): S=1					
D13	I	450 kcts/s	702 nm	Center(I-2): S=1/2	20%	75 μs		-	-
				Side(I-1,I-3): S=1	8%			0.7±0.1 GHz	-
D14	I	450 kcts/s	579 nm	Center(I-2): S=1/2	200%	91 μs		-	-
				Side(I-1,I-3): S=1	50%			1.1±0.16 GHz	-
D15	III	150 kcts/s	568 nm	Center(III-2,III-4): S=1/2	1%			-	300 MHz
				Center(III-3): S=1/2	1%			-	
D16	II	330 kcts/s	685 nm	Center(II-2,II-3): S=1/2	10%			-	130 MHz
				Side(II-1,II-4): S=1	10%			0.89±0.14 GHz	-

Table S1. Summarized information of defect examples. The laser power is set at 15 μW and the microwave power is at 25 dBm. An asterisk (*) indicates that the spin state does not exhibit pulsed ODMR contrast; in such cases, the spin multiplicity is inferred and not experimentally confirmed. A dash (–) denotes parameters that could not be resolved or were not observable.

VIII. Spin defects in $^{12}CO_2$ implanted hBN

As a comparison, we also create spin defects with $^{12}CO_2$ ions using the same procedure as described in Section II. We first perform the PL and ODMR confocal map as presented in Figure S26. In the ODMR contrast maps, we observe non-zero contrasts only when we drive the microwave at 2.01 GHz (71.8 mT), indicating that Group II and Group III defects do not exist in the ^{12}C implanted hBN. Figure S27 present three example ODMR spectra that we typically observe in the $^{12}CO_2$ implanted hBN. We observe both spin S=1/2 and S=1 transitions in single defects at different spots. None of the defects show hyperfine structure due to the lack of ^{13}C atoms.

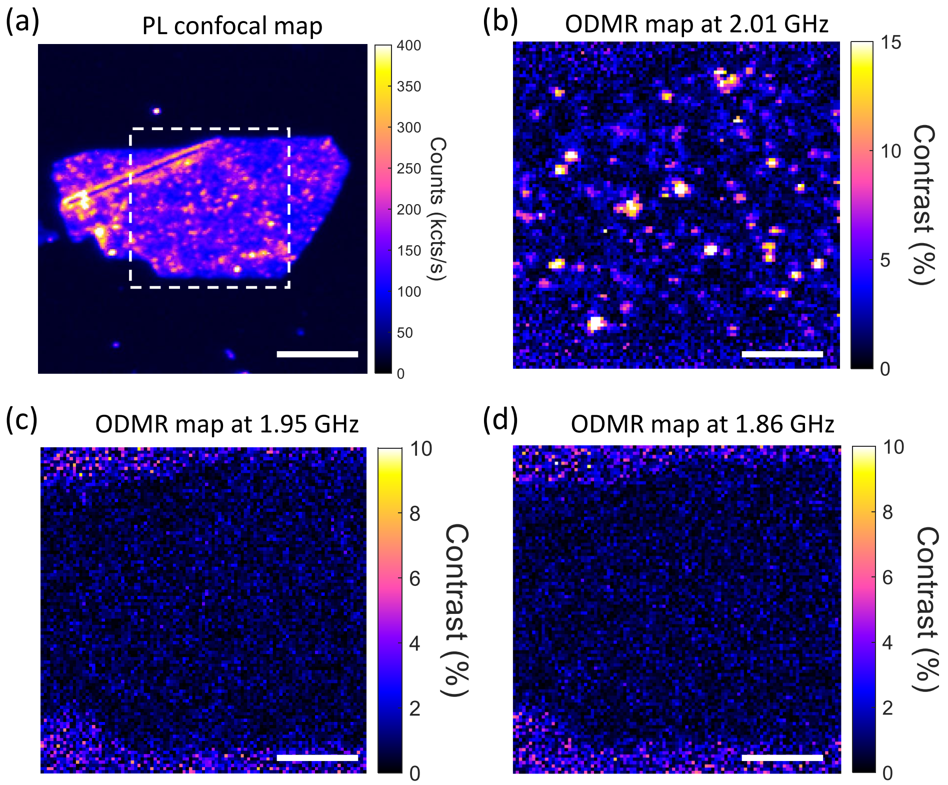

Figure S26. (a) Confocal PL map of a $^{12}CO_2$ implanted hBN after annealing and strong laser exposure. Scale bar is 10 μm. (b)-(d) ODMR contrast mapped by driving the microwave at (b) 2.01 GHz, (c) 1.95 GHz, and (d) 1.86 GHz. The scanned area is marked by the white dashed line (a). A 71.8 mT external magnetic field is applied along the out-of-plane axis. The microwave power is set at 30 mW. Scale bars are 5 μm.

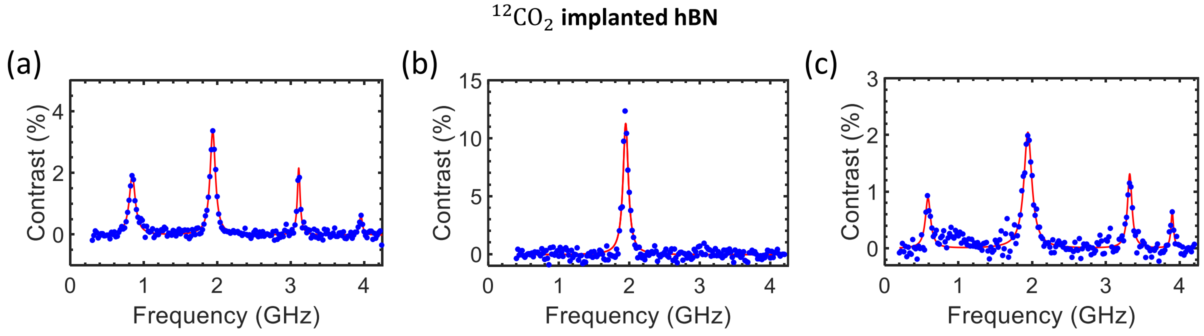

Figure S27. Examples of observed ODMR spectra in $^{12}CO_2$ implanted hBN samples. A 69.3 mT external magnetic is applied.

IX. Spin defects in Helium ion implanted hBN

To better understand the origination of these defects, we further prepare a sample using Helium ion implantation followed by thermal annealing. As shown in Figures S28, S29, we observe spin defects similar to those found in $^{12}CO_2$-implanted hBN. Since carbon is often present as an unintentional impurity during the growth of hBN, these defects may originate from naturally occurring ^{12}C within the material.

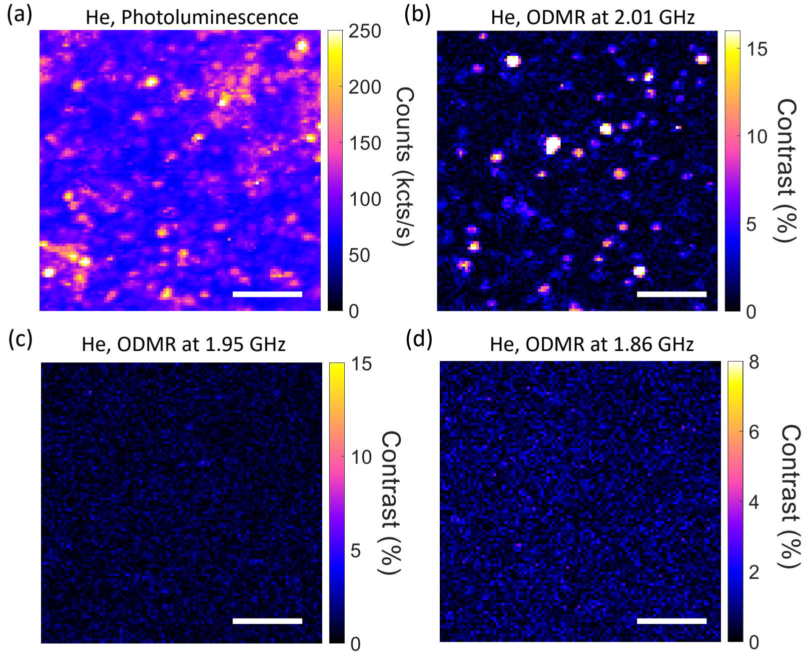

Figure S28. (a) Confocal PL map of a He implanted hBN after annealing and strong laser exposure. (b)-(d) ODMR contrast mapby driving the microwave at (b) 2.01 GHz, (c) 1.95 GHz, and (d) 1.86 GHz. A 71.8 mT external magnetic field is applied along the out-of-plane axis. Scale bars are 5 μm.

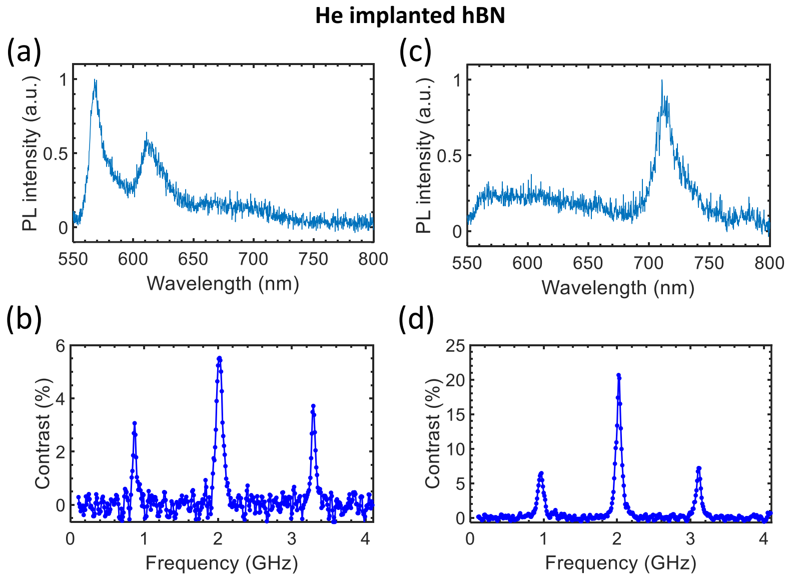

Figure S29. Examples of observed PL spectra and the corresponding ODMR spectra in the He implanted hBN flake. A 71.8 mT external magnetic is applied.

X. Non-implanted hBN with thermal annealing

The helium-implanted samples suggest that intrinsic carbon impurities in hBN can contribute to the formation of spin defects similar to those observed in CO_2 ion-implanted samples. Additionally, we note that as-received hBN without any treatment does not exhibit bright single-photon emitters. To further investigate whether implantation is a necessary step in defect formation, we performed thermal annealing on hBN samples without implantation and characterized the resulting emitters.

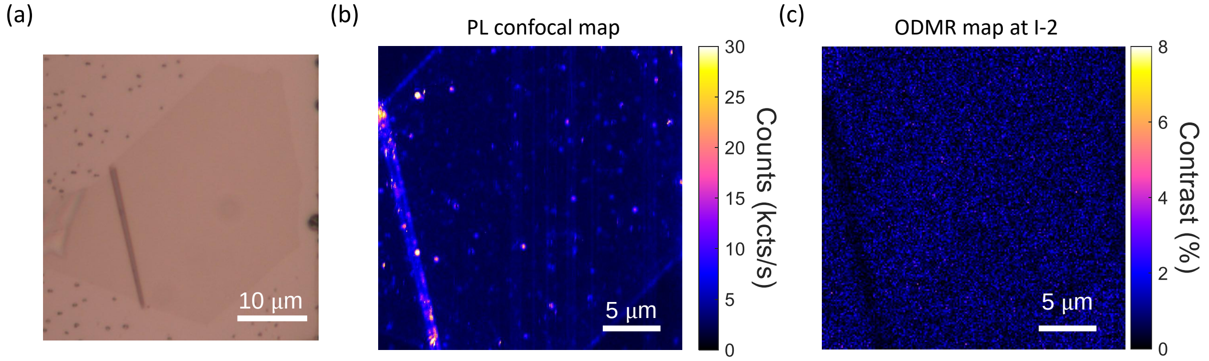

Figure S30. (a) Optical image of an hBN flake without implantation after annealing. Scale bar: 10 μm. (b) PL confocal image of the flake, showing low overall brightness with some emitters appearing near the crack. Scale bar: 5 μm. (c) ODMR map of the same flake, showing no detectable ODMR signals. Scale bar: 5 μm.

As shown in S30 , after annealing, some emitters appear, but their density is significantly lower compared to implanted samples. Furthermore, these emitters predominantly appear near cracks or defect-rich regions in the flakes, while most regions remain dark under PL imaging. The overall brightness of the observed emitters is also notably weaker than those in ion-implanted samples. Most importantly, ODMR measurements reveal that nearly all of these emitters (>99%) do not exhibit detectable spin resonances, making them irrelevant for quantum sensing applications. In our investigation of four different unimplanted, annealed hBN flakes, only one spot exhibited an ODMR signal, but it rapidly quenched within a few minutes.

These findings suggest that ion implantation plays a crucial role in creating a high density of spin-active defects, while thermal annealing alone is insufficient to reliably generate such defects. This can be understood in the context of defect formation under high-temperature annealing. Ion implantation introduces a large number of vacancies, which become mobile at high temperatures and can capture nearby carbon and oxygen impurities, leading to the formation of stable defect complexes. Additionally, during annealing, the vacuum level in our system is maintained at approximately 10^{-5} Torr, but occasionally rises above 10^{-4} Torr when ramping the temperature near 1000 °C due to minor outgassing from system components. This residual gas environment may introduce additional carbon and oxygen impurities, further facilitating defect formation.

In summary, while annealing alone can produce some emitters, its efficiency in generating stable, spin-active defects is very low. The combination of ion implantation and annealing provides a significantly more robust and reproducible approach for spin defect generation in hBN.

XI. DFT calculations of possible defect candidates

To gain more insight into the defect structures, we perform density functional theory (DFT) calculations using Quantum Espresso (QE) [8], an open-source plane-wave software. Both the Perdew-Burke-Ernzerhof (PBE) functional and the Heyd-Scuseria-Ernzerhof (HSE) hybrid functional (the factor of 0.32 for Fock exchange) [9, 10] are employed for the exchange-correlation interaction. We use Optimized Norm-Conserving Vanderbilt (ONCV) pseudopotential [11, 12] for the calculations of excitation energy, and the GIPAW pseudopotential [13] for the calculation of hyperfine interaction parameters and zero-field splitting (ZFS). We set the kinetic energy cutoff to be 55 Ry, which is adequate for converging the relevant properties. Geometry optimizations are carried out with a force threshold of 0.001 Ry/Bohr. We select the $6 \times 6 \times 1$ or higher supercell size of hBN for the calculations of hyperfine parameter and excitation energies. For these calculations, we sample a k-point mesh of $3 \times 3 \times 1$ for the calculation of excitation energies [14], and Γ point for the hyperfine parameters and ZFS [4, 14]. We calculate the zero-phonon line (ZPL) by the constraint

occupation DFT (CDFT) method [15], the hyperfine parameters using the QE-GIPAW code [16], the ZFS by using the ZFS code [14], and we cross compare results between ZFS code and the PyZFS code [17].

Because of the spurious electrostatic interaction between the periodic images containing charged defects and the interaction of charged defect with the homogeneous compensating background charge, it is necessary to calculate the charged cell correction to obtain reliable total energies. The total energy after taking the charged cell correction into account becomes

$$E^q(d) = E^q_{\text{tot}}(d) + E_{\text{corr}}, \tag{13}$$

where $E^q_{\text{tot}}(d)$ is the total energy of the charged defect of charge q, and E_{corr} is the charged cell correction. By using the techniques developed in Refs. [18, 19] and implemented in the JDFTx code [20], we explicitly perform the charge cell correction for the charged defect calculations in this work.

A. Hyperfine Interaction

The hyperfine interaction between nuclear spin and electron spin includes the isotropic (Fermi contact) term at the nucleus site I and the anisotropic dipole-dipole interaction term near the nucleus site I:

$$A_{iso}(I) = \frac{2\mu_0}{3} g_e \mu_e g_I \mu_I \rho_{\text{spin}}(\mathbf{R}) \tag{14}$$

$$A_{aniso}(I) = \frac{\mu_0}{4\pi} g_e \mu_e g_I \mu_I \int d^3r \, \rho_{\text{spin}}(\mathbf{r}) \frac{3\cos^2\theta - 1}{2r^3} \tag{15}$$

where μ_0 is the permeability of vacuum, g_e is the electron g-factor, μ_e is the Bohr magneton, g_I is the g-factor of nucleus I, μ_I is the nuclear magneton, $\mathbf{r}$ is the displacement vector between the electron at $\mathbf{r}_e$ and the nucleus at $\mathbf{R}$, ρ_{spin} is the spin density, and θ is the angle between r and the symmetry axis [21, 22]. The values of g_I used in our calculations are taken from Ref. [23].

B. Zero-Field Splitting

The zero-field splitting is due to the spin-spin interaction. In the first-order, the interaction is described by the following hamiltonian,

$$H_{ss} = \frac{\mu_0}{4\pi} \frac{(g_e \hbar)^2}{r^5} \left[3(\mathbf{s}_1 \cdot \mathbf{r})(\mathbf{s}_2 \cdot \mathbf{r}) - (\mathbf{s}_1 \cdot \mathbf{s}_2) r^2 \right]. \tag{16}$$

Here, μ_0 is the magnetic permeability of vacuum, g_e is the electron g-factor, $\hbar$ is the Planck's constant, $\mathbf{s}_1$ and $\mathbf{s}_2$ denote the spins of the first and second electrons, respectively, and $\mathbf{r}$ is the displacement vector between these two electron. According to the derivation presented in [24], the spin-spin interaction can be reformulated as the product of the ZFS tensor $\mathbf{D}$ and effective total spin $\mathbf{S}$,

$$H = \mathbf{S}^T \mathbf{D} \mathbf{S} \tag{17}$$

where the ZFS tensor contains the interaction details as below,

$$D_{ab} = \frac{1}{2} \frac{\mu_0}{4\pi} (g_e \hbar)^2 \sum_{i>j} \chi_{ij} \langle \Psi_{ij}(\mathbf{r}_1, \mathbf{r}_2) | \frac{\mathbf{r}^2 \delta_{ab} - 3\mathbf{r}_a \mathbf{r}_b}{r^5} | \Psi_{ij}(\mathbf{r}_1, \mathbf{r}_2) \rangle \tag{18}$$

Here, a and b can represent any of the Cartesian coordinates x, y, or z. The indices i and j correspond to all occupied states in both spin-up and spin-down channels. The parameter χ_{ij} is 1 for parallel spins and -1 for anti-parallel spins. The function $\Psi_{ij}(\mathbf{r}_1, \mathbf{r}_2)$ is a two-particle Slater determinant constructed from the Kohn-Sham wavefunctions of the i-th and j-th electronic states. Using the ZFS code and PyZFS [14, 17], we determine the axial and rhombic ZFS parameters, D and E, of the defect candidates in hBN. The eigenvalues of the ZFS tensor are arranged in the descending order as $D_{zz} > D_{yy} > D_{xx}$.

$$D = \frac{3}{2} D_{zz} \tag{19}$$

$$E = (D_{yy} - D_{xx})/2 \tag{20}$$

C. Results and discussion

Previous DFT-based studies mostly relied on comparisons with optical spectra or ODMR linewidth. This can be inconclusive due to the broad variability in emission wavelengths observed across hBN defect emitters. In contrast, our approach focuses on comparing hyperfine interaction parameters derived from first-principles calculations with experimental ODMR and ODNMR data. The use of ^{13}C-enriched samples introduces well-resolved hyperfine splittings, providing more robust and defect-specific spectroscopic fingerprints. This allows us to significantly narrow down the list of plausible defect structures and exclude many configurations that do not match the experimental hyperfine features.

Our experimental results suggest that each defect electron spin in Group II and III couples strongly to one ^{13}C nuclear spin with characteristic hyperfine splittings. This can help identify defect structure, whose hyperfine parameters can be predicted by DFT calculations. Here, we consider six main defect candidates: isolated point defects C_N and C_B, C_BC_N donor-acceptor pairs (DAPs), C_NC_N dimer, C_BO_B dimer and C_BO_N dimer in their neutral (q=0), negative (q=-1) and positive (q=+1) charged states. We first focus on the center branch of spin defects with S=1/2 GSs, as they exhibit clear hyperfine structures. Recently studies suggest the metastable configuration of the S=1/2 spin manifolds, while the optical transitions are supposed to come from the singlet GS to a singlet excited state (ES) (S=0) [25, 26]. Therefore, for the simulation of ODMR using hyperfine tensors, we mainly focus on the S=1/2 spin manifolds. And we compare the optical spectra to the calculated ZPLs in the singlet manifolds.

A previous study investigated the point defects C_B and C_N [7]. The neutrally charged C_B and C_N defects exhibit S = 1/2 states and couple to the carbon nuclear spin with a strengths of 231 MHz and 156 MHz, respectively. C_N^0 defects show a hyperfine value close to that of the Group II defects. However, the ZPL of C_N^0 is predicted to be at 500 nm, which is significantly different from the observed results.

Recent studies also suggest a DAP model to explain the carbon-related defects, and their S=1/2 charged state (q=1) couples strongly to only one carbon nucleus at the C_N lattice site[7]. The C_BC_N-DAP-L defect has a C_BC_N dimer structure but with a larger separation (L lattice distance) between the two point defects. For the neutrally charged C_BC_N-DAP-L, an electron will transfer from C_B towards C_N and form a nonparamagnetic structure ($C_B^+C_N^-$-DAP-L). A positively charged state can be created by removing the excess electron from C_N^-, resulting in a DAP with S=1/2 GSs ($C_B^+C_N^0$-DAP-L). Since the spin density is primarily localized at C_N and is barely affected by the distance L, the hyperfine coupling strengths barely change at different L (Figures S31, S32). The hyperfine coupling strength to the nearest ^{11}B nuclei is about -17.6 MHz (Table S7). Indeed, this hyperfine value qualitatively agrees with the additional ODNMR transition frequency at around 9.2 MHz (Figure S9(b)), which is expected to be half of the hyperfine interaction strength. According to these results, the ODMR spectrum is expected to resolve two hyperfine peaks with a separation of ∼135 MHz (Table S7), which is close to our observations in Group II defects. Additionally, the ODMR linewidth of $C_B^+C_N^0$-DAP-L is calculated to be 74 MHz, matching the natural linewidth of 75 MHz we observed at II-2 and II-3 (Figure S6(d)).

To explain the large hyperfine strength (300 MHz) of the Group III defects, the electronic spin density should be localized at the C_B lattice site since an isolated point defect C_B shows a large hyperfine strength. A positively charged C_BO_N (q=1) defect (Figures S33, S34) with C_{2v} symmetry contains a S=1/2 manifold. The hyperfine strength with the C^{13} nuclear spin is calculated to be A_{zz} = 314 MHz (Table S4), in good agreement with the experimental observation (300 MHz). The linewidth is calculated to be approximately 23 MHz, which is similar to the observed linewidth (25 MHz) at resonance III-2 of Defect 3 using a weak microwave driving. C_BO_N also has a negatively charged state (q=-1) with S=1/2 manifold. The hyperfine strength is 0.1 MHz, which is much smaller than 34 MHz that we observed at III-3. Therefore, the additional peak III-3 is supposed to be a different electronic spin.

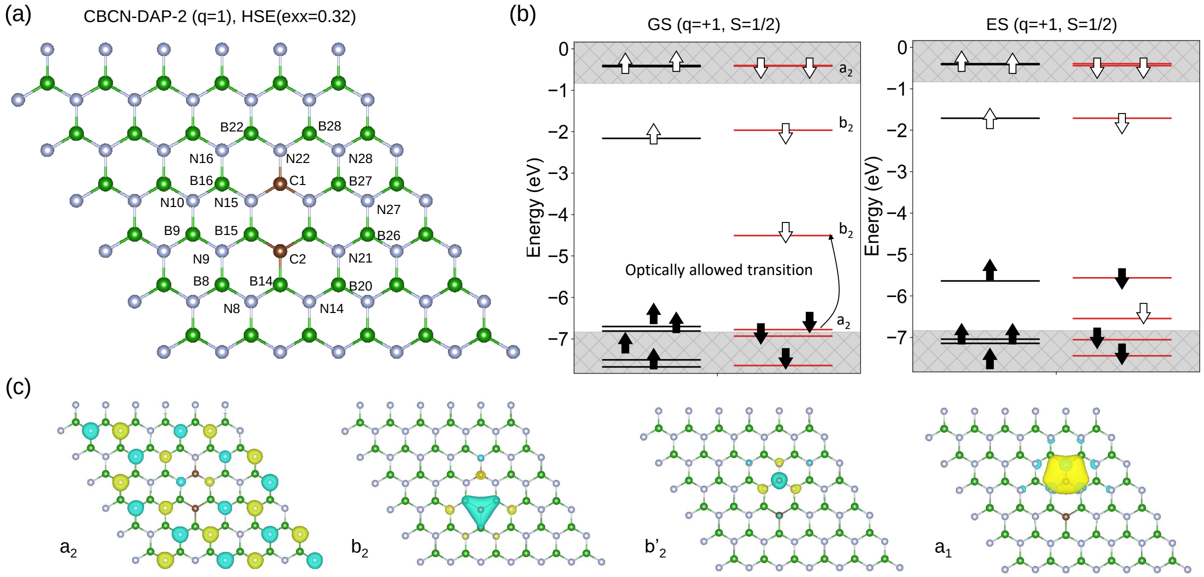

Figure S31. (a) Illustration of a $C_B^+C_N^0$-DAP-2 defect. The labeled atoms are the main contributors to hyperfine interactions. (b) The Kohn-Sham diagram of the $C_B^+C_N^0$-DAP-2 defect at the GS and the ES by CDFT at the HSE level of theory. (c) The corresponding wavefunctions of the single-particle states that are labeled in the Kohn-Sham diagram. a_1, a_2 and b_2 are the irreducible representations of C_{2v} point group. b_2 and b_2' label the different electronic states of the same character. The isosurface is set to 5% of the maximum for a_2, b_2 and b_2'. The isosurface is set to 50% of the maximum for a_1 for better visualization.

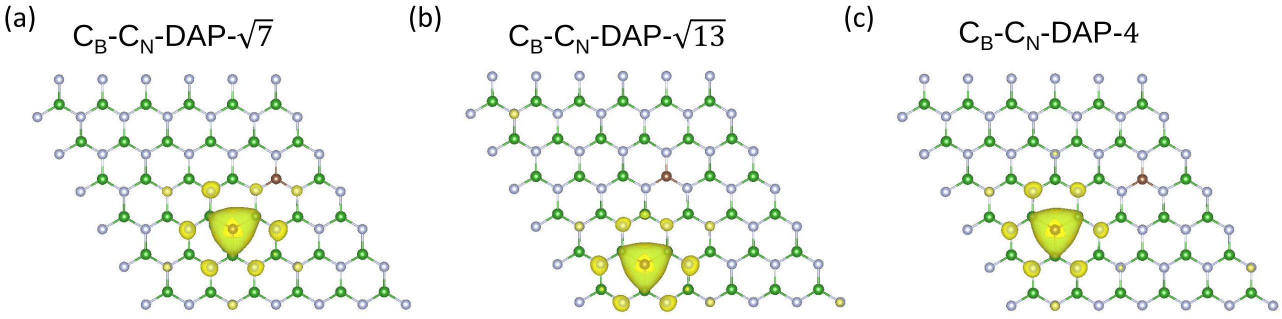

Figure S32. Calculated spin density function for different C_BC_N-DAP-L.

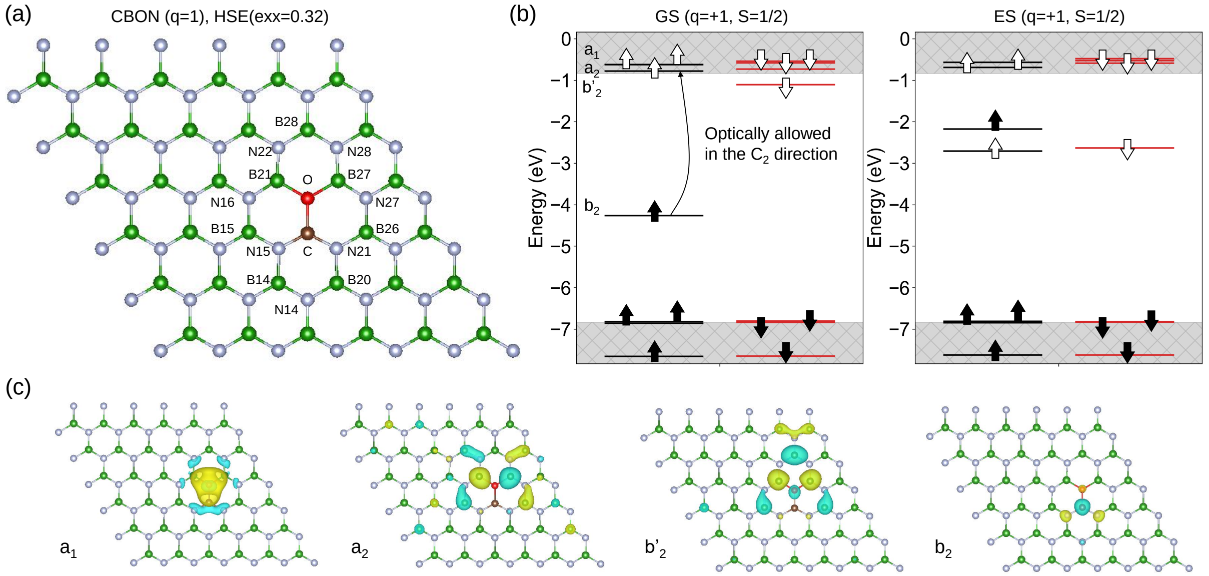

Figure S33. (a) Illustration of a C_BO_N defect. The labeled atoms are the main contributors to hyperfine interactions. (b) The Kohn-Sham diagram of the C_BO_N (q=1) defect at the ground state (GS) and the excited state (ES) by CDFT at the HSE level of theory. (c) The corresponding wavefunctions of the single-particle states that are labeled in the Kohn-Sham diagram. a_1, a_2, b_2 are the irreducible representations of C_{2v} point group, and b_2 and b_2' label the different electronic states of the same character. The isosurface is set to 5% of the maximum.

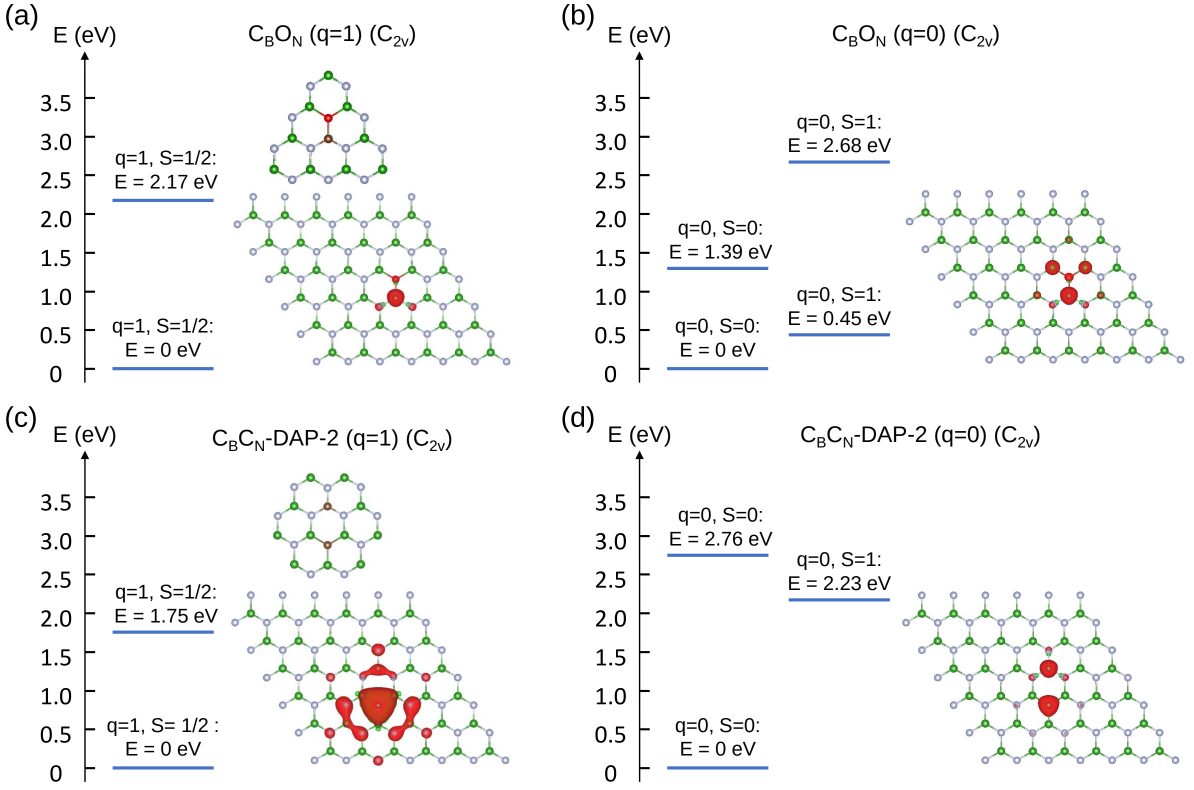

Figure S34. **Summary of the spin defect candidates** Chemical structure, spin density and energy level diagram of (a) the positively charged C_BO_N defect. (b) the neutrally charged C_BO_N defect. (c) the positively charged $C_B^+C_N^0$-DAP-2 defect. (d) the neutrally charged $C_B^+C_N^-$-DAP-2 defect.

Method	Defect	Symmetry	Charge state (q)	ZPL (eV)	Spin, S	GS?	D (MHz)	E (MHz)	A_{zz} (MHz)
HSE	C_BO_N	C_{2v}	0	1.39	0	Yes	-	-	-
HSE	C_BO_N	C_{2v}	0		1	No	1648	534	153
HSE	C_BO_N	C_{2v}	1	2.17	1/2	Yes	-	-	314
HSE	C_BO_N	C_{2v}	-1	0.33	1/2	Yes	-	-	0.1
HSE	C_BC_N-DAP-2	C_{2v}	0	2.76	0	Yes	-	-	-
HSE	C_BC_N-DAP-2	C_{2v}	0		1	No	1542	493	144
HSE	C_BC_N-DAP-2	C_{2v}	1	1.75	1/2	Yes	-	-	166
HSE	C_BC_N-DAP-$\sqrt{7}$	C_1	0	2.14	0	Yes	-	-	-
HSE	C_BC_N-DAP-$\sqrt{7}$	C_1	0		1	No	1805	598	139
HSE	C_BC_N-DAP-$\sqrt{7}$	C_1	1	2.02	1/2	Yes	-	-	173
HSE	C_BC_N-DAP-$\sqrt{13}$	C_1	0	1.84	0	Yes	-	-	-
HSE	C_BC_N-DAP-$\sqrt{13}$	C_1	0		1	No	1565	1038	142
HSE	C_BC_N-DAP-$\sqrt{13}$	C_1	1	2.19	1/2	Yes	-	-	171
HSE	C_BC_N-DAP-4	C_{2v}	0	1.75	0	Yes	-	-	-
HSE	C_BC_N-DAP-4	C_{2v}	0		1	No	1562	1067	141
HSE	C_BC_N-DAP-4	C_{2v}	1	2.23	1/2	Yes	-	-	173

Table S2. Calculated ZPLs, total spins, ZPS parameters (D and E) and longitudinal hyperfine coupling strengths A_{zz} with ^{13}C of several possible defect candidates. The calculations are performed at the HSE (exx=0.32) level of theory.

Method	Defect	Symmetry	Charge state (q)	ZPL (eV)	Spin, S	GS?	D (MHz)	E (MHz)	A_{zz} (MHz)
PBE	C_NC_N	C_{2v}	0	1.42	1	Yes	560	60	75
PBE	C_NC_N	C_{2v}	-1	1.34	1/2	Yes	-	-	70
PBE	C_BO_N	C_{2v}	0	0.59	0	Yes	-	-	-
PBE	C_BO_N	C_{2v}	0		1	No	869	139	120
PBE	C_BO_N	C_{2v}	1	1.75	1/2	Yes	-	-	247
PBE	C_BO_N	C_{2v}	-1	0.21	1/2	Yes	-	-	4
PBE	C_BO_B	C_1	0	1.97	0	Yes	-	-	-
PBE	C_BO_B	C_1	0		1	No	1179	443	
PBE	C_BO_B	C_1	-1	0.22	1/2	Yes	-	-	138
PBE	C_BC_N-DAP-2	C_{2v}	0	2.50	0	Yes	-	-	-
PBE	C_BC_N-DAP-2	C_{2v}	0		1	No	816	25	111
PBE	C_BC_N-DAP-2	C_{2v}	1	1.02	1/2	Yes	-	-	135
PBE	C_BC_N-DAP-$\sqrt{7}$	C_1	0	2.07	0	Yes	-	-	-
PBE	C_BC_N-DAP-$\sqrt{7}$	C_1	0		1	No	1089	117	106
PBE	C_BC_N-DAP-$\sqrt{7}$	C_1	1	1.28	1/2	Yes	-	-	151
PBE	C_BC_N-DAP-$\sqrt{13}$	C_1	0	1.82	0	Yes	-	-	-
PBE	C_BC_N-DAP-$\sqrt{13}$	C_1	0		1	No	960	468	109
PBE	C_BC_N-DAP-$\sqrt{13}$	C_1	1	1.43	1/2	Yes	-	-	153
PBE	C_BC_N-DAP-4	C_{2v}	0	1.75	0	Yes	-	-	-
PBE	C_BC_N-DAP-4	C_{2v}	0		1	No	984	490	108
PBE	C_BC_N-DAP-4	C_{2v}	1	1.49	1/2	Yes	-	-	156

Table S3. Calculated ZPLs, total spins, ZPS parameters (D and E) and longitudinal hyperfine coupling strengths A_{zz} with ^{13}C of several possible defect candidates. The calculations are performed at the PBE level of theory.

Calculated hyperfine tensors of the C_BO_N (q=1) defect at HSE				
Atom	Label	A_{xx} (MHz)	A_{yy} (MHz)	A_{zz} (MHz)
C	C1	53.38	53.07	313.82
B	B15	1.83	-1.32	7.35
B	B26	1.83	-1.32	7.35
B	B21	-1.74	-1.91	0.84
B	B27	-1.74	-1.91	0.84
B	B14	0.32	1.7	-2.34
B	B20	0.32	1.7	-2.34
B	B28	-0.36	-0.39	0.4
N	N15	-8.37	-9.36	2.63
N	N21	-8.37	-9.36	2.63
N	N16	-0.03	-0.47	1.07
N	N27	-0.03	-0.47	1.07
N	N14	0.13	-0.28	2.50
N	N22	-0.18	-0.2	-0.06
N	N28	-0.18	-0.2	-0.06

Table S4. Calculated hyperfine tensors of a positively charged C_BO_N (q=1) defect at HSE (EXX=0.32). Atoms are labeled in Figure S33

Calculated hyperfine tensors of the C_BO_N (q=1) defect at PBE				
Atom	Label	A_{xx} (MHz)	A_{yy} (MHz)	A_{zz} (MHz)
C	C1	9.76	9.22	247.04
B	B15	3.12	0.17	12.15
B	B26	3.12	0.17	12.15
B	B21	-1.45	-1.58	0.81
B	B27	-1.45	-1.58	0.81
B	B14	1.23	-1.17	4.19
B	B20	1.23	-1.17	4.19
B	B28	-0.33	-0.35	0.4
N	N15	-7.61	-8.00	3.05
N	N21	-7.61	-8.00	3.05
N	N16	-0.39	-0.79	1.06
N	N27	-0.39	-0.79	1.06
N	N14	-0.43	-0.82	2.38
N	N22	-0.16	-0.17	-0.03
N	N28	-0.16	-0.17	-0.03

Table S5. Calculated hyperfine tensors of a positively charged C_BO_N (q=1) defect at PBE. Atoms are labeled in Figure S33

Calculated hyperfine tensors of the C_BC_N (q=1) defect at HSE				
Atom	Label	A_{xx} (MHz)	A_{yy} (MHz)	A_{zz} (MHz)
C	C1	0.61	-0.93	4.25
C	C2	-6.05	-6.17	165.72
B	B14	-18.87	-18.42	-23.76
B	B15	-18.69	-15.21	-24.68
B	B21	-18.69	-15.21	-24.68
B	B8	-1.35	-0.87	-2.70
B	B20	-1.35	-0.87	-2.70
B	B9	-2.37	-2.64	-1.22
B	B26	-2.37	-2.64	-1.22
B	B16	-1.03	-1.41	-0.18
B	B27	-1.03	-1.41	-0.18
B	B22	-0.71	-0.73	-0.17
B	B28	-0.71	-0.73	-0.17
N	N22	0.09	-0.08	2.53
N	N15	-0.05	0.10	-0.63
N	N21	-0.05	0.10	-0.63
N	N16	-0.08	-0.09	0.03
N	N28	-0.08	-0.09	0.03
N	N10	0.15	-0.02	0.88
N	N27	0.15	-0.02	0.88
N	N9	0.11	-0.52	6.82
N	N20	0.11	-0.52	6.82
N	N8	0.03	-0.59	3.03
N	N14	0.03	-0.59	3.03

Table S6. Calculated hyperfine tensors of a positively charged C_BC_N (q=1) defect at HSE (EXX=0.32). Atoms are labeled in Figure S31.

Calculated hyperfine tensors of the C_BC_N (q=1) defect at PBE				
Atom	Label	A_{xx} (MHz)	A_{yy} (MHz)	A_{zz} (MHz)
C	C1	-0.06	-1.35	3.92
C	C2	-7.45	-7.55	135.31
B	B14	-14.01	-12.76	-17.74
B	B15	-12.31	-9.25	-17.62
B	B21	-12.31	-9.25	-17.62
B	B8	-1.39	-0.65	-2.42
B	B20	-1.39	-0.65	-2.42
B	B9	-2.21	-2.46	-1.10
B	B26	-2.21	-2.46	-1.10
B	B16	-1.08	-0.49	-1.70
B	B27	-1.08	-0.49	-1.70
B	B22	-0.92	-1.17	-0.38
B	B28	-0.92	-1.17	-0.38
N	N22	-0.39	-0.54	3.35
N	N15	-0.17	-0.61	0.43
N	N21	-0.17	-0.61	0.43
N	N16	0.01	-0.07	0.12
N	N28	0.01	-0.07	0.12
N	N10	-0.14	-0.029	1.82
N	N27	-0.14	-0.029	1.82
N	N9	-1.10	-1.59	7.02
N	N20	-1.10	-1.59	7.02
N	N8	-0.62	-1.05	3.47
N	N14	-0.62	-1.05	3.47

Table S7. Calculated hyperfine tensors of a positively charged C_BC_N (q=1) defect at PBE. Atoms are labeled in Figure S31.

XII. Modeling spin dynamics: spin pair, charge state conversion and energy levels

In the preceding sections, we characterized the key properties of three distinct types of spin defects. Groups II and III defects were identified as arising from ^{13}C substitution introduced via ion implantation. Although Group I defects lack hyperfine structure, some may share similar chemical and electronic structures to those in Groups II and III, with the primary distinction being the presence of intrinsic ^{12}C substitution instead of implanted ^{13}C.

First-principles DFT calculations provide insight into possible defect chemical structures and show good agreement with our ODMR observations. However, these calculations alone cannot fully account for the origin of multiple spin transitions observed within a single photon emitter. To further understand the nature of these defects, we turn our attention to several key features revealed in the ODMR measurements, which offer valuable clues into their electronic structure and spin dynamics:

(1) **Multiple ODMR Peaks:** The presence of multiple peaks in ODMR spectra, corresponding to two distinct spin numbers S within a single defect, suggests a significant role of charge states in determining their spin dynamics.

(2) **Asymmetric Rabi Oscillations:** Some Rabi measurements reveal clear asymmetry and beating patterns. The beating signal in Rabi oscillations points to an underlying spin-pair model for these defects. And the defect with highly asymmetric Rabi oscillation likely host a metastable spin manifold.

(3) **Correlation Between Spin Manifolds:** The S=1/2 ODMR contrast in Group III defects decreases significantly as the S = 1 transition approaches the LAC. Similarly, the contrast of both S = 1 and S= 1/2 transitions vanishes or falls below the detection limit at zero magnetic field (at the LAC of the S = 1/2 manifold). These behaviors suggest a strong correlation between the two spin manifolds, likely involving direct state transitions or coupling between them.

(4) **Nuclear spin polarization at the S = 1 LAC:** At the LAC of the S = 1 manifold, the hyperfine structure asymmetry in the S=1/2 ODMR suggests that both spin manifolds couple to the same nuclear spin for some defects in Group III (Defect 3 for example).

Connecting these observations across defect groups enhances our understanding of their electronic structures and spin dynamics. In this section, we delve deeper into the origins of the multiple ODMR peaks and explore the physical mechanisms underlying these observations.

Recent studies propose a spin-pair model to explain the finite ODMR contrast observed in S = 1/2 spin defects[25]. According to this model, each optically active spin color center comprises two electrons localized at separate point defects a few nanometers apart (Figure S35(a)). These defects, forming a donor-acceptor pair, exhibit different electronic structures. One electron can transfer between the two defects without altering the total charge of the defect pair. When the electrons occupy separate defects (labeled A and B), they form a weakly coupled S = 1/2 spin pair (Figure S35(b), right panel). Conversely, when both electrons reside at a single defect (defect A), they form either a singlet or triplet state (S = 0 or 1). Laser pumping can induce charge transfer between these two states.

The actual GS, which is also the optically active state, is determined by the lowest-energy charge state and depends on the specific defect species and the Fermi level. Consequently, two possible energy level configurations arise, as illustrated in Figure S35 (c) and (d).

In Model 1 as shown in Figure S35(c), the GS is a singlet (S = 0), where both electrons occupy Defect A. Following optical excitation, the electron may decay radiatively or via a S = 1 metastable state through non-radiative intersystem crossing (ISC). The latter one leads to the observed S = 1 resonances. Additionally, one electron may hop to Defect B, forming a metastable S = 1/2 spin pair state. This charge transfer process is significantly accelerated up under laser excitation [26]. The energy levels of the spin pair manifold are labeled as $T_{\pm}$, T_0 and S, with the corresponding transitions giving rise to the observed S = 1/2 ODMR peaks.

In Model 2 as shown in Figure S35(d), the spin pair manifold resides in the GS and has an optically active ES (Figure S35(d)). Laser excitation can induce charge transfer to form the strongly coupled spin states as illustrated by the blue arrows.

Here, we note that in both cases, we place the S = 1 manifold in the metastable state based on DFT calculations. However, while the possibility of an S = 1 ground-state manifold is low, it cannot be fully excluded. In the following sections, we validate the spin-pair model and investigate these two electronic structures through numerical simulations.

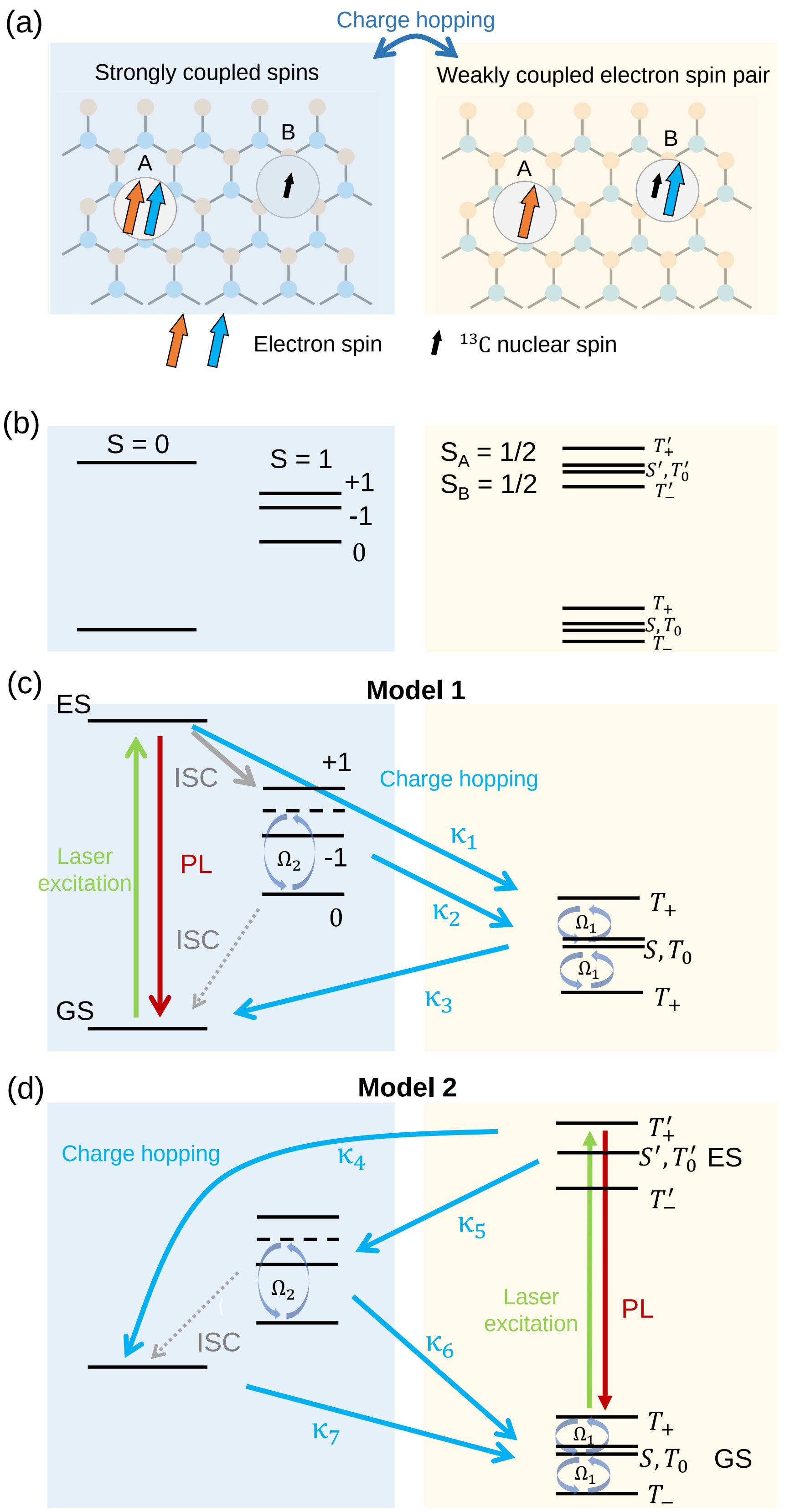

Figure S35. (a) Two possible electron configurations of a single spin color center in different internal charge states. (b) The corresponding energy structures when the defect is in different internal charge states. The lowest energy level among these states depends on the nature of the two defects. (c)-(d) Energy level diagrams and transition rates between states, considering two different models.

A. Asymmetry and Beating in Rabi oscillation

Asymmetry in Rabi Experimentally, we observed that some defects exhibit highly asymmetric Rabi oscillations, where the contrast continuously increases as the microwave pulse duration is ramped up (examples in Figure S24). This phenomenon typically indicates the presence of a long-lived metastable state with a lifetime comparable to the microwave pulse duration[27]. In such cases, a finite population from the metastable state transfers back to the GS (optical manifold) during the dark time, leading to an overall increase in the defect's brightness.

In Rabi measurements, the contrast is determined by comparing the PL counts with and without the microwave pulse. For both cases, the initialization and readout laser pulses are separated by the same duration, which remains fixed for different microwave pulse durations. This setup eliminates the influence of spin-independent transitions between the MS and GS on the Rabi contrast. Consequently, the observation of highly asymmetric Rabi oscillations suggests that these defects likely host a metastable S = 1/2 manifold.

However, defects exhibiting only subtle asymmetry in their Rabi oscillations are also observed, particularly in Group III. These defects also exhibit relatively long T_1 relaxation times, most exceeding 40 μs and many surpassing 100 μs. To further investigate this behavior, we performed two types of pulsed measurements (for the S = 1/2 manifold), as shown in Figure S36. These sequences are commonly used to determine the spin relaxation time. However, if the spin manifold resides in a metastable state, the measured decay time is also influenced by transition rates from the MS to the GS.

In these measurements, the microwave π pulse is placed either at the end (Sequence 1) or at the beginning (Sequence 2) of the dark time. For a GS spin configuration, we expect both sequences to yield similar decay times. However, the situation will be different for an MS spin configuration. Because the positive contrast indicates that the laser pulse initializes the MS into a long-lived state, a microwave π pulse will flip the spin into a short-lived state. Consequently, if the π pulse is applied at the beginning of the dark period, we expect to observe a shorter decay time than when the π pulse is applied at the end, particularly for defects exhibiting high Rabi asymmetry.

For Group III defects, we find that the T_1 relaxation time and contrast remain nearly identical, regardless of whether the π pulse is applied at the beginning or end of the dark interval (Figure S36(c)). This suggests that a GS spin-1/2 manifold (Model 2) is possible. In contrast, the MS spin-1/2 manifold (Model 1) appears more common, as T_1 measurements typically yield a shorter decay time when the π pulse is applied at the beginning of the dark time—an effect frequently observed in many Group I defects with high Rabi asymmetry (Figure S36(b)).

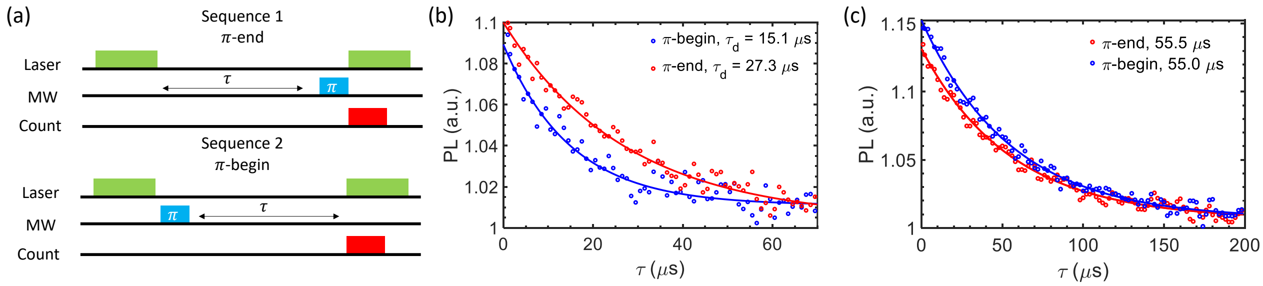

Figure S36. (a) Illustration of the two pulse sequences used for relaxation measurements. For Sequence 1, the π pulse is applied at the end of the dark time. For Sequence 2, π pulse is applied at the start of the dark time. The reference count is taken with the same sequence but without a microwave pulse. (b) An example of relaxation measurements using a Group I defect, where two sequences show significant difference in the decay time: $T_{1,end} = 27.3 \pm 3.9$ μs, and $T_{1,begin} = 15.1 \pm 2.4$ μs. (c) An example of relaxation measurements using a Group III defect, where two sequences show nearly the same decay time: $T_{1,end}$ $= 55.0 \pm 3.2$ μs, and $T_{1,begin} = 55.5 \pm 3.7$ μs.

Beating patterns in Rabi One crucial piece of evidence supporting the spin-pair model is the characteristic beating pattern in Rabi oscillations. This phenomenon is observed in Figure S24 for Group I defects. To illustrate this further, Figure S37 presents three examples of Rabi measurements on a Group I defect at different microwave powers. At relatively low microwave power, the Rabi curve shows multiple superimposed oscillation components. Fast Fourier Transform (FFT) analysis reveals two distinct frequency components, with one approximately double the other. The lower frequency corresponds to the expected standard Rabi oscillation frequency. As the microwave power increases, the double-frequency component becomes more pronounced, ultimately dominating the oscillation at 35 dBm (Fig. S37(c),(f)), where the standard Rabi component is nearly suppressed.

Theoretical Modeling To explain this phenomenon, we use the quantum master equation to simulate the Rabi oscillations based on a simplified energy level structure, focusing on the spin-1/2 transitions. We use a simplified

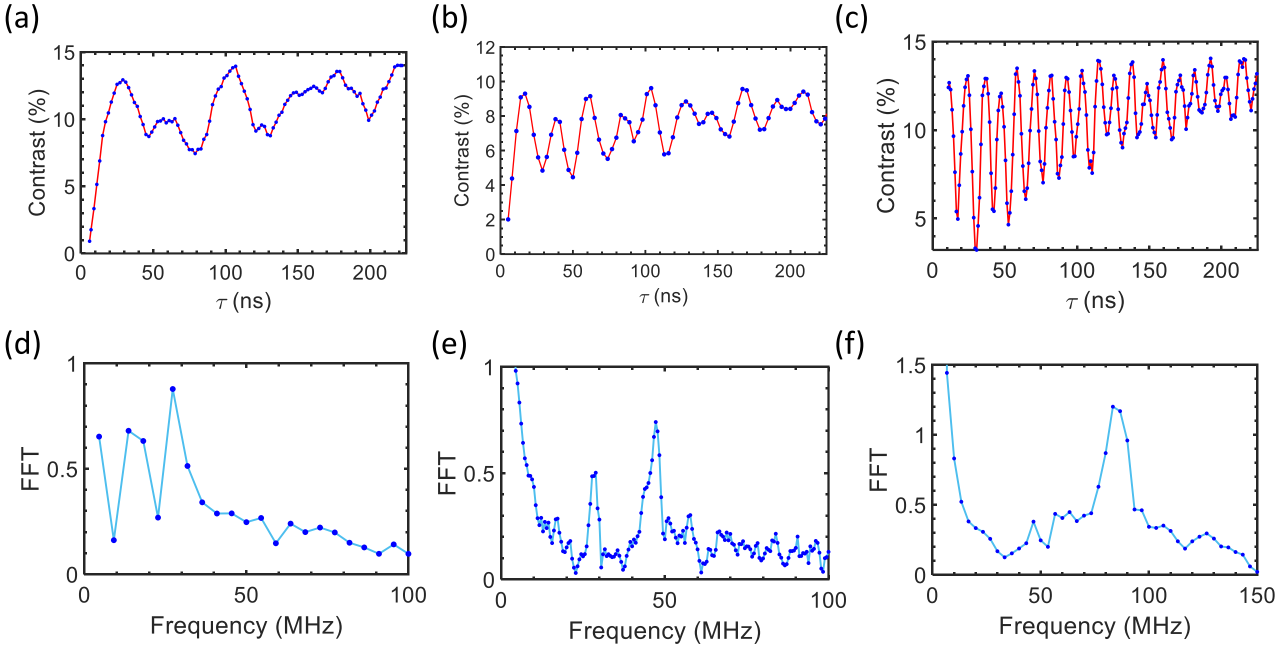

Figure S37. (a)–(c) Rabi oscillations of a Group I defect at the I-2 transition (S=1/2) under microwave driving powers of (a) 22 dBm, (b) 29 dBm, and (c) 35 dBm. (d)–(f) Fast Fourier transforms of the corresponding Rabi oscillations in (a)–(c), respectively.

model including a singlet GS $|g\rangle$ and a spin-pair metastable-state manifold (Figure S38(a)). Spin-dependent charge hopping enables transitions from the spin-pair manifold to the singlet ground state, dictated by selection rules with different rates, $|T\rangle$ and $|ST_0\rangle$. Electrons in the spin-pair state relax to the GS $|g\rangle$ with rates Γ_T^g and Γ_S^g, respectively. These varying transition rates provide insight into the spin-1/2 component observed in the ODMR signal.

Using the density matrix formalism, the system dynamics are described by the Lindblad master equation[28]:

$$\dot{\rho} = -i[H, \rho(t)] + \sum_k \Gamma_k \left[L_k \rho(t) L_k^\dagger - \frac{1}{2} \left\{ L_k^\dagger L_k, \rho(t) \right\} \right], \tag{21}$$

where $\rho(t)$ is the time-dependent density matrix, Γ_k represents transition rates, and L_k are the associated Lindblad operators. The first term describes the coherent evolution, while the second accounts for incoherent processes like electronic state transitions and spin dephasing.

The system Hamiltonian consists of two independent components: the GS manifold (H_g) and the spin-pair manifold (H_{pair}), connected by incoherent transitions:

$$H = H_{pair} \oplus H_g = \begin{pmatrix} H_{pair}^{4\times4} & \\ & H_g^{1\times1} \end{pmatrix} \tag{22}$$

The spin-pair Hamiltonian in the rotating wave approximation (RWA) is expressed as:

$$H_{pair} = H_{sA} + H_{sB} + H_{coupling} + H_{disorder} + H_d, \tag{23}$$

where $H_{sA,sB} = \left(\frac{g\mu_B B_0}{\hbar} - \omega_\mu \right) S_{zA,B} = \Delta S_{zA,B}$ represents the Hamiltonian of the individual spins. Here, B_0 is the external static magnetic field, ω_μ is the microwave frequency, and Δ is the detuning frequency between the driving microwave field and the resonant transition frequency. The spin-spin coupling, represented by $H_{coupling}$, is assumed to be weak enough. In this case, we consider only the coupling along z-direction between the two spins: $H_{coupling} = JS_{zA}S_{zB}$, where the coupling constant J depends on the distance between the two spins. Additionally, a microwave driving term, $H_d = \Omega_1(S_{xA} + S_{xB})$, is applied to coherently coupling the spin levels with a Rabi frequency Ω_1. When the two spins are in different local environments such as nuclear spins, environmental variations cause random shifts, ϵ_A and ϵ_B, from the resonant frequency, leading to detuning of the energy levels. The disorder

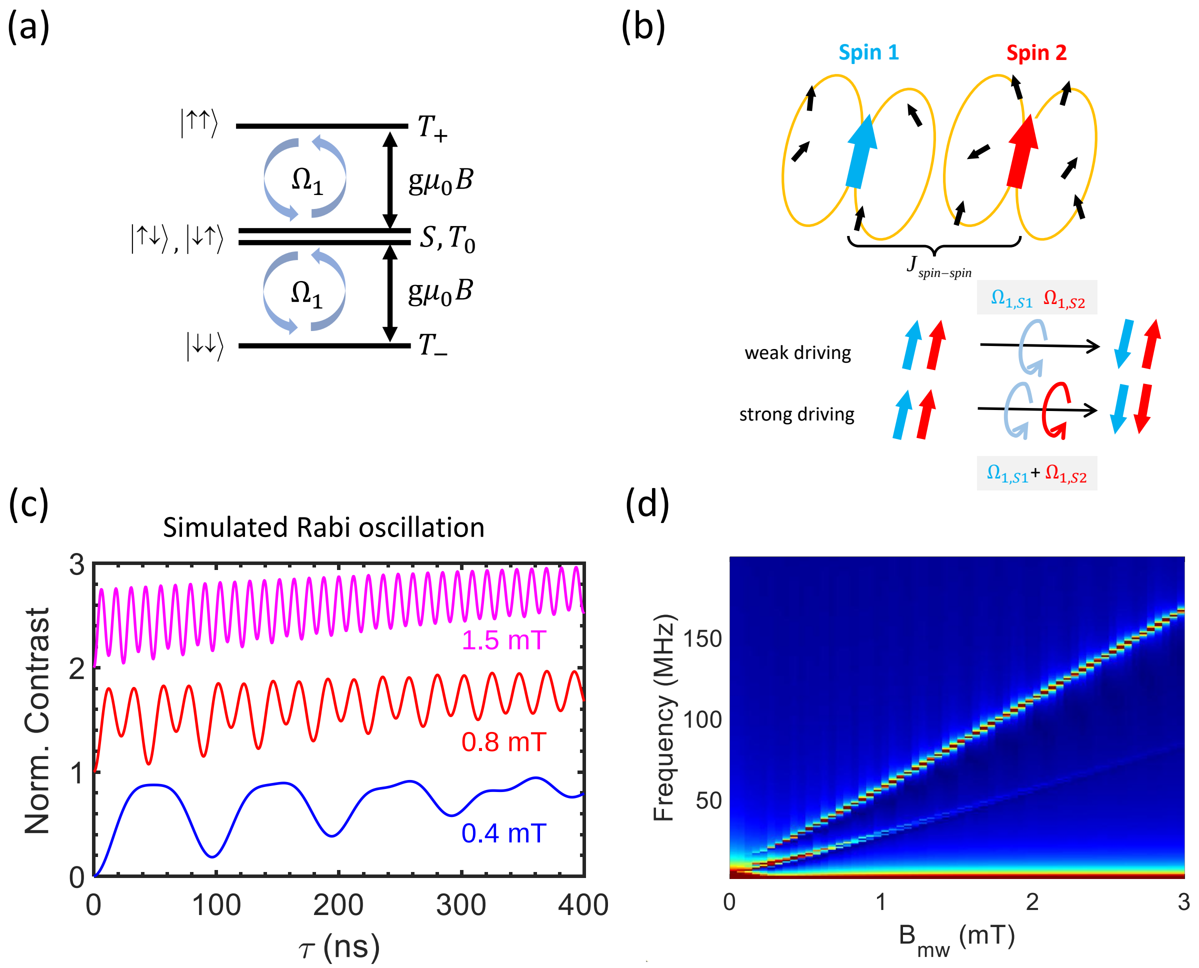

Figure S38. (a) Energy level diagram for Rabi simulation. (b) An illustration of the spin pair model in Rabi measurements. (c) Simulated Rabi oscillations at different microwave driving powers. (d) FFT of the simulated Rabi oscillation at different microwave fields.

Hamiltonian is $H_{disorder} = \epsilon_A S_{zA} + \epsilon_B S_{zB}$. To incorporate incoherent processes, Lindblad operators are introduced for each transition, as shown in Table S8.

incoherent interactions	transition rates	Value $(\Gamma_i/2\pi)$	lindblad operators
Charge hopping from $\lvert T_\pm\rangle$ to $\lvert g\rangle$	Γ_g^T	95 kHz	$L_g^T = \lvert g\rangle\langle T_\pm\rvert$
Charge hopping from $\lvert ST_0\rangle$ to $\lvert g\rangle$	Γ_g^S	38 kHz	$L_g^S = \lvert g\rangle\langle ST_0\rvert$
spin dephasing in S = 1/2 spin pair	γ_ϕ^{sp}	475 kHz, 895 kHz	$\Gamma_{\phi,i} = S_{z,i}$ for i ∈ {A, B}

Table S8. Construct the Lindblad operators for each of the above incoherent interactions for Rabi simulation.

Using the theoretical framework outlined above, the lindblad equation is solved using the Liouville superoperator, $\mathcal{L}$, by calculating the matrix exponential:

$$\rho(t) = exp[\mathcal{L}(t - t_0)]\rho(t_0) \tag{24}$$

From the density matrix, we can obtain the simulated PL signal by assigning different brightness values to the corresponding spin states

$$I_{PL} = \alpha_0\rho_g + \alpha_1\rho_{ST_0} + \alpha_2\rho_{T_\pm}, \tag{25}$$

where $\rho_i, i \in \{g, ST_0, T_\pm\}$ is the population of state $|i\rangle$, derived from the diagonal elements of the density matrix. The

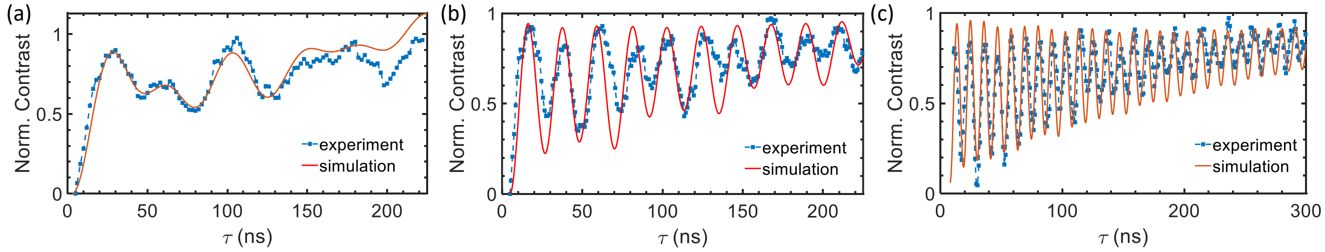

Figure S39. Simulated Rabi oscillation compared with experimental results.

coefficients α_i correspond to the PL intensity of these states. Based on selection rules, we assume that the brightness of the $|T_+\rangle$ and $|T_-\rangle$ ($|T_0\rangle$ and $|S\rangle$) states is identical, a reasonable approximation.

Under ideal readout conditions, the contrast should depend only on the population of the GS. However, in our calculations, the contrast also incorporates contributions from the metastable state populations. This is because, if the laser can read out in an extremely short time, the contrast would only depend on the GS populations. But when the readout time and transition rate are of comparable timescales, population cycling occurs during measurement. Consequently, the metastable population will influence the PL signal, i.e., $\alpha_{1,2} \neq 0$.

Figure S38(c)-(d) presents the simulated Rabi results within the spin pair charge state manifold. As the microwave pulse length increases, the spin pair exhibits clear Rabi oscillations. In addition to the sinusoidal pattern, a beating effect is evident, especially at relatively weak microwave drive. The FFT analysis of the simulated Rabi signal reveals two peaks, with one frequency being approximately twice that of the other.

This power-dependent beating behavior arises from environmental disorder, which affects the two spins in the pair differently. Specifically, when the driving field of B_1 is smaller than the disorder-induced field $|\Delta B_{hyp}|$, the Rabi oscillations predominantly drive only one spin in the pair (S38(b)). Assuming the spin was initialized to $|\uparrow\uparrow\rangle$ state after the first laser pulse, the spin under microwave driving evolve as:

$$|\uparrow\rangle\,|\uparrow\rangle \rightarrow |\uparrow\rangle\,\frac{|\uparrow\rangle + |\downarrow\rangle}{2} \rightarrow |\uparrow\rangle\,|\downarrow\rangle \rightarrow |\uparrow\rangle\,\frac{|\uparrow\rangle - |\downarrow\rangle}{2} \rightarrow |\uparrow\rangle\,|\uparrow\rangle\,. \tag{26}$$

When B_1 increases to a value comparable to $|\Delta B_{hyp}|$, the microwave can be in resonance with both spins and thus drive them simultaneously. In this case, the spins partially evolve as:

$$|\uparrow\rangle\,|\uparrow\rangle \rightarrow \frac{|\uparrow\rangle + |\downarrow\rangle}{2}\,\frac{|\uparrow\rangle + |\downarrow\rangle}{2} \rightarrow |\downarrow\rangle\,|\downarrow\rangle \rightarrow \frac{|\uparrow\rangle - |\downarrow\rangle}{2}\,\frac{|\uparrow\rangle - |\downarrow\rangle}{2} \rightarrow |\uparrow\rangle\,|\uparrow\rangle\,. \tag{27}$$

Because the PL signal depends on the total spin state of the spin pair, both $|\uparrow\rangle\,|\uparrow\rangle$ and $|\downarrow\rangle\,|\downarrow\rangle$ exhibit similar brightness. Consequently, after half a cycle, the PL intensity returns to its original brightness, giving rise to the observed double-frequency components. At even higher microwave power, the driving field overcomes the difference in Larmor frequencies caused by the disorder. At this stage, the $\Omega_1 + \Omega_2$ component becomes dominant, and the $\Omega_1(\Omega_2)$ component is significantly suppressed, where Ω_1 and Ω_2 are the Rabi frequency of the individual electron spin in the pair.

Figure S38(d) shows the Fourier frequency components as a function of the driving field B_1. As expected from the Rabi frequency equation, both frequency peaks scale linearly with B_1. Additionally, the ratio of the peak magnitudes changes with B_1 varies, with the beating signal weakening at lower driving fields.

We further refine our simulation by adjusting the disorder strength to 3.15 MHz and the spin-spin coupling strength to 14.5 MHz. Under these parameters, the simulated Rabi oscillations closely match the experimentally observed data, as shown in Figure S39. The agreement between simulation and experiment reinforces the validity of the spin-pair model in describing the observed beating pattern and asymmetry in Rabi oscillations. This consistency further supports the hypothesis that environmental disorder plays a crucial role in differentiating the two spins within the pair in a Group I defect, leading to distinct oscillation frequencies that merge as the microwave driving power increases.

Rabi oscillations in the presence of strong hyperfine interaction The irregular beating pattern observed in Figure S39 arises from environmental differences between the two spins, leading to distinct hyperfine interactions. These hyperfine variations, comparable to the microwave broadening, result in both spins being driven simultaneously but at slightly different Rabi frequencies. This effect complicates high-fidelity spin control of individual spins. In principle, reducing the microwave driving field could mitigate simultaneous driving. However, this approach is limited by spin decoherence, which sets a lower bound on the microwave power that can be effectively used.

A more robust solution emerges when one of the electron spins is strongly coupled to a single nuclear spin, while the other electron spin remains weakly coupled to the surrounding nuclear environment, a scenario observed in Group

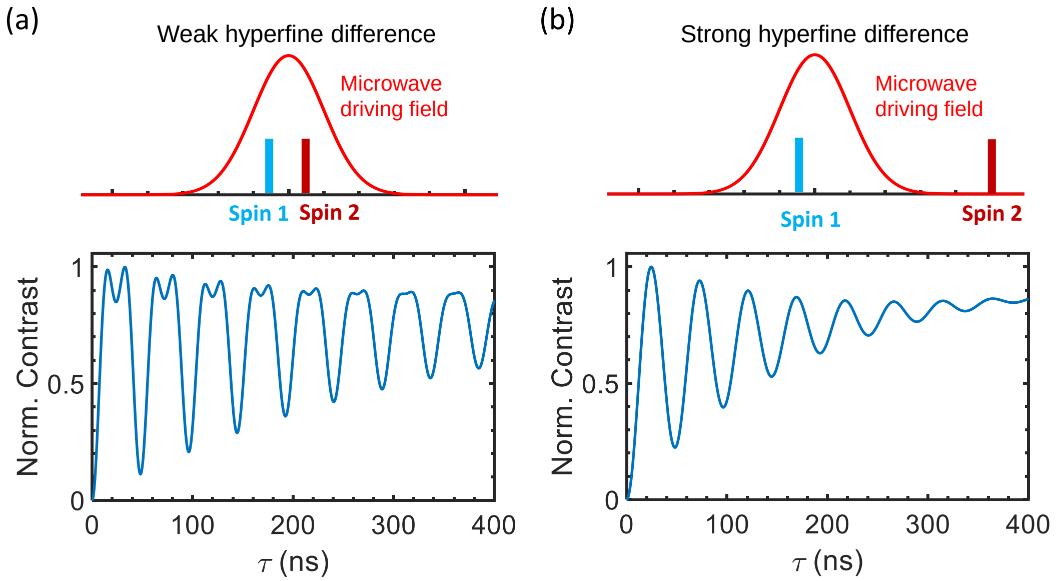

Figure S40. Simulated Rabi oscillation of the spin pair when (a) two spins are near degenerate, (b) one of the spins strongly couples to a nuclear spin ($A_{zz} \gg \Omega_1$).

II and III defects. In this case, the resonance frequencies of the two electron spins become significantly separated, far exceeding the microwave broadening. As a result, selective driving of individual spins becomes feasible, allowing for well-defined microwave control without interference from the second spin.

Figure S40 illustrates simulated Rabi oscillations in two scenarios: (a) when the two spins are nearly degenerate, leading to strong beating effects, and (b) when one spin is strongly coupled to a nuclear spin ($A_{zz} \gg \Omega_1$), effectively isolating the spin response and enabling independent control. These results highlight the advantage of strong hyperfine coupling in achieving precise spin manipulation within spin-pair systems.

B. Modeling B-field dependent ODMR spectra

In the Rabi simulation, we used a simplified model that excluded the spin-1 manifold, assuming the metastable nature of the spin-pair manifold. This assumption is reasonable since the microwave driving predominantly acts within the spin-pair manifold, which is less influenced by other electronic states.

To develop a more comprehensive understanding of the electronic structure, we now analyze the ODMR spectra and their dependence on an external magnetic field. A key feature observed is that the ODMR contrast of the S = 1/2 transition decreases near the LAC of the S = 1 transition. Similarly, a reduction in overall contrast is observed near zero magnetic field, corresponding to the LAC of the S = 1/2 manifold. This behavior suggests a direct coupling between the S = 1/2 and S = 1 manifolds, where state mixing in one manifold influences the other.

To investigate this effect quantitatively, we numerically simulate CW ODMR using two different models: one in which the ground state is a singlet charge state (Model 1) and another in which the ground state is a weakly coupled S = 1/2 spin-pair charge state (Model 2).

1. Model 1: Singlet GS, metastable S = 1 states, and metastable spin pair

In the first model, illustrated in Figure S35(c), the optical manifold consists of a singlet GS and a singlet ES. Within the same internal charge state, the defect hosts a metastable spin S = 1 state, which is connected to the singlet manifold via intersystem crossing (ISC) transitions. Under laser excitation, spin-dependent charge hopping can alter the internal charge state, leading to the formation of a metastable spin-pair state in which two electrons are separated and weakly coupled.

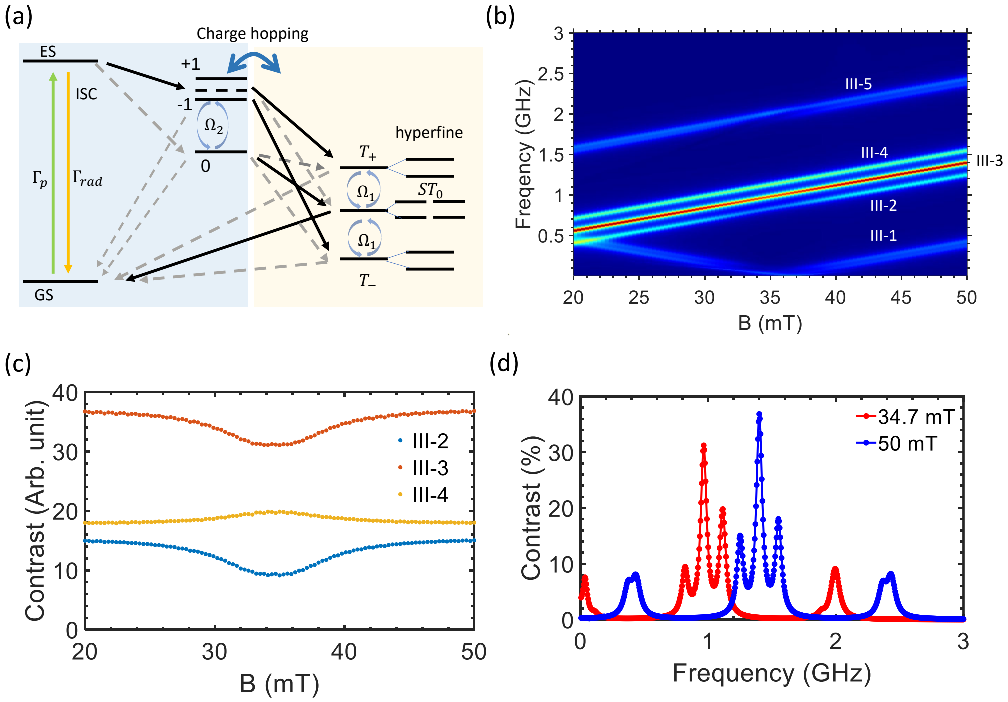

Figure S41. (a) Energy level diagram of model 1 for simulation. The dashed arrows indicates a weak transition compared to the solid arrows. (b) Simulated ODMR as a function of B field. (c) Simulated ODMR contrast of III-3 as a function of B field. The small variation is due to the finite frequency resolution in the simulation. (d) Two ODMR examples considering the magnetic field at LAC (34.7 mT) and far from LAC (50 mT).

The system Hamiltonian is given by:

$$H = H_{pair} \oplus H_{m,S1} \oplus H_{eg} = \begin{pmatrix} H_{pair}^{8\times 8} & & \\ & H_{m,S1}^{6\times 6} & \\ & & H_{eg}^{4\times 4} \end{pmatrix} \tag{28}$$

where H_{eg} and H_{pair} describe the optical manifold and spin-pair states. $H_{m,S1}$ represents the spin S=1 metastable state Hamiltonian. Each Hamiltonian includes a ^{13}C nuclear spin coupled to the electron spin. In the weakly coupled spin-pair state, this nuclear spin strongly interacts with only one of the electron spins, and the Hamiltonian H_{pair} can be written as

$$H_{pair} = \gamma_e \mathbf{B}\cdot\mathbf{S}_A + \gamma_e \mathbf{B}\cdot\mathbf{S}_B + \mathbf{S}_A\mathbf{J}\mathbf{S}_B + \mathbf{S}_A\mathbf{A}\mathbf{I} + \gamma_n \mathbf{B}\cdot\mathbf{I}, \tag{29}$$

where $\mathbf{S}_i$ (i = A,B) and $\mathbf{I}$ represent the S = 1/2 electron spins (A and B) and I = 1/2 nuclear spin, respectively. $\mathbf{J}$ is the coupling strength between two electron spins, and $\mathbf{A}$ is the hyperfine tensor between the electron spin A and the ^{13}C nuclear spin. In the S = 1 manifold, the Hamiltonian is written as:

$$H_{S_1} = DS_{1,z}^2 + E(S_{1,x}^2 - S_{1,y}^2) + \gamma_e \mathbf{B}\cdot\mathbf{S}_1 + \mathbf{S}_1\mathbf{A}\mathbf{I} + \gamma_n \mathbf{B}\cdot\mathbf{I}, \tag{30}$$

where $\mathbf{S}_1$ is the spin S = 1 operator. D and E are the ZFS parameters. We ignore the coherent dynamics within the singlet manifold, and thus Hamiltonian is assumed to be an zero matrix.

The energy level diagram in Figure S35(c) shows multiple possible non-radiative decay channel from the ES to the GS. However, the strong correlation between the ODMR transitions of S = 1 and S = 1/2 states indicates that the transition from the metastable S = 1 state to the metastable spin pair state should dominate the non-radiative process. This implies that κ_2 is expected to be significantly larger than κ_1 and the ISC rate from S = 1 state to the GS. Additionally, we note that the reverse ISC from the S = 1 to the S = 0 ground state is expected to be much weaker than the charge transfer process. This is supported by the observation that the S = 1 ODMR contrast significantly decreases and vanishes at zero magnetic field, together with S = 1/2 ODMR, despite a ZFS of D ∼ 1 GHz. If the reverse ISC were strong, a detectable ODMR contrast for the S = 1 transition would be expected at zero field. Such weak reverse ISC rates can be theoretically expected for defects with C_{2v} symmetry.

Based on these considerations, we simplify the model to the diagram shown in Figure S41(a). The incoherent transitions between different spin manifold are introduced by Lindblad operators as listed in Table S9.

**incoherent interactions**	**transition rates**	**Value** ($\Gamma_i/2\pi$])	**lindblad operators**
Spin preserving optical pumping	Γ_p	1.5 MHz	$L_p = \|e\rangle \langle g\|$
Spin preserving radiative decay	Γ_{rad}	10 MHz	$L_{rad} = \|g\rangle \langle e\|$
Non-radiative transition from $\|e\rangle$ to $\|\pm1\rangle$	Γ_1^e	10 MHz	$L_m^e = \|m\rangle \langle e\|$, for m ∈ {+1, -1}
Non-radiative transition from $\|e\rangle$ to $\|0\rangle$	Γ_0^e	1 MHz	$L_0^e = \|0\rangle \langle e\|$
Non-radiative transition from $\|\pm1\rangle$ to $\|g\rangle$	Γ_g^1	100 Hz	$L_g^m = \|g\rangle \langle m\|$, for m ∈ {+1, -1}
Non-radiative transition from $\|0\rangle$ to $\|g\rangle$	Γ_g^0	500 Hz	$L_g^0 = \|g\rangle \langle 0\|$
Charge hopping from $\|\pm1\rangle$ to $\|T_\pm\rangle$	Γ_T^1	2 MHz	$L_T^e = \|T_\pm\rangle \langle m\|$, for m ∈ {+1, -1}
Charge hopping from $\|0\rangle$ to $\|T_\pm\rangle$	Γ_T^0	100 kHz	$L_T^e = \|T_\pm\rangle \langle 0\|$
Charge hopping from $\|\pm1\rangle$ to $\|ST_0\rangle$	Γ_S^1	200 kHz	$L_S^e = \|ST_0\rangle \langle m\|$, for m ∈ {+1, -1}
Charge hopping from $\|0\rangle$ to $\|ST_0\rangle$	Γ_S^0	2 MHz	$L_S^e = \|ST_0\rangle \langle 0\|$
Charge hopping from $\|T_\pm\rangle$ to $\|g\rangle$	Γ_g^T	160 kHz	$L_g^T = \|g\rangle \langle T_\pm\|$
Charge hopping from $\|ST_0\rangle$ to $\|g\rangle$	Γ_g^S	400 kHz	$L_g^S = \|g\rangle \langle ST_0\|$

Table S9. Construct the Lindblad operators for each of the above incoherent interactions for ODMR simulation using model 1.

The transition rates introduced above were based on the assumption that the external magnetic field is well aligned with the intrinsic quantization axis (defined by the ZFS) and that the system is far from the LAC, where the previously defined spin energy levels correspond to the eigenstates. However, when there is a finite misalignment of the magnetic field or in the presence of hyperfine, spin state mixing occurs, particularly near the LAC, leading to new eigenstates that are linear combinations of the original eigenstates:

$$|p\rangle = \sum_i \alpha_{pi}(\mathbf{B}) \left|i^0\right\rangle , \tag{31}$$

where $|i^0\rangle$ and $|p\rangle$ represent the original and new eigenstates, respectively. This state mixing alters the spin-dependent transitions, resulting in modified transition rates given by:

$$\Gamma_{pq} = \sum_{i,j} |\alpha_{pi}|^2 |\alpha_{qj}|^2 \Gamma_{ij}, \tag{32}$$

where α_{pi} and α_{qj} are the coefficients describing the contributions of the original eigenstates to the new eigenstates, and Γ_{ij} are the transition rates between the original states. The simulated ODMR spectrum is shown in Figure S41 which captures the key feature of the magnetic field dependence.

2. Model 2: GS spin pair and metastable triplet states

In the second model shown in Figure S35(d), the spin-pair manifold resides in the GS and is optically active. A laser excites the spin pair to an ES while conserving its spin state, assuming the spin energy levels in the ES are similar to those in the GS. Electrons in the ES can either decay radiatively back to the spin-pair GS or transfer to a spin S = 1 metastable state via charge hopping, resulting in different internal charge states. Because the S = 1 and S = 1/2 manifolds are strongly correlated, the dominant non-radiative pathway is ES → S = 1 state → spin pair GS. Therefore, here we neglect the S = 0 singlet manifold, assuming transitions into the singlet state are negligible compared to those into the S = 1 manifold. A schematic of this simplified model is presented in Figure S42(a).

The Hamiltonian for this model is expressed as:

$$H = H_{pair,g} \oplus H_{pair,e} \oplus H_{m,S1} = \begin{pmatrix} H_{pair,g}^{8\times8} & & \\ & H_{pair,e}^{8\times8} & \\ & & H_{m,S1}^{6\times6} \end{pmatrix}, \tag{33}$$

where $H_{m,S1}$ represent the metastable S = 1 Hamiltonians defined by Eq.30. $H_{pair,g}$ and $H_{pair,g}$ correspond to the spin-pair Hamiltonians in the GS and ES, respectively. $H_{pair,g}$ was defined by Eq.29. For simplicity, we neglect

coherent interactions in the ES due to limited information about its spin structure. This assumption does not affect the key features we aim to analyze in this model. Consequently, the ES Hamiltonian is treated as an zero matrix. Similarly, we introduce the incoherent transitions, as summarized in Table S10, and recalculate the new transition rates at different magnetic fields using Eq. 32.

**incoherent interactions**	**transition rates**	**Value** ($\Gamma_i/2\pi$])	**lindblad operators**
Spin preserving optical pumping	Γ_p	1.5 MHz	$L_p = \|e\rangle\langle g\|$
Spin preserving radiative decay	Γ_{rad}	10 MHz	$L_{rad} = \|g\rangle\langle e\|$
Charge hopping from $\|T_\pm, e\rangle$ to $\|\pm1\rangle$	$\Gamma_1^{T,e}$	10 MHz	$L_m^{T,e} = \|m\rangle\langle T_\pm, e\|$, for m $\in$ {+1, -1}
Charge hopping from $\|T_\pm, e\rangle$ to $\|0\rangle$	$\Gamma_0^{T,e}$	1 MHz	$L_0^{T,e} = \|0\rangle\langle T_\pm, e\|$
Charge hopping from $\|ST_0, e\rangle$ to $\|\pm1\rangle$	$\Gamma_1^{S,e}$	125 kHz	$L_m^{S,e} = \|m\rangle\langle ST_0, e\|$, for m $\in$ {+1, -1}
Charge hopping from $\|ST_0, e\rangle$ to $\|0\rangle$	$\Gamma_0^{S,e}$	2.4 MHz	$L_0^{S,e} = \|0\rangle\langle ST_0, e\|$
Charge hopping from $\|\pm1\rangle$ to $\|T_\pm, g\rangle$	$\Gamma_{T,g}^1$	5 MHz	$L_{T,g}^m = \|T_\pm, g\rangle\langle m\|$, for m $\in$ {+1, -1}
Charge hopping from $\|0\rangle$ to $\|T_\pm, g\rangle$	$\Gamma_{T,g}^0$	1 MHz	$L_{T,g}^0 = \|T_\pm, g\rangle\langle 0\|$
Charge hopping from $\|\pm1\rangle$ to $\|ST_0, g\rangle$	$\Gamma_{S,g}^1$	200 kHz	$L_{ST_0,g}^m = \|ST_0, g\rangle\langle m\|$, for m $\in$ {+1, -1}
Charge hopping from $\|0\rangle$ to $\|ST_0, g\rangle$	$\Gamma_{S,g}^0$	2.1 MHz	$L_{ST_0,g}^0 = \|ST_0, e\rangle\langle 0\|$

Table S10. Construct the Lindblad operators for each of the above incoherent interactions for ODMR simulation using model 2.

The simulated ODMR spectrum using Model 2 also exhibits a clear LAC near 35 mT, as shown in Figure S42. Additionally, a noticeable decrease in the ODMR contrast of the spin pair is observed near 35 mT, which originates from state mixing within the metastable manifold. Both features align well with the experimental observations of Group III ODMR, as shown in Figure 1 in the main text and Figure S12(c). Furthermore, the imbalance in the hyperfine structure indicates a net nuclear spin polarization of the ^{13}C nuclei detected by the spin pair. This nuclear spin polarization occurs only near 35 mT (LAC of spin S = 1), and thus it is mediated through hyperfine interactions with the spin S = 1 manifold, further corroborating the experimental findings for Group III defects.

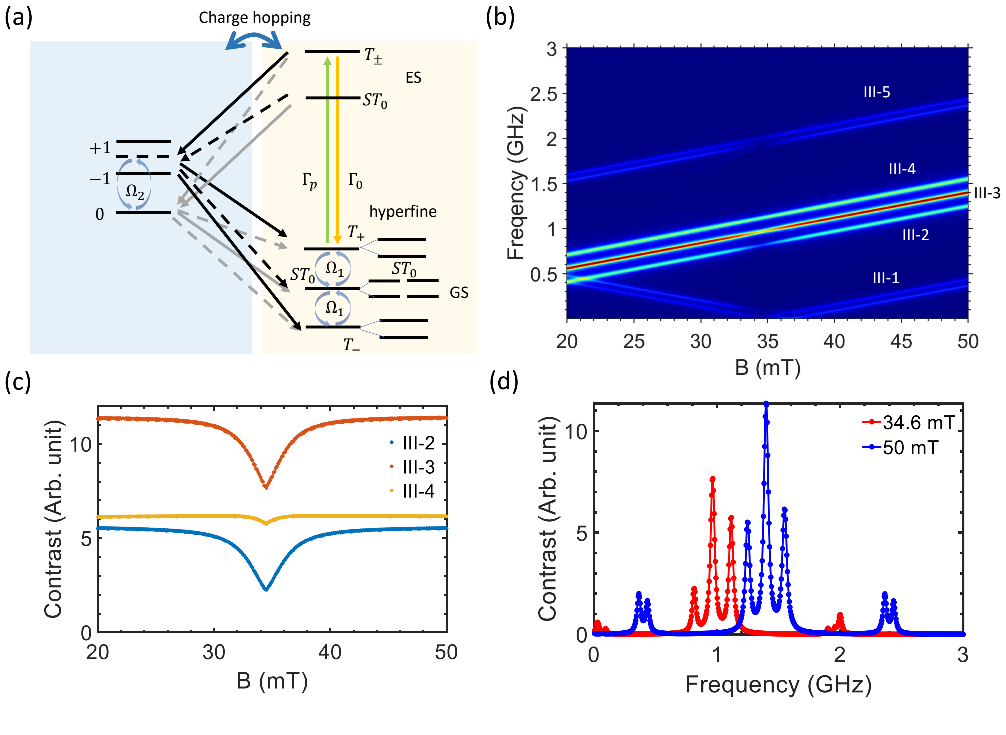

Figure S42. (a) Energy level diagram of model 2 for simulation. (b) Simulated ODMR as a function of B field. (c) Simulated ODMR contrast of III-3 as a function of B field. The small variation is due to the finite frequency resolution in the simulation. (d) Two ODMR examples considering the magnetic field at LAC (34.6 mT) and far from LAC (50 mT).

Discussion Both models successfully capture key experimental features, including the reduction in ODMR contrast at the LAC, the hyperfine structure, and the interplay between the S = 1/2 and S = 1 manifolds. The primary difference between the two models lies in the location of the spin-pair charge state—Model 1 assumes a singlet GS with a metastable spin-pair state, while Model 2 assumes a spin-pair GS with a metastable triplet state.

Although both models capture the key features of ODMR behaviors, Model 1 appears more likely overall. This is because most defects exhibit pronounced asymmetry in their Rabi oscillations—a feature that is best explained by a metastable spin configuration and is not readily accounted for by ground-state spins alone.

Building on the discussion of the spin-pair model and our DFT calculations of candidate defect structures, we can propose a more complete picture of the possible defect configurations. The spin-pair model involves two closely spaced defects—one acting as a donor and the other as an acceptor. Our DFT calculations of C_BC_N-DAP (for Group II) and C_BO_N (for Group III) assume single-defect configurations and thus identify only one member of the defect complex. The second defect, whether donor or acceptor, remains unidentified, due to the absence of a distinct hyperfine signature. Further identification of both defect constituents may be possible by using high-purity hBN to suppress intrinsic ^{12}C impurities, combined with higher ^{13}C doping density. This approach would increase the likelihood that both Defect A and Defect B are coupled to host ^{13}C nuclear spins, enabling clearer spectroscopic identification of both members in the spin pair.

For Group II defects, we assign the C_BC_N-DAP to be the candidate as one of the defect pair members (Defect B), where a ^{13}C atom resides at the C_N site. In the positively charged state ($C_B^+C_N^0$), this defect exhibits an S = 1/2 spin configuration. We propose that it is weakly coupled to a nearby S = 1/2 spin center (Defect A), located a few nanometers away, forming a spin pair. Upon accepting an electron from Defect A, $C_B^+C_N^0$ becomes neutral ($C_B^0C_N^0$), leading both defects to adopt S = 0 singlet ground states. One of these defects, more like to be Defect A, is optically active and hosts a metastable S = 1 state. Since no hyperfine structure is observed for the S = 1 transitions, we attribute the hyperfine interaction to the S = 1/2 state of Defect B and the S = 1 manifold to Defect A.

Similarly, for Group III defects, the C_BO_N structure accounts well for the large observed hyperfine splitting. In its positively charged state ($C_B^0O_N^+$), this defect exhibits an S = 1/2 spin and is likely weakly coupled to another nearby unidentified S = 1/2 defect, forming a similar spin pair. When $C_B^0O_N^+$ accepts an electron from the adjacent defect, it transitions to the neutral charge state ($C_B^0O_N^0$), expected to be a singlet. Notably, the S = 1 state observed in Defect 3 shows clear hyperfine coupling with a ^{13}C nuclear spin, and this same nuclear spin is also coupled to the S = 1/2 state in $C_B^0O_N^+$ (suggested by the nuclear spin dynamical polarization via S = 1 LAC). These observations suggest that the singlet ground state likely hosts a metastable S = 1 state that retains coupling to the ^{13}C nucleus, while the second spin defect in the pair is optically inactive.

These results highlight the importance of charge dynamics in determining the spin properties of quantum emitters in hBN. Future experiments, such as charge-state-selective spectroscopy and single-shot readout, could provide further insights into the microscopic origins of these spin-pair states and their potential applications in quantum sensing and information processing.

To further improve the reliability of theoretical predictions, it will be important to benchmark against higher levels of theory beyond standard DFT. On one hand, higher-level approaches such as Green's function methods GW/BSE[19, 29], quantum defect embedding theory[30], or quantum Monte Carlo[31] can offer more accurate predictions of excitation energies, oscillator strengths, and radiative lifetimes by better accounting for static and dynamical correlations. More importantly, a deeper understanding of spin–photodynamics, particularly charge dynamics between paired defects, can guide DFT-based modeling. This mechanism is distinct from conventional single-defect spin centers like NV centers in diamond. In this context, quantum kinetic theory based on density-matrix formalism[32–34], time-dependent DFT [25], which can capture non-equilibrium charge and spin dynamics, may play an essential role in future efforts to model these two-defect spin pair systems more realistically.

[1] Zhao Mu, Hongbing Cai, Disheng Chen, Jonathan Kenny, Zhengzhi Jiang, Shihao Ru, Xiaodan Lyu, Teck Seng Koh, Xiaogang Liu, Igor Aharonovich, et al. Excited-state optically detected magnetic resonance of spin defects in hexagonal boron nitride. Physical Review Letters, 128:216402, 2022.

[2] Nikhil Mathur, Arunabh Mukherjee, Xingyu Gao, Jialun Luo, Brendan A McCullian, Tongcang Li, A Nick Vamivakas, and Gregory D Fuchs. Excited-state spin-resonance spectroscopy of V_B^- defect centers in hexagonal boron nitride. Nature Communications, 13:3233, 2022.

[3] Hannah L Stern, Carmem M. Gilardoni, Qiushi Gu, Simone Eizagirre Barker, Oliver FJ Powell, Xiaoxi Deng, Stephanie A Fraser, Louis Follet, Chi Li, Andrew J Ramsay, et al. A quantum coherent spin in hexagonal boron nitride at ambient conditions. Nature Materials, 23(10):1379–1385, 2024.

[4] Xingyu Gao, Sumukh Vaidya, Kejun Li, Peng Ju, Boyang Jiang, Zhujing Xu, Andres E Llacsahuanga Allcca, Kunhong Shen, Takashi Taniguchi, Kenji Watanabe, et al. Nuclear spin polarization and control in hexagonal boron nitride. Nature Materials, 21:1024–1028, 2022.

[5] Mo Chen, Masashi Hirose, Paola Cappellaro, et al. Measurement of transverse hyperfine interaction by forbidden transitions. Physical Review B, 92:020101, 2015.

[6] David A Hopper, Henry J Shulevitz, and Lee C Bassett. Spin readout techniques of the nitrogen-vacancy center in diamond. Micromachines, 9:437, 2018.

[7] Philipp Auburger and Adam Gali. Towards ab initio identification of paramagnetic substitutional carbon defects in hexagonal boron nitride acting as quantum bits. Physical Review B, 104:075410, 2021.

[8] Paolo Giannozzi, Stefano Baroni, Nicola Bonini, Matteo Calandra, Roberto Car, Carlo Cavazzoni, Davide Ceresoli, Guido L Chiarotti, Matteo Cococcioni, Ismaila Dabo, Andrea Dal Corso, Stefano de Gironcoli, Stefano Fabris, Guido Fratesi, Ralph Gebauer, Uwe Gerstmann, Christos Gougoussis, Anton Kokalj, Michele Lazzeri, Layla Martin-Samos, Nicola Marzari, Francesco Mauri, Riccardo Mazzarello, Stefano Paolini, Alfredo Pasquarello, Lorenzo Paulatto, Carlo Sbraccia, Sandro Scandolo, Gabriele Sclauzero, Ari P Seitsonen, Alexander Smogunov, Paolo Umari, and Renata M Wentzcovitch. QUANTUM ESPRESSO: A Modular and Open-Source Software Project for Quantum Simulations of Materials. J. Phys.: Condens. Matter, 21(39):395502, sep 2009.

[9] Jochen Heyd, Gustavo E Scuseria, and Matthias Ernzerhof. Hybrid Functionals Based on a Screened Coulomb Potential. J. Chem. Phys., 118(18):8207–8215, 2003. doi:10.1063/1.1564060.

[10] Jochen Heyd, Gustavo E. Scuseria, and Matthias Ernzerhof. Erratum: "Hybrid functionals based on a screened Coulomb potential" [J. Chem. Phys. 118, 8207 (2003)]. The Journal of Chemical Physics, 124(21):219906, 06 2006. ISSN 0021-9606. doi:10.1063/1.2204597.

[11] D. R. Hamann. Optimized Norm-Conserving Vanderbilt Pseudopotentials. Phys. Rev. B, 88(8):085117, aug 2013.

[12] Martin Schlipf and François Gygi. Optimization Algorithm for the Generation of ONCV Pseudopotentials. Comput. Phys. Commun., 196:36–44, 2015.

[13] Christian Tantardini, Alexander G Kvashnin, and Davide Ceresoli. Gipaw pseudopotentials of d elements for solid-state nmr. Mater., 15(9):3347, 2022.

[14] Tyler J Smart, Kejun Li, Junqing Xu, and Yuan Ping. Intersystem Crossing and Exciton–Defect Coupling of Spin Defects in Hexagonal Boron Nitride. npj Comput. Mater., 7(1):1–8, 2021. doi:10.1038/s41524-021-00525-5.

[15] M Mackoit-Sinkevičienė, Marek Maciaszek, Chris G Van de Walle, and Audrius Alkauskas. Carbon Dimer Defect as a Source of the 4.1 eV Luminescence in Hexagonal Boron Nitride. Appl. Phys. Lett., 115(21):212101, 2019.

[16] Nicola Varini, Davide Ceresoli, Layla Martin-Samos, Ivan Girotto, and Carlo Cavazzoni. Enhancement of dft-calculations at petascale: nuclear magnetic resonance, hybrid density functional theory and car–parrinello calculations. Comput. Phys. Commun., 184(8):1827–1833, 2013.

[17] He Ma, Marco Govoni, and Giulia Galli. PyZFS: A Python Package for First-Principles Calculations of Zero-Field Splitting Tensors. J. Open Source Softw., 5(47):2160, 2020. doi:10.21105/joss.02160.

[18] Ravishankar Sundararaman and Yuan Ping. First-principles Electrostatic Potentials for Reliable Alignment at Interfaces and Defects. J. Chem. Phys., 146(10):104109, 2017. doi:10.1063/1.4978238.

[19] Feng Wu, Andrew Galatas, Ravishankar Sundararaman, Dario Rocca, and Yuan Ping. First-Principles Engineering of Charged Defects for Two-Dimensional Quantum Technologies. Phys. Rev. Mater., 1(7):071001(R), 2017.

[20] R. Sundararaman, K. Letchworth-Weaver, K. A. Schwarz, D. Gunceler, Y. Ozhabes, and T. A. Arias. JDFTx: Software for Joint Density-Functional Theory. SoftwareX, 6:278–284, 2017.

[21] Chris G Van de Walle and Peter E Bloechl. First-principles calculations of hyperfine parameters. Phys. Rev. B, 47(8): 4244, 1993.

[22] Mohammad Saeed Bahramy, Marcel HF Sluiter, and Yoshiyuki Kawazoe. Pseudopotential hyperfine calculations through perturbative core-level polarization. Phys. Rev. B, 76(3):035124, 2007.

[23] NJ Stone. Table of nuclear magnetic dipole and electric quadrupole moments. At. Data Nucl. Data Tables, 90(1):75–176, 2005.

[24] MJ Rayson and PR Briddon. First Principles Method for the Calculation of Zero-Field Splitting Tensors in Periodic Systems. Phys. Rev. B, 77(3):035119, 2008. doi:10.1103/PhysRevB.77.035119.

[25] Islay O Robertson, Benjamin Whitefield, Sam C Scholten, Priya Singh, Alexander J Healey, Philipp Reineck, Mehran Kianinia, Gergely Barcza, Viktor Ivády, David A Broadway, Igor Aharonovich, and Jean-Philippe Tetienne. A charge transfer mechanism for optically addressable solid-state spin pairs. arXiv preprint arXiv:2407.13148, 2024.

[26] Raj N Patel, Rebecca EK Fishman, Tzu-Yung Huang, Jordan A Gusdorff, David A Fehr, David A Hopper, S Alex Breitweiser, Benjamin Porat, Michael E Flatté, and Lee C Bassett. Room temperature dynamics of an optically addressable single spin in hexagonal boron nitride. Nano Letters, 2024.

[27] Norihito Kosugi, Shigemasa Matsuo, Kohkichi Konno, and Noriyuki Hatakenaka. Theory of damped rabi oscillations. Physical Review B—Condensed Matter and Materials Physics, 72(17):172509, 2005.

[28] Francesco Campaioli, Jared H Cole, and Harini Hapuarachchi. Quantum master equations: Tips and tricks for quantum optics, quantum computing, and beyond. PRX Quantum, 5(2):020202, 2024.

[29] Yuan Ping and Tyler J Smart. Computational design of quantum defects in two-dimensional materials. Nature Computational Science, 1:646–654, 2021.

[30] He Ma, Nan Sheng, Marco Govoni, and Giulia Galli. Quantum embedding theory for strongly correlated states in materials. Journal of Chemical Theory and Computation, 17:2116–2125, 2021.

[31] KA Simula and Ilja Makkonen. Calculation of the energies of the multideterminant states of the nitrogen vacancy center in diamond with quantum monte carlo. Physical Review B, 108:094108, 2023.

[32] Jacopo Simoni, Gabriele Riva, and Yuan Ping. First-principles open quantum dynamics for solids based on density-matrix formalism. arXiv preprint arXiv:2504.17936, 2025.

[33] Junqing Xu, Adela Habib, Ravishankar Sundararaman, and Yuan Ping. Ab initio ultrafast spin dynamics in solids. Physical Review B, 104:184418, 2021.

[34] Junqing Xu and Yuan Ping. Ab initio predictions of spin relaxation, dephasing, and diffusion in solids. Journal of Chemical Theory and Computation, 20:492–512, 2023.
